# Distribution of infectious and parasitic agents among three sentinel bee species across European agricultural landscapes

**DOI:** 10.1038/s41598-024-53357-w

**Published:** 2024-02-12

**Authors:** Aurélie Babin, Frank Schurr, Sabine Delannoy, Patrick Fach, Minh Huyen Ton Nu Nguyet, Stéphanie Bougeard, Joachim R. de Miranda, Maj Rundlöf, Dimitry Wintermantel, Matthias Albrecht, Eleanor Attridge, Irene Bottero, Elena Cini, Cecilia Costa, Pilar De la Rúa, Gennaro Di Prisco, Christophe Dominik, Daniel Dzul, Simon Hodge, Alexandra-Maria Klein, Jessica Knapp, Anina C. Knauer, Marika Mänd, Vicente Martínez-López, Piotr Medrzycki, Maria Helena Pereira-Peixoto, Simon G. Potts, Risto Raimets, Oliver Schweiger, Deepa Senapathi, José Serrano, Jane C. Stout, Giovanni Tamburini, Mark J. F. Brown, Marion Laurent, Marie-Pierre Rivière, Marie-Pierre Chauzat, Eric Dubois

**Affiliations:** 1 ANSES, Sophia Antipolis Laboratory, Unit of Honey bee Pathology, 06902 Sophia Antipolis, France; 2 IdentyPath Genomics Platform, Food Safety Laboratory, ANSES, 94701 Maisons-Alfort, France; 3 Paris-Est University, ANSES, Laboratory for Animal Health, 94701 Maisons-Alfort, France; 4 ANSES, Ploufragan-Plouzané-Niort Laboratory, Epidemiology and Welfare, France; 5 Department of Ecology, Swedish University of Agricultural Sciences, 75007 Uppsala, Sweden; 6 Department of Biology, Lund University, Lund, Sweden; 7 Chair of Nature Conservation and Landscape Ecology, University of Freiburg, Tennenbacher Straße 4, 79106 Freiburg, Germany; 8 Agroecology and Environment, Agroscope, Reckenholzstrasse 191, 8046 Zurich, Switzerland; 9 Federation of Irish Beekeepers’ Associations, Tullamore, Ireland; 10 Botany, School of Natural Sciences, Trinity College Dublin, Dublin, Ireland; 11 Centre for Agri-Environmental Research, School of Agriculture, Policy and Development, University of Reading, Reading, UK; 12 CREA Research Centre for Agriculture and Environment, Via di Corticella 133, 40128 Bologna, Italy; 13 Department of Zoology and Physical Anthropology, Faculty of Veterinary, University of Murcia, 30100 Murcia, Spain; 14 Institute for Sustainable Plant Protection, The Italian National Research Council, Piazzale E. Ferni 1, 80055 Portici, Napoli, Italy; 15 UFZ-Helmholtz Centre for Environmental Research, Department of Community Ecology, 06120 Halle, Germany; 16 German Centre for Integrative Biodiversity Research (iDiv) Halle-Jena-Leipzig, Puschstraße 4, 04103 Leipzig, Germany; 17 School of Agriculture and Food Science, University College Dublin, Dublin, Ireland; 18 Institute of Agricultural and Environmental Sciences, Estonian University of Life Sciences, Tartu, Estonia; 19 Department of Evolution, Ecology and Behaviour, Institute of Infection, Veterinary and Ecological Sciences, University of Liverpool, Crown Street, Bioscience Building, L69 7ZB, Liverpool, UK; 20 University of Bari, Department of Soil, Plant and Food Sciences (DiSSPA-Entomology and Zoology), Bari, Italy; 21 Centre for Ecology, Evolution & Behaviour, Department of Biological Sciences, School of Life Sciences and the Environment, Royal Holloway University of London, Egham, UK

**Keywords:** Biodiversity, Evolutionary ecology, Biodiversity, Evolutionary ecology, Pathogens, Infectious-disease diagnostics

## Abstract

Infectious and parasitic agents (IPAs) and their associated diseases are major environmental stressors that jeopardize bee health, both alone and in interaction with other stressors. Their impact on pollinator communities can be assessed by studying multiple sentinel bee species. Here, we analysed the field exposure of three sentinel managed bee species (*Apis mellifera*, *Bombus terrestris* and *Osmia bicornis*) to 11 IPAs (six RNA viruses, two bacteria, three microsporidia). The sentinel bees were deployed at 128 sites in eight European countries adjacent to either oilseed rape fields or apple orchards during crop bloom. Adult bees of each species were sampled before their placement and after crop bloom. The IPAs were detected and quantified using a harmonised, high-throughput and semi-automatized qPCR workflow. We describe differences among bee species in IPA profiles (richness, diversity, detection frequencies, loads and their change upon field exposure, and exposure risk), with no clear patterns related to the country or focal crop. Our results suggest that the most frequent IPAs in adult bees are more appropriate for assessing the bees’ IPA exposure risk. We also report positive correlations of IPA loads supporting the potential IPA transmission among sentinels, suggesting careful consideration should be taken when introducing managed pollinators in ecologically sensitive environments.

## Introduction

Managed and wild bees contribute crucial pollination services to cultivated and wild plants and, in the case of managed honey bees, provide beneficial products (honey, beeswax, pollen)^1,2^. However, bee health has been dramatically jeopardized over the last decades, with massive losses of managed honey bee colonies and a decline of wild bee populations^3,4^. The integrity of bee health relies on dealing with multiple interacting abiotic and biotic environmental stressors, some of them arising from anthropogenic ecosystem modifications via agricultural intensification and urbanisation^4,5^. Habitat loss and fragmentation, use of agrochemicals and other pollutants, low floral resource diversity, quality and quantity, as well as infectious and parasitic agents (IPAs) and their associated diseases, are major interacting threats to bee health at the individual and population levels, whose effects can be exacerbated by climate change^3,4,6–8^.

Many different pollinators nest and forage in the same landscapes^9,10^, and are thus exposed to similar landscape-level environmental stressors. Yet pollinator-specific traits (such as sociality, colony size, foraging range and dietary preferences) can significantly modulate both the exposure to these stressors^11^ and the effects of any exposure on bee health, as modulated by species-specific differences in individual tolerance and resistance mechanisms, as well as social immunity mechanisms for eusocial and semi-social bees^12,13^. It is therefore critical that different types of bee species are deployed as sentinels (or bioindicators) for monitoring the landscape-level environmental stressors in order to have a full representation of the breadth and depth of wild bee species, most of whom have a solitary lifestyle and are therefore poorly represented by social bee species^14^. Managed honey bees and their pollen and nectar have long been used as environmental sentinels to monitor, for instance, the exposure to heavy metal and agrochemical contaminants, pathogens of crop plants and bees, as well as to monitor changes in agricultural landscapes and the consequences of climate change ^14–17^. Bumble bees and certain solitary bees are increasingly considered relevant and promising sentinels for the wider community of semi-social and solitary bees in environmental risk assessment of pesticides^18–22^, and the interacting effects of landscape complexity and agricultural management regime^21,23–26^. Wild bee species richness, diversity and abundance have also been used as bioindicators for heavy metal and pesticide pollution^27,28^, and local habitat quality for pollinators and landscape structure^29,30^.

Within the European project PoshBee^31^, three different sentinel bee species (*Apis mellifera*, *Bombus terrestris* and *Osmia bicornis*) were deployed in a huge network of 128 field sites in eight European countries (Estonia, Germany, Ireland, Italy, UK, Spain, Sweden, Switzerland) covering four biogeographical zones (Atlantic, Boreal, Continental, Mediterranean) for environmental monitoring, sample collection and measurement of multiple chemical, biological and pathological traits relevant to bee health and landscape-level stressors on two, economically important, bee-attractive mass flowering crops (oilseed rape and apple)^32^. In this study, we focused on the detection and quantification of the presence of 11 IPAs in the three sentinel bee species, as biomarkers for bee health, before the bee’s deployment in the field sites and after they had been exposed to the field site conditions for several weeks. These IPAs cover the main (honey) bee diseases in Europe: six RNA viruses ((*Acute bee paralysis virus* (ABPV), *Black queen cell virus* (BQCV), Chronic bee paralysis virus (CBPV), both A and B genotypes of *Deformed wing virus*^12^, and *Sacbrood virus* (SBV); the bacteria responsible for American foulbrood (AFB, caused by *Paenibacillus larvae*) and European foulbrood (EFB, caused primarily by *Melissococcus plutonius*) and three microsporidian parasites (the honey bee parasites *Nosema apis* and *Nosema ceranae*, and the bumble bee parasite *Nosema bombi* (a reassignment of these three parasites to the genus *Vairimorpha* has been proposed recently but is currently under debate)^33^.

Our study analysed specifically the local co-distribution of the 11 IPAs in the three sentinel bees (IPA richness, diversity, exposure risk, detection frequencies and loads), as a function of the bee species, exposure time, focal crop, and country/biogeographical zone, and identify and quantify any possible IPA transmission between bee species. Like all organisms, bees naturally interact with a wide range of IPAs (viruses, bacteria, eukaryotic parasites) they have coevolved with, which have specific effects on some aspects of the bee’s life cycle and behaviour^34^. Some IPAs can become pathogenic when present at excessive levels and many of them, particularly the viruses^34^ can infect multiple hosts more or less successfully and can be transmitted between bee species^35^. This transmission constitutes an environmental risk per se, related to the interactions between bee species. In addition, the interaction with the landscape and its resources can constitute landscape-level stressors which can also affect the distribution and loads of individual IPAs and thus influence their transmission risk. For example, co-exposure to neonicotinoid pesticides with the microsporidium *N. ceranae* increases the mortality of managed honey bees *Apis mellifera*^36^, while co-exposure with the *Deformed wing virus* (DWV) alters their biological programme of labour division and the foragers’ cognitive ability to return to the hive^37^.

IPA infection intensity of managed and wild bees varies naturally throughout the season^12,13,34﻿^ and is also affected by available floral resources^38–44^, climatic conditions^45–48^, habitat quality^44^, urbanisation intensity^49–51^, density of managed bees^44,52^ and interacting environmental factors^53–55^. The specific and temporary deployment of large numbers of managed honey bees, bumble bees or solitary bees for crop pollination may therefore represent a threat to native wild bee populations, through competition for limited floral resources and nest sites ^9,56,57^, and through IPA transmission in overlapping foraging networks^8,58–63^ or direct contact between bees or their faeces. The health management, placement and density of managed bees are therefore key factors in the potential IPA risk to native wild bees^4,56,64–70^. Other important factors in this IPA exposure risk are the host range and the IPA infectivity in different bee species, the decay of IPA infectivity in the open environment^63^, the susceptibility, tolerance and resistance of the bee species to the shared IPAs, and the degree of overlap in the foraging and nesting requirements of the different bee species during the entire season.

To our knowledge, our study is the first European-wide qualitative and quantitative description of IPA profiles of three co-located sentinel bee species under real-world field conditions during the blooming of two bee-attractive crops, with respect to the site network structure and bee interactions and traits. It adds to the recent studies at national scale in several countries, and at the Europe scale with fewer sampling sites, which measured the detection frequencies and loads of multiple viruses and parasites in managed honey bees and bumble bees, and in wild bees^12,13,48,60^.

## Results

### Variation in IPA richness, diversity and detection frequencies among bee species

**Figure 1:**
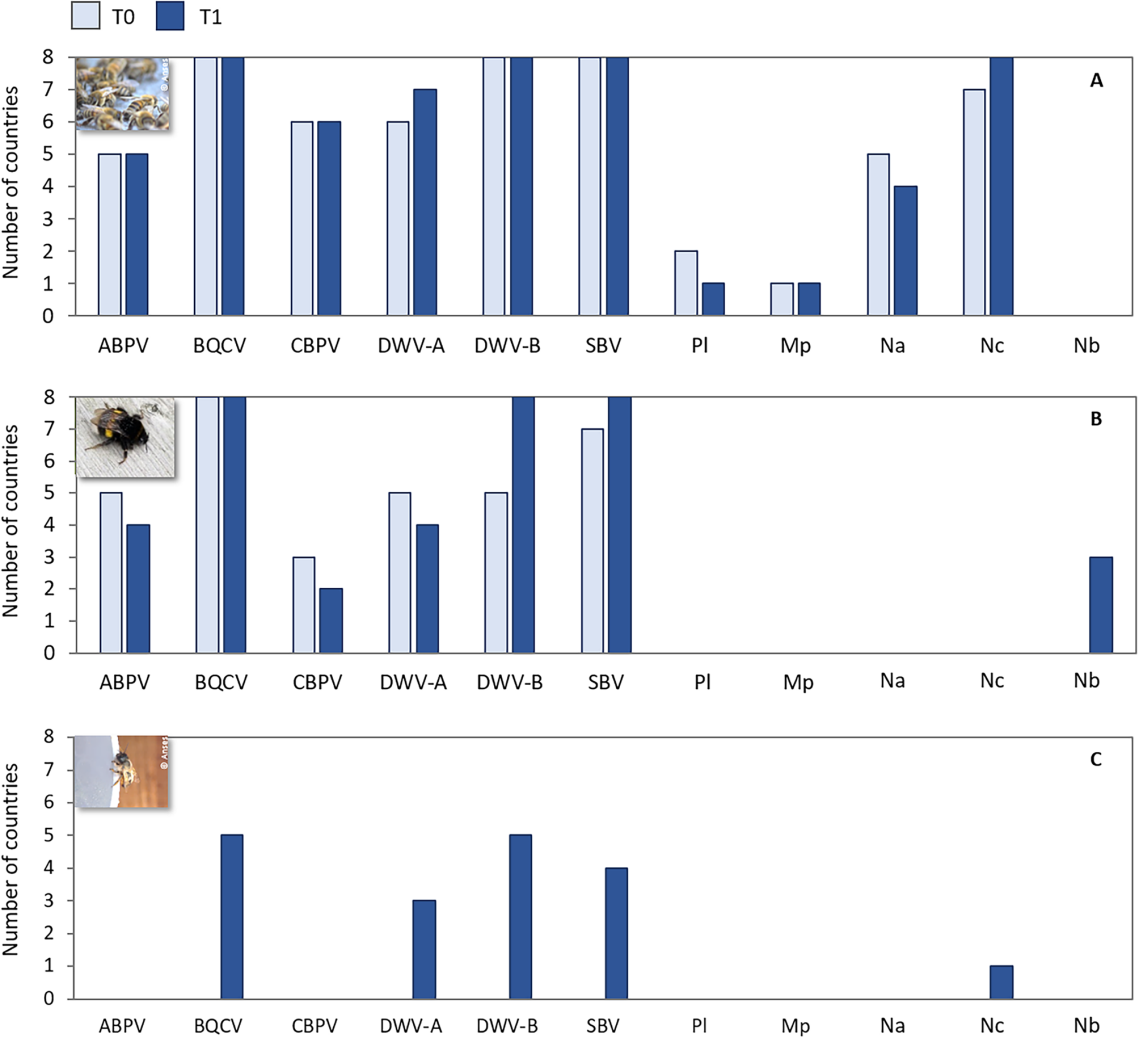
Numbers of countries with at least one positive site for each IPA at the two screening occasions (T0, T1). (**A**) *Apis mellifera*, (**B**) *Bombus terrestris*, and (**C**) *Osmia bicornis*. Honey bees and bumble bees were deployed in all the eight countries, while mason bees were deployed in six countries. Pl, *Paenibacillus larvae*; Mp, *Melissococcus plutonius*; Na, *Nosema apis*; Nc, *Nosema ceranae*; Nb, *Nosema bombi*. Numbers of countries: *A. mellifera N* = 8, *B. terrestris N* = 8, *O. bicornis N* = 5.

Overall (considering both the screening T0 before the bee’s deployment and the screening T1 after their exposure to field conditions for several weeks), each of the ten honey bee IPAs (excluding the bumble bee parasite *N. bombi*) was detected in at least one country in the *Apis mellifera* colonies, sourced locally within each country ^32^ (Fig. 1A; Supplementary table S1). The IPA richness (*i.e.* the number of IPAs detected) was not different among countries and between screening occasions (Supplementary tabless S2, S3; one-sample Mann–Whitney test on richness change *P* = 0.93), with the Irish colonies exhibiting overall the lowest richness. The detection frequencies in *A. mellifera* varied among countries for each IPA, especially for the less frequent ones, and did not differ between screening occasions (Supplementary tablesS1, S2). Viruses were the most frequent IPAs in this sentinel bee, especially BQCV, DWV-B and SBV (Supplementary table S1). Of the two honey bee microsporidia, *N. ceranae* was the most frequent while *N. apis* detection frequency was moderate across countries (Fig. 1A; Supplementary table S1), with highly variable numbers of positive sites (Supplementary table S2). After field exposure (T1), the rate of honey bee co-infection by *N. apis* and *N. ceranae* was moderate, at 16.7%. The bacterial agents causing AFB and EFB were rarely detected (Supplementary table S1). *Paenibacillus larvae* (AFB) was detected at a single Estonian site at T0 and a single Irish site at T0 and T1. In all cases, overt AFB symptoms, which is the current criterion used by the EU regulatory framework for controlling and suppressing the disease incidence^71^, were not observed. *Melissococcus plutonius* (primary agent of EFB) was detected in colonies of the same local origin, deployed at most of the oilseed rape sites in the United Kingdom for both screening occasions, but not at any of the apple sites even if oilseed rape was also grown in these landscapes^32^ (Fig. 1A; Supplementary table S2). In honey bees, the IPA diversity, estimated by the Shannon index, was moderate and it varied across countries as did the IPA sets. The index remained stable between screening occasions, mirroring the lack of significant change in IPA detection frequencies (Supplementary table S4).

In the *Bombus terrestris* colonies, sourced from local retailers but all originating from the same commercial producer^32^, the IPAs detected included mainly the six viruses, each of which was detected in at least one country and on both screening occasions, as well as the bumble bee microsporidium *N. bombi* in a few samples at T1 (Fig. 1B). Viral richness in bumble bees increased between screening occasions for all the countries except Ireland and Italy (Supplementary tables S3, S5; one-sample Mann–Whitney test on richness change *P* < 0.0001). Even so, at T1 the IPA richness in *B. terrestris* colonies was lower than that in the co-located *A. mellifera* colonies (Supplementary fig. S1). The detection frequencies of ABPV and CBPV decreased between screening occasions while those of the other viruses and *N. bombi* increased, although not significantly for all of them (Supplementary table S1). The parasite *N. bombi* was detected only after field exposure at four of the 128 sites and for both focal crops: two apple sites in Sweden and one oilseed rape site each in the United Kingdom and Germany (Fig. 1B; Supplementary table S5). Overall, the IPA diversity was lower in *B. terrestris* than in *A. mellifera* (Supplementary table S4). The variation in diversity among countries reflected the variation in detection frequencies among IPAs on both screening occasions (Supplementary tables S4, S5). After field exposure (T1), the increasing detection frequencies in *B. terrestris* yielded a decreasing heterogeneity of the IPA sets, resulting in a decreasing IPA diversity.

Before field deployment (T0), none of the screened IPAs were detected in *O. bicornis*, which were sourced from a single commercial supplier^32^. After field exposure (T1), IPA richness increased (Supplementary table S3; one-sample Mann–Whitney test of richness change *P* < 0.0001), although to lower levels than in the co-located *A. mellifera* colonies and to some extent also the *B. terrestris* colonies (Supplementary fig. S1). The IPAs found included mainly four of the six viruses (BQCV, the A and B genotypes of DWV, and SBV) with varying detection frequencies among countries, while *N. ceranae* was detected only once in Estonia (Fig. 1C; Supplementary tables S1, S6). The IPA diversity varied among countries, with an overall diversity similar to that of *B. terrestris* (Supplementary table S4). For the three sentinel bee species, the IPA richness and detection frequencies at T1 were not associated in any clear pattern with the focal crop (apple or oilseed rape), even if frequencies in *B. terrestris* were slightly higher on apple for several IPAs (Supplementary tables S7, S8, S9).

### Frequency distribution and threshold of IPA loads

**Figure 2:**
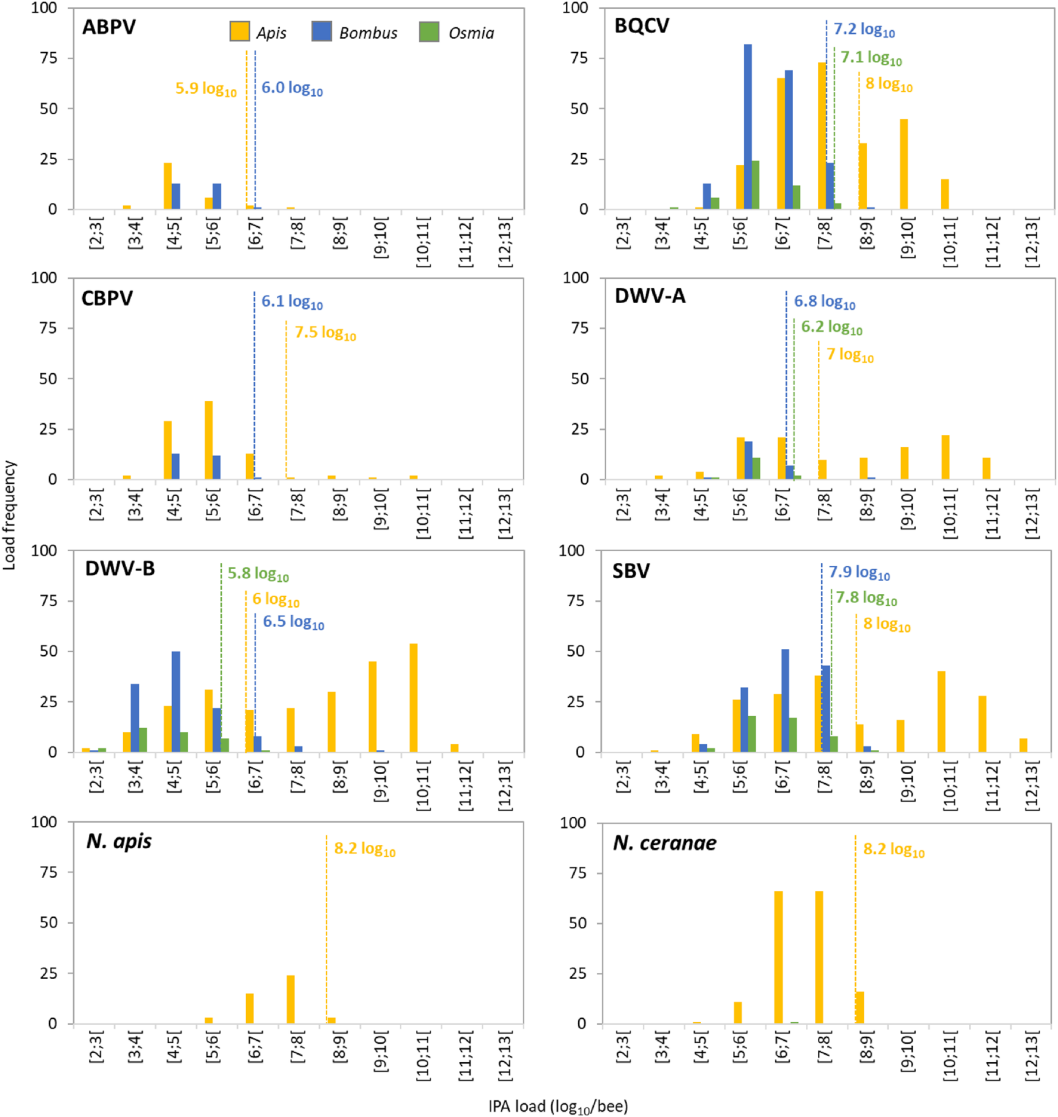
Distribution of frequencies of IPA loads (in log_10_/bee) measured in the three bee species for the eight most frequently detected IPAs (ABPV, BQCV, CBPV, DWV-A, DWV-B, SBV, and the two honey bee microsporidia, *Nosema apis* and *N. ceranae*). *Apis mellifera* (in yellow) and *Bombus terrestris* (in blue) data from the two screening occasions (T0 and T1) were pooled. *Osmia bicornis* data at T1 only (in green) are represented, since none of the IPAs was detected at T0. The load thresholds that separate the low loads (less likely to be associated with disease symptoms) from higher loads (more likely to be associated with disease symptoms) are indicated with coloured values and vertical dashed lines.

For the most prevalent IPAs detected in each sentinel bee species, the distribution of the frequencies of IPA loads, pooled across screening occasions, enabled us to calculate a threshold for each IPA for separating samples with low loads (and less likely to be associated with disease symptoms) from those with higher loads (and more likely to be associated with symptoms)^72^. For *A. mellifera*, the load frequency distributions of BQCV, DWV-A, DWV-B and SBV conformed to a bimodal distribution, with two peaks (modes). The threshold for separating the two modes was calculated as the lowest IPA load between the distribution modes (Fig. 2). The sample proportions exhibiting loads above these thresholds, *i.e.* for which colonies were more likely to exhibit disease symptoms, were 17%, 59%, 73% and 50%, for BQCV, DWV-A, DWV-B and SBV respectively. The distribution of the load frequencies for ABPV, CBPV, *N. apis* and *N. ceranae* was unimodal, and the threshold was calculated to identify the 5% highest IPA loads (Fig. 2). For both *B. terrestris* and *O. bicornis*, the frequency distributions of BQCV, DWV-A, DWV-B, and SBV loads conformed to a unimodal distribution (Fig. 2). For both bee species, the distributions aligned with the lower half of the *A. mellifera* distributions, indicating overall lower IPA loads in these two bee species. The load thresholds for these two bee species were slightly lower than for *A. mellifera*, except for ABPV and DWV-B in *B. terrestris* (Fig. 2).

### IPA exposure risk of sentinel bees

**Figure 3:**
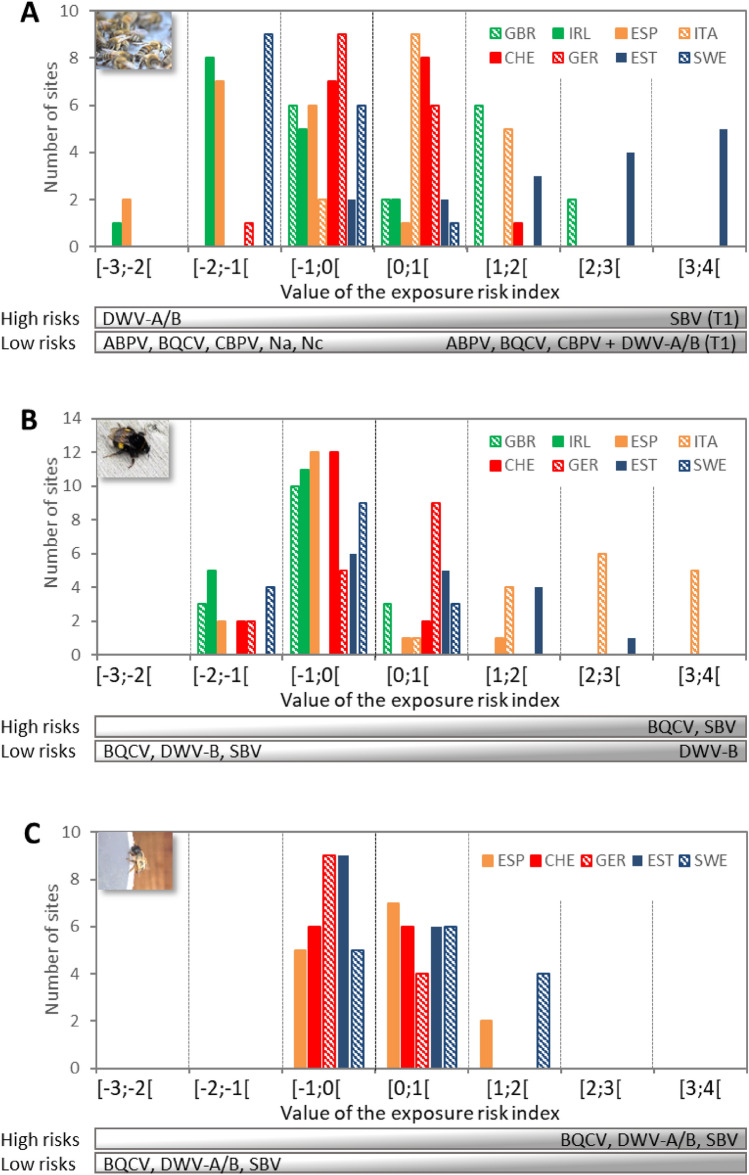
Distribution of frequencies of exposure risk values (**A**) in *Apis mellifera*, (**B**) in *Bombus terrestris* and (**C**) in *Osmia bicornis* for each country. Colours distinguish the biogeographical areas: green = Oceanic, orange = Mediterranean, red = Continental, blue = Boreal. CHE, Switzerland; ESP, Spain; EST, Estonia; GBR, United Kingdom; GER, Germany; IRL, Ireland; ITA, Italy and SWE, Sweden

Using the load thresholds, we assessed the exposure risk to the frequently detected IPAs of each sentinel bee species at each site with a multifactorial analysis (MFA) on categorized ratios of the load on the load threshold. This analysis enables to discriminate the field sites and countries based on their exposure risk, and to determine the IPAs that are potentially relevant for the exposure risk assessment for each sentinel bee species (Supplementary fig. S2). The exposure risk index ranges from negative to positive values, each side being associated with high and low exposure risks to specific IPAs (Fig. 3). The exposure risk profiles differed among sentinel bees and countries without any clear link with the biogeographic zone, and were primarily driven by the most frequently detected viruses, BQCV, DWV-B, and SBV (Fig. 3). In *A. mellifera*, most sites from Ireland, Spain and Sweden clustered on the negative side of the index, associated with high exposure risks of DWV-A and DWV-B and low risks of BQCV and the two honey bee microsporidia (Fig. 3A; Supplementary table S10). On the opposite, most sites from Estonia, Italy and the United Kingdom clustered on the positive side of the index associated with a high exposure risk of SBV during field deployment and low risks of BQCV, and of DWV-A and B during field exposure (Supplementary table S11). In this sentinel bee species, sites from Switzerland and Germany were equally distributed on both negative and positive sides of the index, indicating no specific risk profiles for these countries (Fig. 3A). In *B. terrestris*, the site and country distributions of the exposure risk values were similar to that in *A. mellifera*, yet associated with different risks (Fig. 3B). Most sites from Ireland, Spain and Sweden, but also those from Switzerland, clustered on the negative side of the index associated with low risks of the three viruses BQCV, DWV-B and SBV (Supplementary table S12). Most sites from Estonia and Italy clustered on the opposite positive side at high risks of BQCV and SBV (Supplementary table S13). German sites were equally distributed on both negative and positive sides of the index, indicating no specific risk profile. In *O. bicornis*, the exposure risk profile relied on the four detected viruses and differs from the two others: more than half the German and Estonian sites clustered on the negative side of the index at low exposure risks of these four viruses, while Swedish sites exhibited the most positive index values associated with high exposure risks (Fig. 3C; Supplementary tables S14, S15). The exposure risk profiles did not differ between focal crops for the three sentinel bees (Supplementary fig. S3).

### Variation in IPA loads among bee species

**Figure 4:**
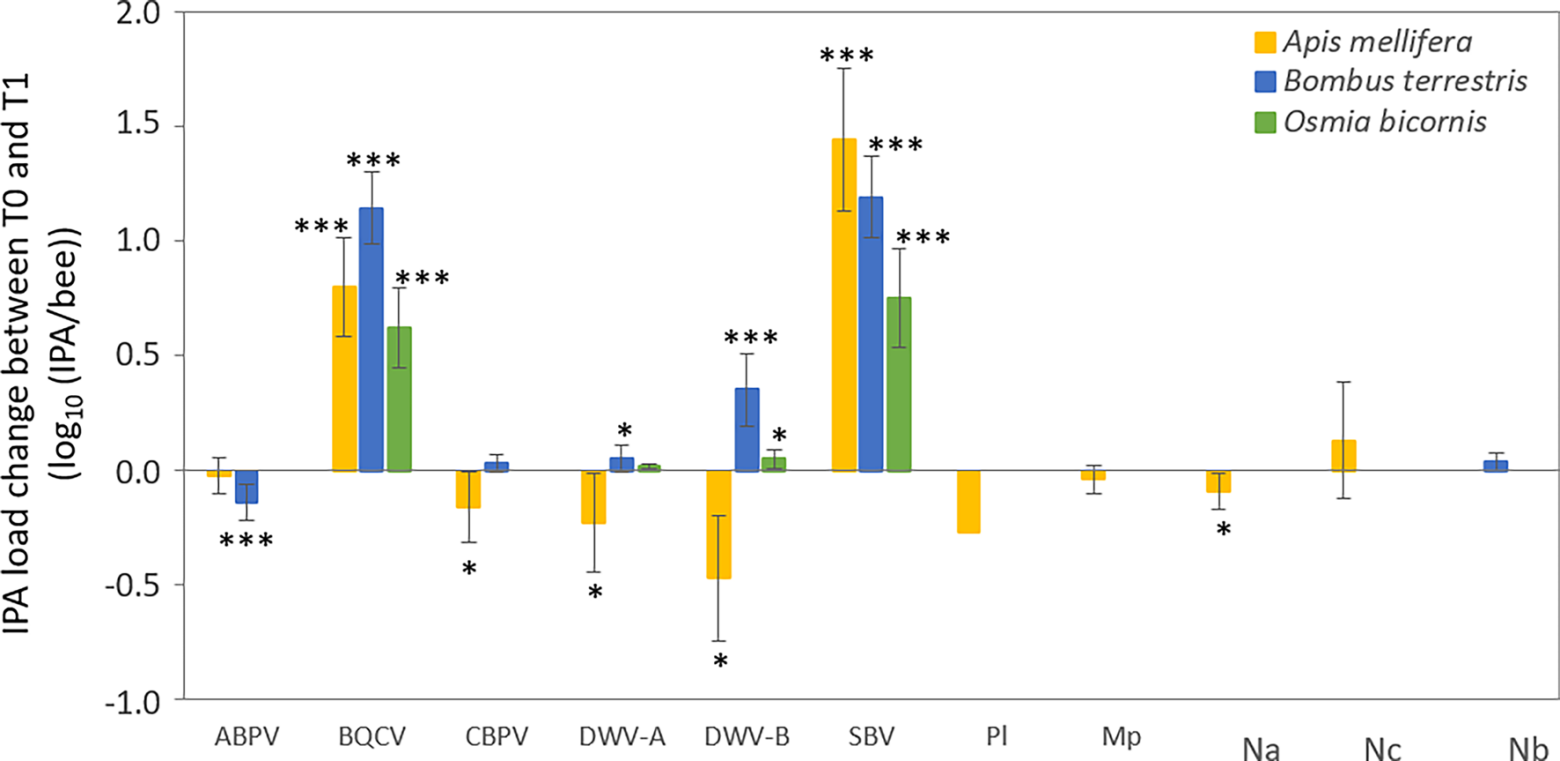
Change in IPA loads between T1 and T0 screening occasions (mean ± 95% CI) for the most frequently detected IPAs in each bee species. For the analysis to be possible, the non-detected analytical results were replaced by the corresponding limit of detection of the molecular method. Significance of one-sample t tests against the mean load change of 0 are indicated as follows: * *P* < 0.05, *** *P* < 0.0001 (similar results were obtained with non-parametric Mann–Whitney). Pl, *Paenibacillus larvae*; Mp, *Melissococcus plutonius*; Na, *Nosema apis*; Nc, *Nosema ceranae*; Nb, *Nosema bombi*.

**Figure 5:**
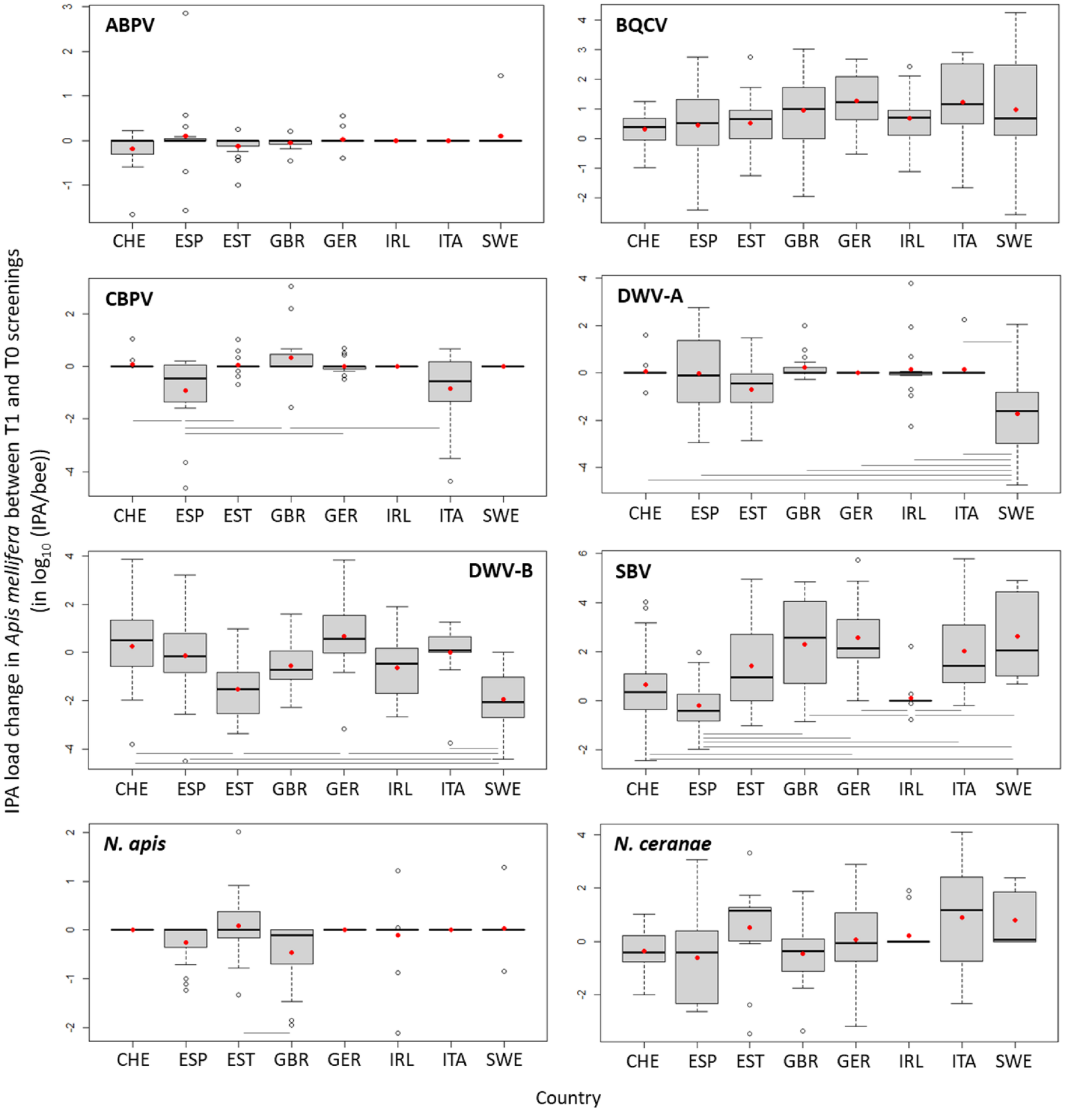
Change in IPA loads between T1 and T0 screening occasions (quartiles, median and mean as red points) for the most frequently detected IPAs quantified in *Apis mellifera* in each country (CHE, Switzerland; ESP, Spain; EST, Estonia; GBR, United Kingdom; GER, Germany; IRL, Ireland; ITA, Italy and SWE, Sweden). For the analysis to be possible, the non-detected analytical results were replaced by the corresponding limit of detection of the molecular method. Significant Tukey pairwise comparisons are indicated with bars.

The analysis of the change in IPA loads between screening T1 (after field exposure) and screening T0 (before field exposure) showed that loads changed differently among the IPAs, depending on the country and the bee species (Fig. 4; Supplementary fig. S4; Supplementary tables S16, S17, S18, S19). For *A. mellifera*, the colonies deployed in the field at T0 already harbored substantial loads of BQCV, both genotypes of DWV (A and B) and SBV (Supplementary fig. S4). The *A. mellifera* IPA loads at this screening occasion varied significantly among countries (Supplementary table S16), although without any identifiable pattern, nor did any one country exhibit consistently higher or lower loads for all of the IPAs (Supplementary fig. S5). During field exposure, the mean load change was null for ABPV and *N. ceranae*, indicating that the loads were similar at both screening occasions, while those of BQCV and SBV increased, and those of CBPV, DWV-A and B and *N. apis* decreased mostly with small amplitudes (Fig. 4; Supplementary fig. S4). The *A. mellifera* BQCV, DWV-A, DWV-B and SBV T0-T1 load changes varied among the eight countries, without any relationship to the initial level of variation at T0. None of the countries exhibited either consistently higher or lower load changes for all the IPAs, *i.e.* the distributions of load changes of each IPA was largely independent of the other IPAs, when analysed by country (Fig. 5; Supplementary fig. S5). There was no significant variation among countries for ABPV and BQCV load changes, and differences among countries were small for microsporidia (the country effect relied on a single significant pairwise difference for *N. apis*; and on a marginal difference between Spain and Italy for *N. ceranae*, *P* = 0.06; Fig. 5). For the bacterial agents of foulbroods, the load of *P. larvae* (AFB) detected at a single site decreased slightly (-0.27 log_10_ (IPA/bee)), while the loads of *M. plutonius* (EFB) did not differ between screening occasions (Fig. 4; mean change ± 95% CI at positive sites: -0.75 ± 0.90 log_10_ (IPA/bee), one-sample t test and Mann–Whitney *P* = 0.15). Note that EFB symptoms were only associated with *M. plutonius* loads above 7.5 log_10_ (IPA/bee) (Supplementary fig. S6).

**Figure 6:**
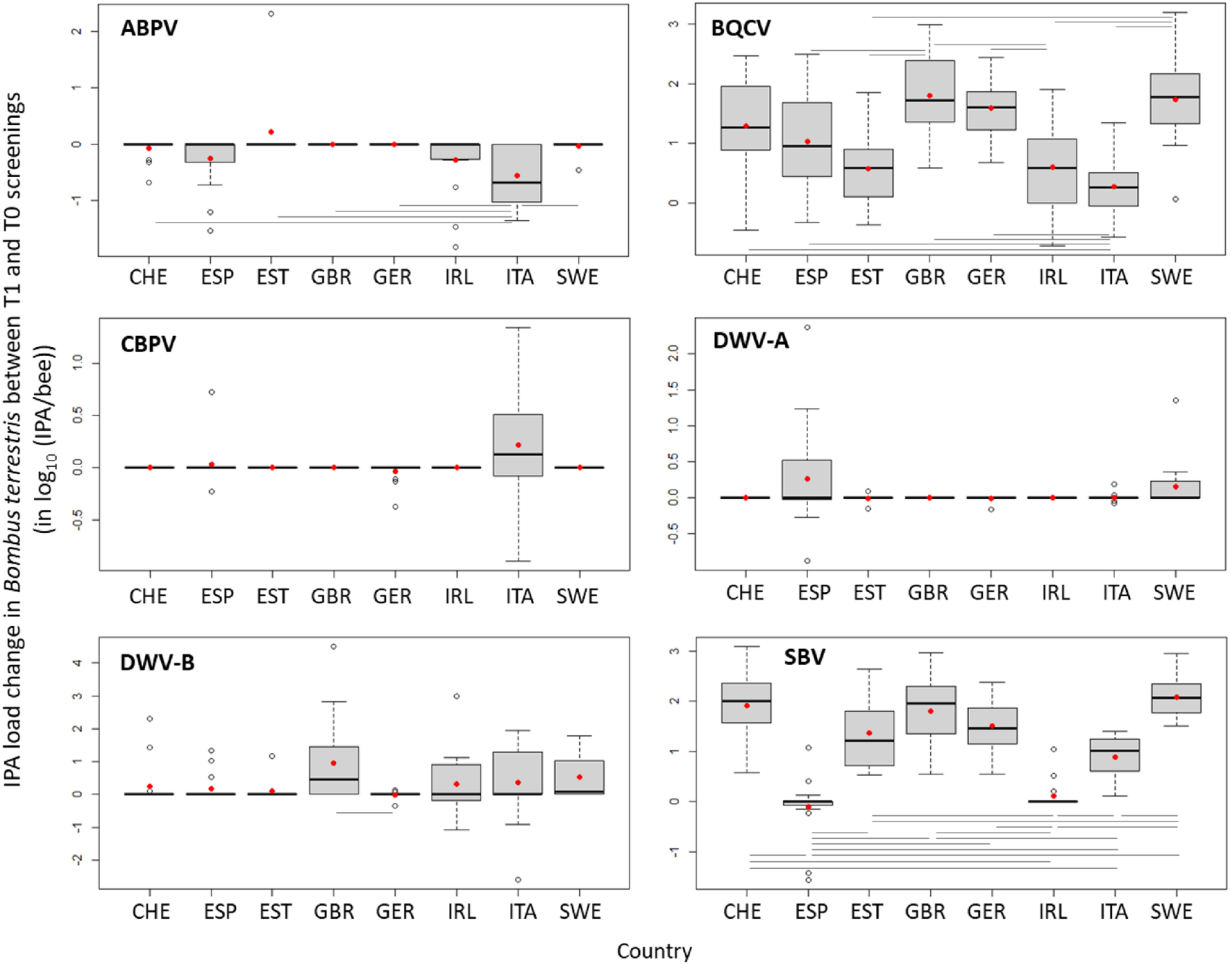
Change in IPA loads between T1 and T0 screening occasions (quartiles, median and mean as red points) for the most frequently detected IPAs quantified in *Bombus terrestris* in each country (CHE, Switzerland; ESP, Spain; EST, Estonia; GBR, United Kingdom; GER, Germany; IRL, Ireland; ITA, Italy; SWE, Sweden). For the analysis to be possible, the non-detected analytical results were replaced by the corresponding limit of detection of the molecular method. Significant Tukey pairwise comparisons are indicated with bars.

In *B. terrestris* colonies, the IPA loads were overall much lower than those of *A. mellifera*, both before and after exposure to field conditions (Fig. 2; Supplementary fig. S4). Despite the uniform and standardized origin of all *B. terrestris* colonies, the loads of five of the six viruses at T0 (*i.e.* prior to deployment in the field) varied significantly among countries (Supplementary fig. S7; Supplementary table S16). The exception was CBPV (Supplementary tables S16, S19), again largely because it was detected only very rarely, and only in a few countries, at both T0 and T1. Upon field exposure, the loads of all viruses except ABPV and CBPV increased with varying amplitudes (Fig. 4). The loads of ABPV decreased over time while those of CBPV remained constant (Fig. 4), although both of these results are based on just a few positive samples in a few countries (Supplementary fig. S8; Supplementary table S20). Also the T0-T1 changes in loads varied significantly between countries with positive sites for four of the viruses (ABPV, BQCV, DWV-B and SBV; Fig. 6). As for *A. mellifera*, this variation was independent of the variation among countries observed at T0, and none of the countries exhibited consistently higher or lower changes in IPA loads across all IPAs (Fig. 6; Supplementary fig. S7). The microsporidium *N. bombi* was only detected at four sites at T1, which naturally is an increase in both prevalence and load on its absence at T0 (Fig. 4). This load increase was not significant when considering those four sites (1.35 ± 0.10 log_10_ (IPA/bee); one-sample Mann–Whitney *P* = 0.10) but not when taken in the context of the general absence of *N. bombi* across all 128 sites (one-sample t-test *P* = 0.05).

**Figure 7:**
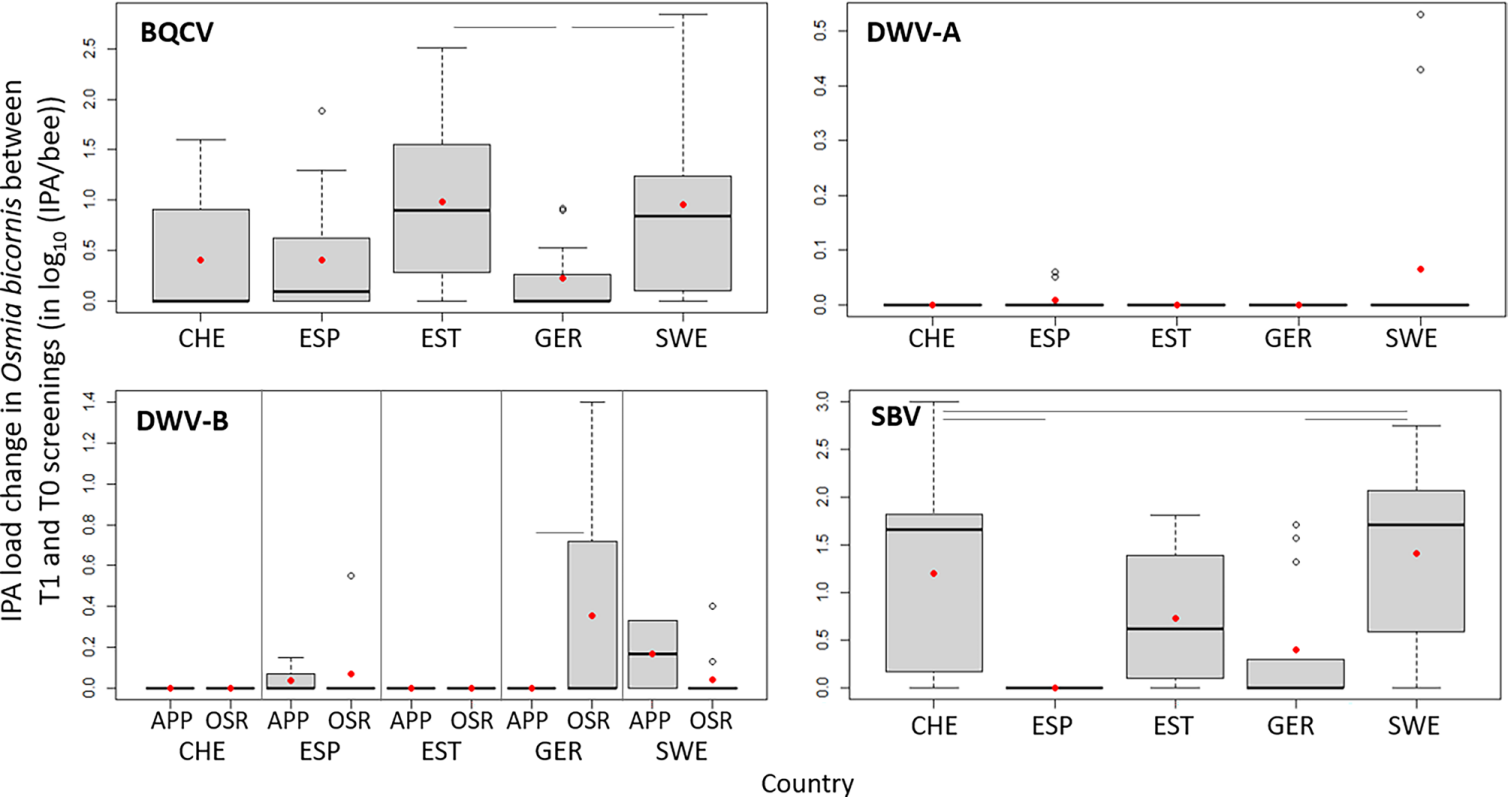
Change in IPA loads between T1 and T0 screening occasions (quartiles, median and mean as red points) for the most frequently detected IPAs quantified in *O. bicornis* in each country (CHE, Switzerland; ESP, Spain; EST, Estonia; GER, Germany and SWE, Sweden). For the analysis to be possible, the non-detected analytical results were replaced by the corresponding limit of detection of the molecular method. Significant Tukey pairwise comparisons are indicated with bars. The load changes are split by focal crop for DWV-B to illustrate the significant effect in the statistical analysis.

The loads of the four viruses detected at T1 in *O. bicornis* (BQCV, A and B genotypes de DWV, and SBV) were overall much lower than those of *A. mellifera* and slightly lower than those of *B. terrestris* (Fig. 2). The load of the single sample positive to *N. ceranae* fell in the middle of the range of *A. mellifera* loads. Since none of the IPAs were detected at T0 (Fig. 1C), the load change of those detected IPAs was inevitably an increase over the non-detection at T0. There were a few differences among countries for BQCV and SBV load changes (Fig. 7).

### Effect of the focal crop and exposure time on IPA load changes

Overall, the focal crop on which bees were deployed did not consistently explain the variation in the changes in IPA loads. The significant focal crop effects, both alone and in interaction with the country, for two IPAs quantified in *B. terrestris* and *O. bicornis* was likely due to the small numbers of data points (ABPV; *B. terrestris*) and differences in load change variability between oilseed rape and apple as the focal crop (DWV-B; *B. terrestris* and *O. bicornis*), the highest variability not being associated with the same crop among countries (Fig. 7; Supplementary figs S7, S8, S9, S10, S11, S12; Supplementary tables S17, S18, S19, S20).

The time of field exposure varied from 14 to 55 days for *A. mellifera*, 14 to 99 days for *B. terrestris*, and 13 to 59 days for *O. bicornis*. This did not consistently explain the variation in the changes in IPA loads despite its wide range of values, although BQCV load changes increased with the increasing *A. mellifera* exposure time (estimate ± se: 0.10 ± 0.02). The other few significant exposure time effects, alone and in interaction with the country likely relied on the few data points and the differences in load change among countries (Supplementary tables S17, S18, S19).

### Loads of different IPAs after field exposure within the three sentinel bee species

The microsporidia *N. apis* or *N. ceranae* and most bee viruses are acquired orally and shed again into the faeces for dispersal into the environment. There is both causal and correlational evidence that microsporidia and certain bee viruses, principally BQCV, benefit mutually from co-infection, leading to correlated titres at laboratory, colony and field-landscape levels^36,73,74^. In this study, no significant correlations were found between microsporidia and virus loads in *A. mellifera* (Supplementary figs. S13, S14, S15; Supplementary tables S21, S22).

As can be expected for different strains of the same virus, the loads of the A and B genotypes of DWV were positively correlated in both *A. mellifera* and *B. terrestris*, though not in *O. bicornis* (Supplementary table S23; Supplmentary fig. S16). In *A. mellifera*, both genotypes were detected mostly at sites where the honey bee-specific, obligate ectoparasitic mite *Varroa destructor*, which vectors DWV-A passively and DWV-B circulatively^34,75^, was present in the colonies (87% of samples positive to DWV-A and 79% of those positive to DWV-B; Supplementary fig. S17). The loads of both DWV genotypes were also positively correlated to the mean mite loads (Supplementary table S24), possibly contributing indirectly to the positive relationship between the DWV-A and DWV-B loads (Supplementary fig. S17). The positive correlations between DWV-A and B in *A. mellifera* and *B. terrestris* were not linked to the observation of bee deformities (Supplementary figs. S16, S17), which only tend to become apparent at very high mite and DWV loads towards the autumn season^76^.

### Potential for transmission of IPAs between sentinel bees

**Figure 8:**
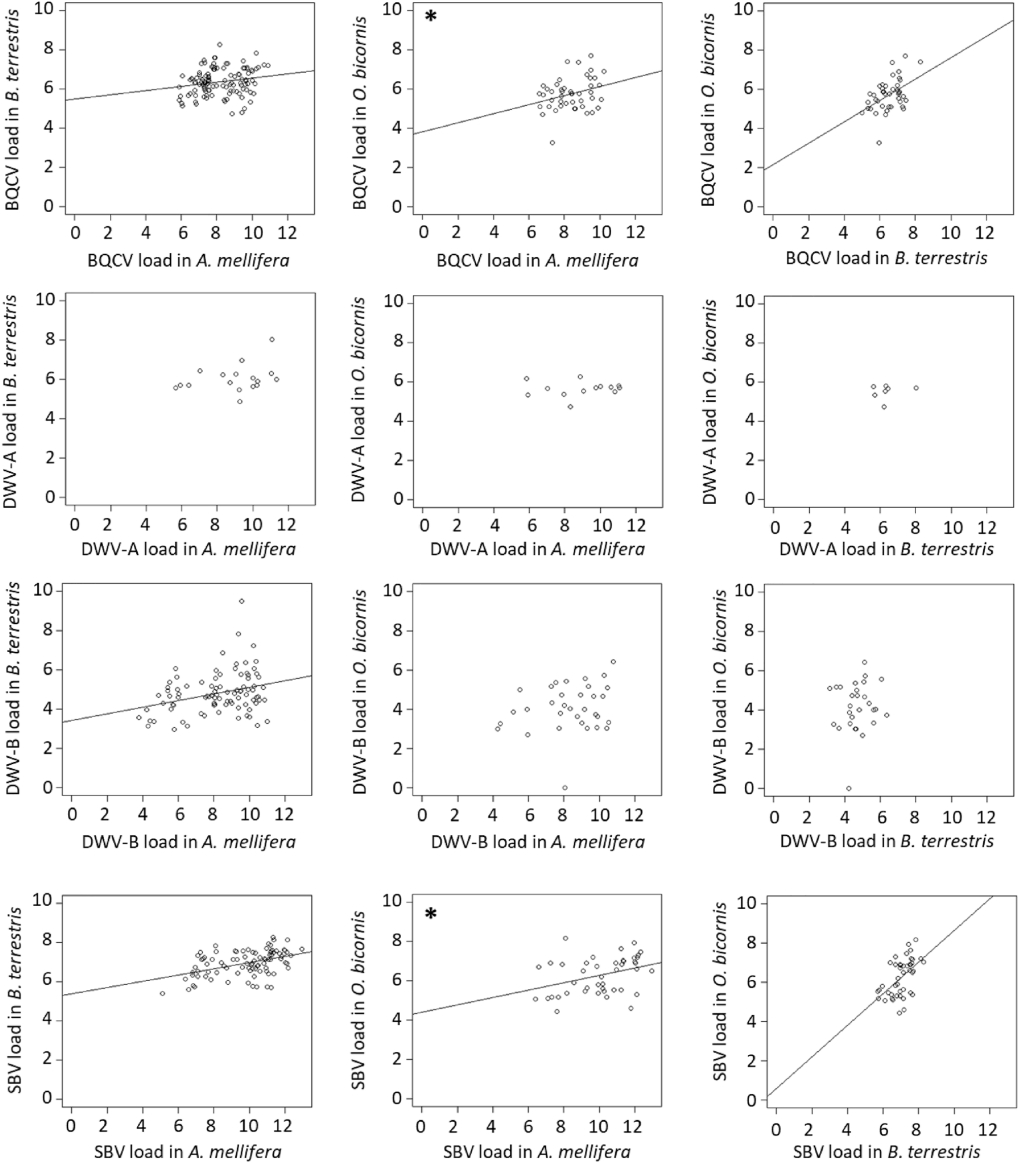
Relationship between the loads of BQCV, DWV-A, DWV-B and SBV (in log_10_ (IPA/bee)) after field exposure (T1) in the different bee species at the same sampling sites. Regression lines are shown when the correlation was statistically significant (Pearson’s correlation, *P* < 0.05). Asterisks indicate positive correlations that are not significant when these loads are corrected for differences among countries (Supplementary table S26).

The potential for IPA transmission among bee species was explored by testing for positive correlations between the loads and between the loads and load changes for IPAs that were co-detected at the same sites in multiple sentinel species before bee deployment and after the exposure to field conditions and the bees’ close coexistence for a few weeks. Only the viruses DWV-A, DWV-B, BQCV, SBV and CBPV were detected in multiple bee species at the same sites. The remaining IPAs were either only detected in a single bee species (the two bacteria and three microsporidia species) or in different species but at different sites (ABPV). The loads of DWV-A were not correlated for any species pairs (Fig. 8; Supplementary table S25). The loads of DWV-B were positively correlated only between *A. mellifera* and *B. terrestris*. This relationship remained when correcting for country-specific loads (Supplementary table S26). The loads of BQCV and SBV were positively correlated for all the species pairs, with strongest correlations between *B. terrestris*–*O. bicornis* than *A. mellifera* (Fig. 8;Supplementary table S25). However, the significant positive correlations between *A. mellifera* and *O. bicornis* for these two viruses became smaller and non-significant after correcting for the differences in loads among countries (Supplementary table S26). CBPV was detected in both *A. mellifera* and *B. terrestris* at very few sites, and the loads were not correlated (Supplementary table S25). These positive correlations were not detected between *A. mellifera* and *B. terrestris* prior to their field placement (screening T0, none of the IPAs were detected in *O. bicornis* at this screening) except for a positive and moderate correlation of BQCV loads of similar amplitude as at screening T1 (Supplementary table S25). This suggests that these correlations most likely arose from the field co-location of sentinel bees. In addition, the loads at screening T0 in *A. mellifera* or *B. terrestris* were sometimes correlated positively to the load change in the other bee species between the two screening occasions (Supplementary table S25, Supplementary table S26), meaning that the honey bee and bumble bee loads at field placement were sometimes related to how the loads evolved in the other bee species.

## Discussion

We described the detection frequencies and loads of 11 infectious and parasitic agents (IPAs) in the three managed bee species deployed as sentinels, *A. mellifera*, *B. terrestris* and *O. bicornis*, before and after their field placement in a network of 128 sites. Our study provides the first European-wide assessment of the distribution of 11 common IPAs and of the IPA profile among three closely existing sentinel bees under real-world field conditions. The positive correlations of loads of some of the viruses among co-located bee species provide additional evidence of the potential for IPA transmission between bee species, and the possible threat of high concentrations of managed bee species to sympatric wild bee species. For the three bee species, the changes upon field exposure in IPA richness and diversity, detection frequencies and loads, and the exposure risk profiles, varied among countries without any clear and consistent pattern and were not clearly influenced by the focal crop (oilseed rape or apple). Except for the increasing BQCV loads with the exposure time of *A. mellifera*, the change in IPA loads was also not influenced in any clear pattern by the time of exposure. This indicates that under the specific field conditions of our site network, the bees’ IPA profile was not primarily driven by the site conditions (focal crop, country linked to the biogeographical zone, exposure time). This contrasts with reports of the effects of regional and seasonal variability in pollen diversity and climate conditions on IPA prevalence and loads^38,45,48^. However, it is consistent with the results of a comparable large-scale field study exposing honey bees and bumble bees to the neonicotinoid pesticide, clothianidin, in southern Sweden^12,13^. Yet, the three bee species clearly differed in their IPA profiles (richness, diversity, exposure risk, prevalence, and load).

The ten IPAs infectious to honey bees (excluding the bumble bee parasite *N. bombi*) were all detected in *A. mellifera*. Except for the Irish sites exhibiting the lowest IPA richness, the IPA profile of *A. mellifera* consisted mainly on the six RNA viruses and the two honey bee microsporidia screened, consistently with the high incidences of most of these viruses and *N. ceranae* in spring and summer^34,77–79^, with variability among countries for *N. cereanae*^80^. The brood viruses BQCV and SBV, and the genotype B of DWV were present at the highest detection frequencies and loads among the six screened viruses. With sporadic detection within each positive country (maximum 38% of sampled sites), ABPV was the least prevalent virus across countries, in line with its documented sporadic presence in *A. mellifera* colonies^81^ and the marked seasonal variability of its prevalence and load^12,78^. Despite variation among countries likely due to the local origin of *A. mellifera*, the IPA richness and diversity, and the detection frequencies of most IPAs remained overall constant during the bloom of the two focal crops, oilseed rape or apple. This is similar to the results of the previously mentioned Swedish field study^12^. By contrast, the loads quantified in *A. mellifera* changed differently during field exposure, depending on the IPA. Also consistently with the Swedish field study^12^, the loads of ABPV, *M. plutonius* and *N. ceranae* remained constant during field exposure, while the loads of CBPV, the genotypes A and B of DWV, and *N. apis* decreased and those of the brood viruses BQCV and SBV increased. In the case of CBPV, BQCV and SBV, our results reflect the seasonal dynamics of these infections: CBPV infections occur mainly in spring and early summer^78^, and the intensity of BQCV and SBV infections increases in summer^78,79^. The increasing loads of BQCV and SBV could also reveal the increased honey bee densities^52^ or field variability in the quality of available food resources (pollen diversity)^38,41^. Yet the stable and decreasing loads of most IPAs may reveal, on the opposite, an overall similar or alleviated level of nutritional stress associated with a higher pollen diversity in the field^82,83^. Note that due to the calculation of the load changes using the detection limit of the method for undetected analytical results, the real load change upon field exposure may be underestimated and the average changes may be greater. The absence of clear relationship between the IPA richness, diversity, detection frequencies, loads in *A. mellifera*, and the focal crop and time of field exposure indicate that in our study, the honey bees’ IPA profile was primarily determined prior to the bees’ deployment. The variation in the IPA profiles across the sites may result from local differences in beekeeping practices as well as the use of different *A. mellifera* subspecies^84^.

The bacterial agents causing the honey bee AFB and EFB, *P. larvae* and *M. plutonius* respectively, were rarely detected and in *A. mellifera* only. This is consistent with the sampling of adult bees^71^, the deployment of honey bee colonies devoid of disease symptoms and the strict colony surveillance for AFB in the European regulation framework (EU Animal Health Law 2016/429). Similarly, the selection of colonies devoid of *N. apis* typical dysentery^85^ may partly explain its low prevalence across countries, except the United Kingdom and Estonia. The lower and decreasing prevalence of *N. apis* in *A. mellifera* and its decreasing loads upon field exposure, compared to *N. ceranae* whose prevalence and loads remained constant, may also partly result from the differences in these microsporidia ecology and infection dynamics (survival, multiplication, infectivity under cold and warm temperatures): *N. apis* infections are more frequent in Northern climates, increase during winter and drop during summer in temperate climates, while *N. ceranae* spores are not resistant to freezing and multiply under summer desiccation and temperatures^86–88^. Coinfections by *N. apis* and *N. ceranae* occurred at a low rate (16.7%) mainly in the United Kingdom and Estonia, and falls within the wide range of reported honey bee coinfections in Europe (< 10%)^86^, Spain (4.4 to 6.5% over 2 years)^43^, Sweden (up to 71%)^88^, and Turkey (18.6 to 49% from a 7-year survey)^89^.

Viral loads can be used to define a transition from covert viral infections (at low loads, less likely to be associated with disease symptoms) to overt viral infections (high loads, more likely to be associated with disease symptoms). For the viruses detected in *A. mellifera* in this study, these load thresholds were similar to the thresholds established previously, based on diagnostic data from French honey bee colonies^72^. The shape of the frequency distributions of ABPV, BQCV and CBPV loads differed from those reported in the French study, but with similar thresholds^72^. This is consistent with the fact that infections do not systematically occur at high loads for a given IPA^12^. The different shape of the ABPV frequency distribution supports a season effect since loads as high as 11 log_10_ copies/bee can be recorded later in summer^78^. However, the bimodal distribution of BQCV loads compared to the previously reported unimodal distribution suggests a higher tolerance to this virus in the deployed *A. mellifera* colonies (in 17% sites exhibiting loads above the threshold). The threshold similarities between two distinct studies indicate that, despite natural variability and seasonality of IPA detection frequencies and loads, some characteristics of the interactions of these viruses with *A. mellifera* hosts are stable across field conditions. For microsporidia, *N. apis* and *N. ceranae* thresholds were similar to each other, and above the empirical threshold of 6 log_10_ spores/bee calculated on data from *N. ceranae* microscopy diagnosis^77^, possibly because of the higher sensitivity of the molecular methods. Based on these thresholds and the IPA loads, the synthetic index of IPA exposure risk showed differences among bee species in their respective risk profiles. Yet the exposure risk relied primarily on the most frequent viruses, BQCV, DWV-A, DWV-B, and SBV. The synthetic index enabled to identify differences in the main exposure risks among countries, mainly for *A. mellifera* and *B. terrestris*, yet without any clear link with the biogeographical zone. Since, due to their common occurrence, these four viruses contribute most to the synthetic exposure index. Subclinical (covert) infections of these IPAs in adult honey bees could be relevant for assessing the exposure risk of bee species where these viruses are commonly detected, allowing discrimination between sites and countries in a sampling network.

The IPA richness, diversity, detection frequencies and loads were generally lower in the two other sentinel bee species, *B. terrestris* and *O. bicornis*. The IPA richness consisted mainly of the most prevalent viruses found also in the sympatric sentinel *A. mellifera*, similarly to a previous report^55^. Although several viruses cross-infect non-*Apis* bees^59,60^, ten out of the 11 screened IPAs are known historically to infect managed *A. mellifera*, hence are likely more adapted to this host. The differences between bee species could partly result from the more recent management history of *B. terrestris* and *O. bicornis*, used for crop pollination in greenhouses, open fields, and orchards^90–92^. It could also result from the differences on several aspects of their life histories, ecology and management, which could affect their level of exposure to stressors: sociality level (colonies of thousands of *A. mellifera* and of hundreds of *B. terrestris*, solitary nesting *O. bicornis*), life cycle (multiple worker generations a year for *A. mellifera* and *B. terrestris vs.* monovoltine *O. bicornis*), foraging period (longer periods for *A. mellifera* and *B. terrestris vs.* a few weeks for mason bees), foraging distance (up to a few kilometres for *A. mellifera* and *B. terrestris vs.* within 1 km for *O. bicornis*) and management history (millennia for *A. mellifera*, a century for *B. terrestris*, and a few decades for *O. bicornis*)^32,90–92^. This could also be linked to the uniform commercial origins of the colonies and cocoons of these two sentinel bee species, while the *A. mellifera* hives were obtained from local beekeepers and therefore had been placed under other field conditions prior to this study, with the potential presence of several but different IPAs at different loads. The *B. terrestris* IPA set consisted of the six screened viruses both before and after the bee’s field placement, and with increasing frequencies upon field exposure. The viruses screened in this study have already been reported to infect this bumble bee species^35,93,94^, and some of them were previously detected in commercial *B. terrestris* colonies^95–97^. As for *A. mellifera*, BQCV, SBV, and DWV-B were the most prevalent viruses in bumble bees, with loads similar to those previously reported for the virus DWV-A^97﻿^, but lower to those quantified in *A. mellifera*. The microsporidium *N. bombi*, described historically in *B. terrestris*^98^, was detected at a few sites in this bee species only. Given that the same supplier provided the *B. terrestris* colonies for the entire site network through local suppliers, and the parasite was not detected before field placement, the bumble bees likely acquired *N. bombi* from the field. In a similar manner, none of the screened IPAs were detected in the solitary bee *O. bicornis*, whose cocoons were provided by a single supplier for the entire site network, indicating either their absence or their undetectable presence using our molecular methods. The IPAs detected in this species after field placement were likely acquired from the field, and included four viruses (BQCV, SBV, and DWV genotypes), and a single detection of *N. ceranae*. Those viruses were previously reported to infect non-*Apis* wild bees^67,99–101^, including *Osmia* spp. and specifically *O. bicornis*^99,101–103^. As for *B. terrestris*, loads of these viruses were lower than those in *A. mellifera*. This mirrors the lower virus loads reported in several solitary bee species, compared to those of sympatric *A. mellifera* apiaries^100^. For both *B. terrestris* and *O. bicornis*, detection frequencies and loads increased after field placement (except for ABPV in *B. terrestris*), regardless of the exposure time at the field sites. This suggests possible lower resistance or higher tolerance levels to these IPAs which are mainly honey bees’. Alternatively, as discussed earlier for *A. mellifera*, this could also result from variability in food resource availability^38,41^ or increased colony densities in the case of *B. terrestris*^52^.

In our analysis of interspecies correlations, the loads of BQCV and SBV were positively correlated for the species pairs except for *A. mellifera*-*O. bicornis*, and the loads of DWV-B were positively correlated only between *B. terrestris* and *O. bicornis*. Except for the BQCV correlation between *A. mellifera* and *B. terrestris* at screening T0, no bias came from a pre-existing correlation between the loads before the placement of bees in the sites. The increasing detection frequencies and loads of these viral IPAs in *B. terrestris* and *O. bicornis* upon field exposure, the overall lower loads in these two sentinel bees than in *A. mellifera*, together with the positive correlations of BQCV, DWV-B and SBV loads quantified in the different bee species from the same sampling sites, suggest that these IPAs could have been transmitted among bee species. In PoshBee’s site network, the three bee species were deployed at appropriate distances from each other at the crop edge^32^. Given that foraging distances of *A. mellifera* and *B. terrestris* cover that of *O. bicornis*^32^, the three bee species shared partially the same floral resources. The potential IPA transmission among managed honey bees and bumble bees, and wild bumble bees, solitary bees, and other hymenoptera and arthropods, is well documented^60–63,66,99,104–112^. Recently, a pan-European field survey of 12 sampling sites reported that prevalence of three viruses including DWV, in wild bumble bees and solitary bees was determined by the virus presence in sympatric managed honey bees and by abiotic environmental conditions (temperature, vegetation flowering period)^48﻿^. Interspecies IPA transmission can occur through sharing food resources^8,112–114^, yet this might not be systematic^61^. Although our correlative results do not enable to conclude on the directionality of this potential IPA transmission, the colonies of *A. mellifera* contain larger numbers of foragers than *B. terrestris* colonies and *O. bicornis* trap nests, which likely makes honey bees the most frequent sentinel bees at each site. Combined with the positive correlations between the *A. mellifera* loads at screening T0 and the load changes upon field exposure in the two other sentinel bees, this would point to a likely transmission of viruses from managed *A. mellifera* to the two other sentinels, yet without excluding a possible additional transmission from *B. terrestris* to *O. bicornis*. The absence in *B. terrestris* and *O. bicornis* of the bacterial agents of honey bee foulbroods and *N. apis* infectious to honey bees, the rare presence of *N. ceranae* (in a single *O. bicornis* sample), as well as the absence of the bumble bee parasite *N. bombi* in honey bees and mason bees, would also point to the virus transmission from deployed *A. mellifera*, possibly via bees’ faeces on flowers^60–62^. The lower viral loads and load thresholds in *B. terrestris* and *O. bicornis* compared to *A. mellifera* and the similar frequency distributions of BQCV and DWV-A loads in *B. terrestris* are consistent with previous reports^60,115^. Lower infection intensities might be due to a combination of host specificity of the screened viruses, different management practises of the bee species before field exposure, the more recent management histories for pollination of these two bee species, and different colony sizes and social organisations^31,90–92,107^. Differences in pollination behaviour could also contribute to the IPA profile of *O. bicornis* (low richness, diversity, detection frequencies and loads) in situations when *Osmia* spp. did not share the same flowers as *A. mellifera* and *B. terrestris*. Indeed, mason bees are used as efficient fruit tree pollinators^92^, but they also collect oilseed rape pollen when present in their environment^11,116^. Yet, there was no clear differences in *O. bicornis* IPA detection frequencies and loads between the two focal crops in our field study. Additional analyses that include data related to the pollen available to the bees and the pollinator abundance would help explore deeper the contribution of environmental conditions to the bee’s IPA profile.

After bees were placed on the site for several weeks, we found no significant correlations between the loads of either BQCV, DWV-A and B, and the loads of the microsporidia *N. apis* and *N. ceranae* in *A. mellifera*, in contrast with the previous reports of positive relationship between BQCV and *N. apis*^36,73^, and a negative relationship between DWV and *N. ceranae* in adult honey bees^74^. For the virus DWV, the loads of the two genotypes A and B were positively correlated in *A. mellifera*, as previously documented^117,118^, without any clear link with the presence of bee deformities. This positive correlation may be explained by the positive correlations of the loads of either viral genotype with the loads of *V. destructor* mite which vectors these viruses and contributes to the epidemiology of the deformed wing disease^75,118^. While *V. destructor* does not colonize the bumble bee colonies, a similar positive correlation appeared for *B. terrestris* without a link with the presence of bee deformities, and no correlation was found for *O. bicornis*. No consensus exists on the concomitant observations of bee deformities and DWV infections in *A. mellifera* or *B. terrestris*^113,119^. The *V. destructor* mite is a frequent transmission route of DWV genotypes, almost exclusively associated with high viral loads^73,120^; yet these viruses can be transmitted vertically (via eggs and sperm) and horizontally via resource sharing and hygienic behaviour, leading to covert infections at lower viral loads without wing deformities^﻿55,59,120^, as observed in the *B. terrestris* samples in our study.

To summarize, the detection and quantification of 11 common bee IPAs in the field network of 128 sites across eight European countries revealed differences among the three sentinel bee species, *A. mellifera*, *B. terrestris*, and *O. bicornis*. The specificities of their IPA profiles (richness, diversity, detection frequency, load, and change over time, load threshold and exposure risk) upon field placement and exposure to realistic environmental conditions in the field was not mainly driven by the flowering crop system, nor the country where bees were deployed. Further exploration of the contributions of multiple landscape parameters at each site would improve the understanding of environmental effects in the bees’ IPA profile. In our study, the bees’ management history and biology, their coevolution history with the ten honey bee IPAs screened, as well as the IPA transmission between bee pollinators via floral resource sharing, likely played crucial roles. Further analyses, for instance using sequencing of viruses, would provide additional data on the potential IPA transmission and its directionality. Our descriptive study confirms the complexity and variability in the assessment of the stress sources affecting bee health. It further indicates that the most frequently occurring IPAs in adult bees of the three sentinel bee species (BQCV, DWV-B and SBV) could be reliable variables to analyse the bee risk of IPA exposure and reliable indicators of IPA transmission among managed bees and possibly among bee pollinators (wild and managed) in general. Our study provides an additional building block in the wide-scale assessment of the IPA exposure of bee pollinators and in the understanding of IPA transmission among them. Our results shed light on the possible consequences of honey bee management strategies in ecologically sensible environments such as biodiversity conservation areas. Our study also revealed background inter-survey stability in *A. mellifera* interactions with its common IPAs, indicating that these IPAs may be of interest for epidemiology longitudinal and cross-sectional surveys.

## Material and methods

### Screened pathogens and parasites

The 11 IPAs screened in the three bee species included six RNA viruses (ABPV, BQCV, CBPV, DWV-A, DWV-B, and SBV), the two Gram-positive bacteria *P. larvae* and *M. plutonius* (responsible for the notifiable honey bee diseases AFB and EFB, respectively), and the three intracellular microsporidian parasites (*N. apis* and *N. ceranae* historically described in honey bees, and *N. bombi* historically described in bumble bees).

### Design of the experimental landscape study site system

Three colonies each of three sentinel bee species: honey bees (*A. mellifera*)*,* bumble bees (*B. terrestris*)*,* and mason bees (*O. bicornis*) were deployed between March and July 2019 at each of 128 field sites in eight European countries (Estonia, Germany, Ireland, Italy, United Kingdom, Spain, Sweden, Switzerland), covering four biogeographical areas (Atlantic, Boreal, Continental, Mediterranean)^32^. In each country, 16 sampling sites were selected around two types of bee-pollinated mass-flowering crops of economic importance, distributed throughout Europe and with different crop management systems: eight landscapes centred around oilseed rape field (OSR), an annual arable crop, and eight landscapes centred around apple cultivation (APP), a perennial orchard crop^32^ (Supplementary fig. S18). In each country and for each crop, the landscapes were selected along a gradient of land-use intensity within a 1 km radius of the focal crop field site, using the proportion of arable cropland (OSR) and orchards (APP) as a proxy for exposure to agrochemicals and other land use intensity-related environmental stressors^32^.

### Sentinel bee colony provenance and deployment

The three *A. mellifera* colonies at each field site were sourced locally in each country, ideally from the same beekeeper for both focal crops although different countries had slightly different sourcing strategies: some countries (*e.g.* Sweden) sourced all honey bee colonies from a single supplier and distributed these to the various Swedish sites; other countries (*e.g.* Ireland, United Kingdom) sourced colonies from different local beekeepers near each site, through the local beekeeping organizations; other countries (*e.g.* Italy) sourced colonies from a single beekeeper for each focal crop. The deployed colonies belonged to five *A. mellifera* subspecies and subspecies mixes (Supplementary table S27). All honey bee colonies were certified free from the notifiable diseases AFB and EFB prior to deployment, and with acceptably low levels of other IPAs and pests. During field exposure of the bees, the colonies were tested for the presence of *Varroa destructor* mites, visual symptoms of AFB and EFB in honey bee hives, and the occurrence of wing deformities in honey bees and bumble bees were recorded^32^. The three *B. terrestris* colonies (‘Standard Hive’) at each site were obtained from the Biobest Group NV (Westerlo, Belgium) through local retailers (the local subspecies *B.t. audax* was used in the United Kingdom and Ireland while the subspecies *B.t. terrestris* was used on mainland Europe). Only for the six mainland countries in the site network, the three *O. bicornis* trap nests at each field-site were produced by The Red Bee Hive Company Ltd (Southampton, England) and seeded with a standard number of male and female cocoons sourced from Wildbiene & Partner AG (Zürich, Switzerland)^32^. All sentinel bee colonies and species were deployed before the blooming of the local focal crop at each site, each species being placed at appropriate distances from each other^32^.

### Sample collection strategy and method

Samples of the three bee species were collected at two time-points: immediately prior (screening T0), and during or after (screening T1) the main flowering period of the local focal crop at each site. The chronological timing of sample collection varied considerably across the 128 sites in the 8 countries, depending on logistics and crop phenology (March–May for T0 and May–July for T1)^32^.

For each bee species at each site and each sampling occasion, a single sample of pooled bees was collected across the three colonies/trap nests as a solution for statistical analyses to the artifice of the *Osmia* trap nest replication, the considerable degree of drift and mixing between honey bee colonies, and the surplus of higher level replication and semi-replication throughout the experimental design^32^. The three colonies/trap nests for each bee species were, as much as possible, equally represented in their respective pooled sample by pooling the bees on-site. For the *A. mellifera* samples, a pooled sample of 60 worker bees (20 per hive) were collected at both T0 and T1 (Supplementary fig. S19) from the outer frames of the upper hive chambers^32^, which is where the age distribution of the bees is most representative of the entire colony^121^. This sample will therefore include both young nurse bees in contact with brood specific IPAs (*P. larvae* and *M. plutonius*) and older forager bees exposed to adult IPAs (CBPV, *N. apis* and *N. ceranae*) and the external environment. Some of the IPAs infect both brood and adult honey bees (ABPV, BQCV, DWV, SBV)^122,123^ with the primary pathology usually in either brood (BQCV, SBV) or adult bees (ABPV, DWV). For the *B. terrestris* samples, equal numbers of bumble bee worker bees were collected from inside each nest after closing the colony entrance. The T0 sample contained 12 bees (4 bees per colony, so as not to impact colony performance) while the T1 sample contained 30 bees collected after nests were collected and euthanized^32^ (10 bees per colony; Supplementary fig. S19). For the *O. bicornis* samples, at T0 a single pool of 10 females was provided by each of the six mainland countries involved, straight from the country’s total *O. bicornis* allocation and prior to field deployment of the cocoons. At T1, female *O. bicornis* bees were sampled at each site when returning to the nests^32^, although the number of *O. bicornis* sampled varied widely between sites, from 1 to 43 bees per sample (Supplementary table S28). Because *O. bicornis* bees did not install in the trap nests deployed in Italy, the pre-screening sample was not included in the analysis.

### Sample management, transport and initial processing

The pooled bee samples were transported on ice from the field sites and stored at − 20 °C at a local storage facility within 12 h to minimize degradation of the nucleic acids. The samples from each country were subsequently sent on dry ice (− 40 °C) to ANSES, where they were stored at − 80 °C until processing, without breaking the cold transport chain. A crude cleared homogenate was prepared from each sample by grinding the bees in sterile 10 mM phosphate buffer (pH 7.0; 1 ml buffer per honey bee and solitary bee, 2 ml buffer per bumble bee) using tubes with a rotor–stator element (DT-50 or DT-20) on an Ultra Turrax® tube drive (IKA). To disrupt spores and bacterial cocci, the homogenates were then finely ground in 2-ml microtubes containing 750 mg of 0.1–0.25 mm glass beads and five 3 mm inox beads on a Mixer Mill MM400 (2 cycles, 30 s at 3000 Hz; Retsch). Homogenates were centrifuged twice (8000 × *g*, 10 min, 4 °C) to sediment the glass and metal beads, and remaining cuticle debris. The cleared homogenates were stored at − 80 °C until further processing.

### Nucleic acid extraction and purification

Viral RNA, and bacterial and microsporidian DNA were collected and purified simultaneously from 150 µl of cleared homogenate using the NucleoSpin® 8 Virus—Viral RNA and DNA isolation kit, according to the manufacturer’s instructions (Macherey–Nagel). Briefly, 48 samples at a time (including bee homogenates, negative grinding controls, and negative and positive purification controls) were lysed in guanidium buffer and proteinase K (56 °C, 10 min) before being transferred onto a vacuum Evo75 automated pipetting workstation (TECAN, Switzerland), where the nucleic acids in each sample were collected on a silica membrane column and purified with a series of washes. The purified RNA and DNA were finally eluted from the membrane with 100 µl of 5 mM Tris–HCl buffer (pH 8.5). The eluates were stored at − 80 °C until further processing. Nucleic acid concentrations measured during validation of the extraction method varied from 7 to 342 ng/µl for natural bee samples, and from 75 to 88 ng/µl for a honey bee matrix enriched with standardized amounts of a plasmid (accuracy profiles and systematic method bias for each IPA quantified in each bee species in Supplementary fig. S20).

### Reverse transcription

For the detection and quantification of the RNA viruses in each sample, 18.8 µl of the total nucleic acid eluate was reverse-transcribed into complementary DNA (cDNA) in a 30 µl reaction at 42 °C for 1 h, in reverse transcriptase buffer (50 mM Tris–HCl, 75 mM KCl, 3 mM MgCl_2_, pH 8.3), with 30 pmol of random hexamer primers (Invitrogen), 0.75 mM dNTP mix (Invitrogen), 30 U RNase OUT (Invitrogen), and 300 U of Superscript II reverse transcriptase (Invitrogen).

### Harmonised high-throughput real-time quantitative PCR

IPA detection and quantification were performed on pure cDNA for viruses and on two-fold diluted DNA samples in elution buffer for bacteria and microsporidia, by probe-based real-time qPCR with 1X LightCycler® Real-time Ready (Roche) on a LightCycler® 1536 real-time PCR thermal cycler (Roche, Meylan, France). qPCR mixes were previously optimised and harmonised to reach 500 nM of each primer and 200 nM of probe in 2-µl reactions (except for DWV-B, see below; details of primers and probes in Supplementary table S29). A Bravo automated liquid handling platform equipped with a chiller and a PlateLoc thermal microplate sealer (Agilent Technologies, La Jolla, CA, USA) was used as a microplate dispenser to load 1 µl mix and 1 µl sample on 1536-well plate. Quantitative PCR thermal conditions were 95 °C for 3 min followed by 45 cycles at 95 °C for 10 s and 60 °C for 30 s. PCR inhibition was checked in each DNA and cDNA sample by assessing the extinction of the exogenous amplification control (*Ehrlichia canis* gene sequence) added to the PCR reaction^124,125^.

ABPV and DWV-B cDNAs were detected and quantified on a QuantStudio® 5 real-time PCR device (Applied Biosystems) in 20-µl reactions, with 5 µl cDNA sample, 1X LightCycler® 480 Probes Master, and 50 nM of the passive reference dye ROX. For DWV-B quantification, the PCR mix contained 1200 nM of each primer and 400 nM of probe; for ABPV quantification, we used the same PCR mix as described above. Thermal conditions on this PCR device were 95 °C for 3 min followed by 45 PCR cycles at 95 °C for 10 s, 60 °C for 30 s, 72 °C for 25 s, and a final cooling at 40 °C for 10 s. The measurement uncertainty was calculated for each IPA quantification method using the IPA loads of the positive controls (Supplementary fig. S21).

### Data analysis

Using qualitatively the analytical results, the IPA richness at each site was calculated as the number of detected IPAs and the detection frequencies were calculated as the proportion of positive samples among the samples analysed for one sentinel bee species, and for focal crop or country for each bee species. Quantitative results were expressed as the decimal logarithm of IPA copy numbers per bee (log_10_ (IPA/bee)), as extrapolated from the qPCR results and the primary homogenate volumes and dilutions^122^. For each sentinel bee species, the changes between screening occasions T0 and T1 in IPA richness (discrete count data) were analysed with one-sample Mann–Whitney’s tests against the mean = 0. The changes in IPA diversity (quantitative data) between screening occasions and among countries was tested with one-sample t tests. For each IPA in each sentinel bee species, changes in detection frequencies between screenings occasions and between focal crops were tested with χ^2^, with Fisher’s correction when needed.

For each frequently detected IPA (viruses and honey bee microsporidia) in each bee species, the distribution of the frequencies of IPA loads pooled across the two screening occasions was plotted to determine the load threshold that discriminates covert infections (for which an ‘equilibrium’ exists between the host and the IPA) from overt infections at high loads and sometimes associated with disease symptoms^72^. The threshold was estimated, for bimodal distributions, at the lowest load between the two modes, and calculated for unimodal distributions to define the 5% highest loads according to the following formula $\overline{x }+1.65 sd$ ($\overline{x }$ mean load, $sd$ standard deviation).

After checking for normality and variance homogeneity, the loads at screening T0 of each frequently detected IPA in *A. mellifera* (viruses and honey bee microsporidia) and *B. terrestris* (viruses) were analysed with ANOVA models including the country and the site as factors. To analyse the changes in IPA loads upon field exposure, the difference between the load at T1 and the corresponding load at T0 was calculated for each sampled site. In order to have a balanced data set, in those instances where the IPA was not detected (and thus no load recorded), the value of the detection limit of the molecular method was assigned (Supplementary table S30), rather than using zero. For a small number of samples, when both the non-quantified load at T0 was replaced by the detection limit and the load at T1 was lower than the detection limit, then the negative load change was replaced by zero because of the low quantification accuracy below the detection limit. For each sentinel bee species, the overall change in loads of each IPA (quantitative data) between screening occasions was analysed with one-sample Student’s t tests against the mean = 0. After checking for normality, variance homogeneity and absence of strong correlations, the load changes of the frequently detected IPAs (viruses and honey bee microsporidia) were further analysed, first, to test for a sentinel bee species effect using a MANCOVA model including the country, the focal crop, the site (as fixed factors), the exposure time (as covariable), and their two-way interactions on IPA load changes of the three sentinel bee species together. Changes in the loads of these IPAs were then analysed for each sentinel bee species separately with a MANCOVA model including the country, the focal crop, the site (as fixed effects), the exposure time (as covariable), and their two-way interactions. Finally, the IPA-specific effects of the country, the focal crop, the site (as fixed effects) and the exposure time (as covariable) were analysed for each of the frequently detected IPAs in each sentinel bee species with ANCOVA models using a stepwise backward procedure. Where appropriate, post-hoc pairwise comparisons were performed with Tukey’s tests.

The relationship between the loads of several relevant IPAs detected in one bee species at screening T1 (viruses and microsporidia) and of *V. destructor*, as well as the relationship between the loads of IPAs detected in co-located sentinel bee species to explore the potential transmission between sentinels, were tested with Pearson’s test (or Spearman’s for small numbers of data points and non-normal distributions). For this latter analysis, the relationships were tested on both raw data (log_10_ loads) and loads corrected for the differences among countries (centring and standardization of the loads within each country as follows: (load–mean load)/standard deviation; Supplementary table S26). All the statistical analyses were performed using R software, version 3.5.2^126^, using the functions chisq.test, cor.test, t.test, wilcox.test, lm, anova, manova, TukeyHSD.

### Supplementary Information

Supplementary Information 1.

Supplementary Information 2.

## Data Availability

All data generated or analysed during this study are included in this published article as supplementary material.
