# Supplementary material for: Distribution of infectious and parasitic agents among three sentinel bee species across European agricultural landscapes
Source: Sci Rep. 2024 Feb 12;14:3524. doi: 10.1038/s41598-024-53357-w (PMC10861508; doi:10.1038/s41598-024-53357-w)
Supplement: Supplementary file 1 — Supplementary Information 1. [file 41598_2024_53357_MOESM1_ESM.docx]

**Distribution of infectious and parasitic agents among three sentinel bee species across European agricultural landscapes**

Supplementary material

**Supplementary table S1. Detection frequencies (percentage of positive sites) of the 11 IPAs on the two screening occasions, T0 and T1, in the three sentinel bees.** *N* indicates the number of sampled sites. The asterisk indicates a significant difference (*P* < 0.05) between the two screening occasions tested with χ^2^, and Fisher’s test for numbers of positive sites ≤ 5 (superscript *F*), NA indicates when the test could not be performed.

| **IPA** | ***Apis mellifera*** | | | ***Bombus terrestris*** | | | ***Osmia bicornis*** | | |
| --- | --- | --- | --- | --- | --- | --- | --- | --- | --- |
|  | **T0** (*N*=128) | **T1** (*N*=127) | ***P* value** | **T0** (*N*=128) | **T1** (*N*=122) | ***P* value** | **T0** (*N*=5) | **T1** (*N*=69) | ***P* value** |
| ABPV | 14.8 | 11.8 | 0.60 | 18.0 | 3.3 | <0.001* | 0.0 | 0.0 | NA |
| BQCV | 99.2 | 100 | 1 | 56.2 | 95.1 | <0.001* | 0.0 | 66.7 | <0.001* |
| CBPV | 35.2 | 34.7 | 1 | 14.8 | 5.7 | 0.03 | 0.0 | 0.0 | NA |
| DWV-A | 43.8 | 48.8 | 0.49 | 7.8 | 14.8 | 0.12 | 0.0 | 20.3 | <0.001* |
| DWV-B | 95.3 | 94.5 | 0.99 | 22.7 | 73.8 | <0.001* | 0.0 | 46.4 | <0.001* |
| SBV | 82.8 | 80.3 | 0.72 | 31.3 | 76.2 | <0.001* | 0.0 | 66.7 | <0.001* |
| *P. larvae* | 1.6 | 0.8 | 1*^F^* | 0.0 | 0.0 | NA | 0.0 | 0.0 | NA |
| *M. plutonius* | 5.5 | 3.9 | 0.78 | 0.0 | 0.0 | NA | 0.0 | 0.0 | NA |
| *N. apis* | 21.1 | 14.2 | 0.20 | 0.0 | 0.0 | NA | 0.0 | 0.0 | NA |
| *N. ceranae* | 57.8 | 67.8 | 0.13 | 0.8 | 0.0 | 1*^F^* | 0.0 | 1.5 | 1*^F^* |
| *N. bombi* | 0.0 | 0.0 | NA | 0.0 | 3.3 | 0.06*^F^* | 0.0 | 0.0 | NA |

**Supplementary table S2. Presence of the 11 IPAs in *Apis mellifera* as the number of positive sites in each country on each screening occasion.** Abbreviations: CHE=Switzerland, ESP=Spain, EST=Estonia, GBR=United Kingdom, GER=Germany, IRL=Ireland, ITA=Italy, SWE=Sweden, Pl=*Paenibacillus larvae* (causative agent of AFB), Mp=*Melissococcus plutonius* (causative agent of EFB), Na=*Nosema apis*, Nc=*Nosema ceranae*, Nb=*Nosema bombi*. Colour code: grey: no positive site, light yellow: 1 to 4 positive sites, medium yellow: 5 to 8 positive sites, dark yellow: 9 to 12 positive sites, and orange: 13 to 16 positive sites. CV is the coefficient of variation of site numbers for each IPA, calculated as the standard deviation divided by the mean.

| **Sampling** | **IPA** | **CHE** | **ESP** | **EST*** | **GBR** | **GER** | **IRL** | **ITA** | **SWE** | **CV (%)** |
| --- | --- | --- | --- | --- | --- | --- | --- | --- | --- | --- |
| T0 (*N*=128) | ABPV | 6 | 2 | 5 | 5 | 1 | 0 | 0 | 0 | 107.8 |
|  | BQCV | 16 | 16 | 16 | 16 | 16 | 15 | 16 | 16 | 2.2 |
|  | CBPV | 1 | 13 | 5 | 5 | 6 | 0 | 15 | 0 | 101.4 |
|  | DWV-A | 0 | 16 | 14 | 4 | 0 | 5 | 1 | 16 | 102.2 |
|  | DWV-B | 16 | 16 | 16 | 16 | 15 | 16 | 11 | 16 | 11.5 |
|  | SBV | 16 | 15 | 13 | 16 | 10 | 4 | 16 | 16 | 32.5 |
|  | Pl | 0 | 0 | 1 | 0 | 0 | 1 | 0 | 0 | 185.2 |
|  | Mp | 0 | 0 | 0 | 7 | 0 | 0 | 0 | 0 | 282.8 |
|  | Na | 0 | 4 | 11 | 8 | 0 | 3 | 0 | 1 | 122.6 |
|  | Nc | 16 | 10 | 13 | 14 | 11 | 0 | 8 | 2 | 61.4 |
|  | Nb | 0 | 0 | 0 | 0 | 0 | 0 | 0 | 0 | - |
| T1  (*N*=127) | ABPV | 5 | 5 | 1 | 0 | 2 | 0 | 0 | 2 | 112.0 |
|  | BQCV | 16 | 16 | 15 | 16 | 16 | 16 | 16 | 16 | 2.2 |
|  | CBPV | 8 | 7 | 5 | 6 | 9 | 0 | 9 | 0 | 66.6 |
|  | DWV-A | 3 | 16 | 12 | 6 | 0 | 8 | 1 | 16 | 82.4 |
|  | DWV-B | 15 | 16 | 11 | 16 | 16 | 16 | 14 | 16 | 11.8 |
|  | SBV | 16 | 7 | 12 | 16 | 15 | 4 | 16 | 16 | 37.2 |
|  | Pl | 0 | 0 | 0 | 0 | 0 | 1 | 0 | 0 | 282.8 |
|  | Mp | 0 | 0 | 0 | 5 | 0 | 0 | 0 | 0 | 282.8 |
|  | Na | 0 | 0 | 9 | 6 | 0 | 2 | 0 | 1 | 151.7 |
|  | Nc | 16 | 7 | 12 | 13 | 14 | 2 | 14 | 8 | 43.6 |
|  | Nb | 0 | 0 | 0 | 0 | 0 | 0 | 0 | 0 | - |

*At T1, 15 sites (samples of pooled bees) were analysed for Estonia.

**Supplementary table S3. IPA richness (number of detected IPAs) before field deployment (T0) and change between the screening occasions T0 and T1 overall and at the country scale for each sentinel bee (mean ± se)**

| **Bee species** | | **Mean±se** | **Min** | **Max** |
| --- | --- | --- | --- | --- |
| ***Apis mellifera*** | T0 (*N*=128) | 4.6±0.1 | 2 | 8 |
|  | Change (*N*=127) | +0.05±0.11 | -3 | +4 |
| ***Bombus terrestris*** | T0 (*N*=128) | 1.5±0.1 | 0 | 6 |
|  | Change (*N*=122) | +1.27±0.15 | -4 | +6 |
| ***Osmia bicornis*** | T0 (*N*=5) | NA | 0 | 0 |
|  | Change (*N*=69) | +2.01±0.14 | 0 | +4 |

| **Bee species** | | **CHE** | **ESP** | **EST** | **GBR** | **GER** | **IRL** | **ITA** | **SWE** |
| --- | --- | --- | --- | --- | --- | --- | --- | --- | --- |
| ***Apis***  ***mellifera*** | T0 | 4.4  ±0.1 | 5.8  ±0.3 | 5.9  ±0.3 | 5.7  ±0.2 | 3.7  ±0.2 | 2.8  ±0.2 | 4.2  ±0.3 | 4.2  ±0.1 |
|  | Change | +0.5  ±0.2 | -1.1  ±0.3 | -0.4  ±0.4 | -0.4  ±0.3 | +0.8  ±0.2 | +0.3  ±0.3 | +0.2  ±0.3 | +0.5  ±0.2 |
| ***Bombus***  ***terrestris*** | T0 | 0.8  ±0.2 | 1.1  ±0.3 | 1.4  ±0.3 | 0.8  ±0.2 | 0.4  ±0.2 | 1.6  ±0.3 | 4.3  ±0.2 | 1.5  ±0.2 |
|  | Change | +2.0  ±0.2 | +1.4  ±0.3 | +0.9  ±0.4 | +2.6  ±0.2 | +2.3  ±0.2 | +0.1  ±0.3 | -1.2  ±0.6 | +1.9  ±0.2 |
| ***Osmia***  ***bicornis*** | T0 | 0.0 | 0.0 | 0.0 | 0.0 | 0.0 | 0.0 | 0.0 | 0.0 |
|  | Change | +1.8  ±0.3 | +1.7  ±0.3 | +2.1  ±0.2 |  | +1.5  ±0.4 |  |  | +2.7  ±0.3 |

**Supplementary table S4. IPA diversity with the Shannon index.** The diversity of detected IPAs was estimated using the formula of the Shannon index$H^{'}= - \sum_{i}^{S} pi . ln(pi)$, with *pi* the IPA detection frequency (proportion of positive samples among analysed samples) and S the total number of IPAs. This index combines the information of the IPA presence (richness) and their abundance across samples (detection frequency). It ranges from 0 (all the IPAs are present in all the samples but without heterogeneity) to ln(S).

The index was calculated at the species and country scales for the two screening occasions (except T0 for *O. bicornis* since none of the pathogens were detected) and at the focal crop scale at T1. The higher the *H’* value, the higher the diversity and heterogeneity of the detected IPAs. However, similar richness values do not automatically yield similar diversity values: high detection frequencies are associated with low IPA heterogeneity and do not contribute as strongly to increasing the *H’* index value as low and moderate detection frequencies (a frequency of 1 yields a ln(p) value of 0). In a consistent manner, a positive change of the *H’* value upon sentinels’ deployment in the field would indicate increasing heterogeneity, while a negative change in the index value would indicate decreasing heterogeneity.

Before the sentinels’ deployment in the field, the IPA diversity was similar in *A. mellifera* and *B. terrestris* despite their difference in IPA richness (table below). The *A. mellifera* samples harboured a few highly frequent viruses (BQCV, DWV-B and SBV) while the *B. terrestris* samples harboured fewer IPAs at overall lower detection frequencies. For these two sentinel bees, the IPA diversity varied across countries without one country exhibiting higher or lower values.

Upon field exposure, the IPA diversity remained constant in *A. mellifera*, overall (Table below) and across most countries (one-sample t test, µ=0: *t*=0.88, *df*=7, *P*=0.41), consistently with the absence of average change in IPA richness or detection frequencies. By contrast, the IPA diversity decreased in *B. terrestris* overall (Table below) and across almost all the countries (one-sample t test, µ=0: *t*=-3.06, *df*=7, *P*=0.018), reflecting the increasing detection frequencies for most viruses. IPA diversity of *O. bicornis* after field exposure was similar to that of *B. terrestris*, reflecting the similarly low IPA richness but high detection frequencies for the four detected viruses.

| Sentinel | | Species | Country | | | | | | | | Crop | |
| --- | --- | --- | --- | --- | --- | --- | --- | --- | --- | --- | --- | --- |
|  |  |  | CHE | ESP | EST | GBR | GER | IRL | ITA | SWE | OSR | APP |
| *A. mellifera* | T0 | 2.09 | 0.54 | 1.13 | 1.61 | 1.90 | 1.15 | 1.26 | 0.84 | 0.43 |  |  |
|  | T1 | 1.94 | 1.08 | 1.15 | 1.85 | 1.64 | 0.76 | 1.39 | 0.73 | 0.78 | 1.73 | 1.98 |
| *B. terrestris* | T0 | 1.85 | 1.20 | 1.51 | 1.00 | 0.69 | 0.75 | 1.32 | 1.02 | 1.20 |  |  |
|  | T1 | 1.15 | 0.17 | 0.67 | 0.84 | 0.66 | 0.47 | 0.58 | 1.04 | 0.86 | 1.34 | 0.88 |
| *O. bicornis* | T0 |  |  |  |  |  |  |  |  |  |  |  |
|  | T1 | 1.28 | 0.85 | 0.95 | 0.91 |  | 0.98 |  |  | 0.88 |  |  |

**Supplementary table S5. Presence of the 11 IPAs in *Bombus terrestris* as the number of positive sites in each country on each screening occasion.**  Abbreviations: CHE=Switzerland, ESP=Spain, EST=Estonia, GBR=United Kingdom, GER=Germany, IRL=Ireland, ITA=Italy, SWE=Sweden, Pl=*Paenibacillus larvae* (causative agent of AFB), Mp=*Melissococcus plutonius* (causative agent of EFB), Na=*Nosema apis*, Nc=*Nosema ceranae*, Nb=*Nosema bombi*. Colour code: grey: no positive site, light yellow: 1 to 4 positive sites, medium yellow: 5 to 8 positive sites, dark yellow: 9 to 12 positive sites, and orange: 13 to 16 positive sites. CV is the coefficient of variation of site numbers for each IPA, calculated as the standard deviation divided by the mean.

| **Sampling** | **IPA** | **CHE** | **ESP** | **EST*** | **GBR** | **GER** | **IRL** | **ITA*** | **SWE** | **CV (%)** |
| --- | --- | --- | --- | --- | --- | --- | --- | --- | --- | --- |
| T0  (*N=*128) | ABPV | 3 | 4 | 0 | 0 | 0 | 5 | 10 | 1 | 121.1 |
|  | BQCV | 6 | 7 | 12 | 8 | 2 | 8 | 16 | 13 | 49.3 |
|  | CBPV | 0 | 2 | 0 | 0 | 3 | 0 | 14 | 0 | 203.7 |
|  | DWV-A | 0 | 1 | 2 | 0 | 1 | 0 | 3 | 3 | 102.5 |
|  | DWV-B | 2 | 0 | 1 | 0 | 0 | 10 | 10 | 6 | 121.5 |
|  | SBV | 2 | 6 | 8 | 4 | 0 | 3 | 16 | 1 | 103.1 |
|  | Pl | 0 | 0 | 0 | 0 | 0 | 0 | 0 | 0 | - |
|  | Mp | 0 | 0 | 0 | 0 | 0 | 0 | 0 | 0 | - |
|  | Na | 0 | 0 | 0 | 0 | 0 | 0 | 0 | 0 | - |
|  | Nc | 0 | 0 | 0 | 0 | 0 | 0 | 0 | 0 | - |
|  | Nb | 0 | 0 | 0 | 0 | 0 | 0 | 0 | 0 | - |
| T1  (*N*=122) | ABPV | 0 | 0 | 1 | 1 | 0 | 0 | 1 | 1 | 106.9 |
|  | BQCV | 15 | 16 | 11 | 16 | 16 | 11 | 15 | 16 | 15.2 |
|  | CBPV | 0 | 0 | 0 | 3 | 0 | 0 | 4 | 0 | 187.7 |
|  | DWV-A | 0 | 10 | 2 | 0 | 0 | 0 | 1 | 5 | 158.9 |
|  | DWV-B | 14 | 14 | 2 | 16 | 10 | 16 | 3 | 15 | 50.9 |
|  | SBV | 16 | 2 | 11 | 16 | 16 | 1 | 15 | 16 | 55.7 |
|  | Pl | 0 | 0 | 0 | 0 | 0 | 0 | 0 | 0 | - |
|  | Mp | 0 | 0 | 0 | 0 | 0 | 0 | 0 | 0 | - |
|  | Na | 0 | 0 | 0 | 0 | 0 | 0 | 0 | 0 | - |
|  | Nc | 0 | 0 | 0 | 0 | 0 | 0 | 0 | 0 | - |
|  | Nb | 0 | 0 | 0 | 1 | 1 | 0 | 0 | 2 | 151.2 |

*At T1, 11 sites (samples of pooled bees) were analysed for Estonia and 15 sites (samples) for Italy.

**Supplementary figure S1. IPA richness after field exposure (T1) in the three sentinel bees.** The line indicates equal IPA richness values in two sentinel bees.


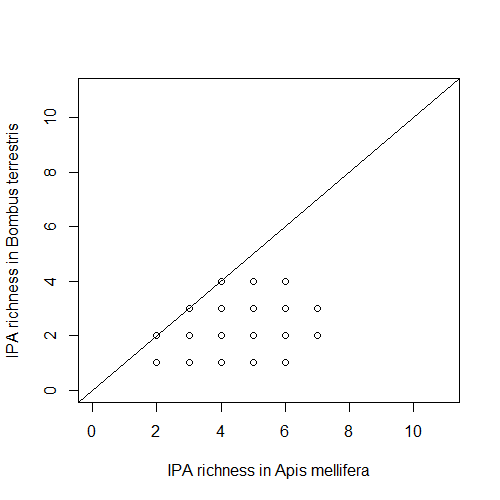

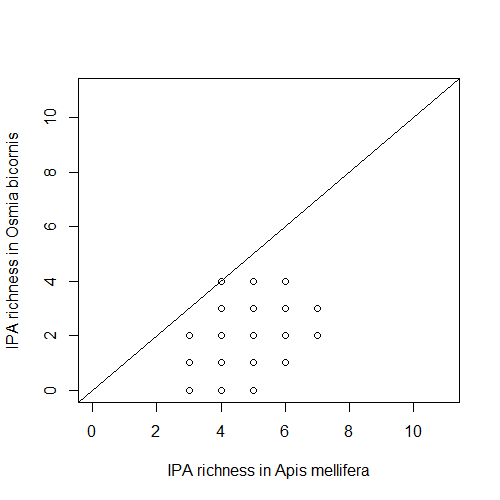

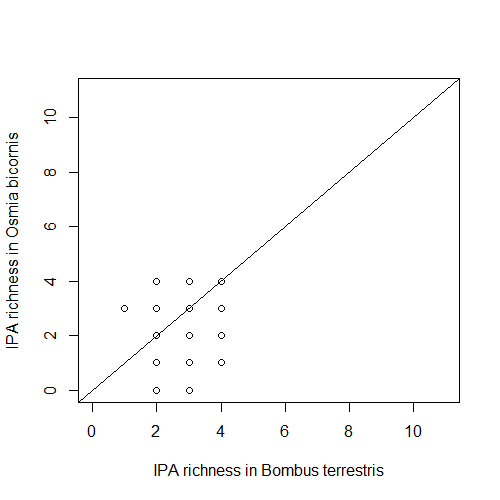


**Supplementary table S6. Presence of the 11 IPAs in *Osmia bicornis* as the number of positive sites in each country on each screening occasion**. Abbreviations: CHE=Switzerland, ESP=Spain, EST=Estonia, GER=Germany, SWE=Sweden, Pl=*Paenibacillus larvae* (causative agent of AFB), Mp=*Melissococcus plutonius* (causative agent of EFB), Na=*Nosema apis*, Nc=*Nosema ceranae*, Nb=*Nosema bombi*. Colour code: grey: no positive site, light yellow: 1^st^ quarter of positive sites, medium yellow: 2^nd^ quarter of positive sites, dark yellow: 3^rd^ quarter of positive sites, and orange: last quarter of positive sites. CV is the coefficient of variation of site numbers for each IPA, calculated as the standard deviation divided by the mean.

| **Sampling** | T0 (*N*=5) | Screening T1 (*N*=69) | | | | | |
| --- | --- | --- | --- | --- | --- | --- | --- |
| **IPA** | **Overall** | **CHE**  **(12 sites)** | **ESP**  **(14 sites)** | **EST**  **(15 sites)** | **GER**  **(13 sites)** | **SWE**  **(15 sites)** | **CV (%)** |
| ABPV | 0 | 0 | 0 | 0 | 0 | 0 | - |
| BQCV | 0 | 6 | 8 | 14 | 6 | 12 | 39.5 |
| CBPV | 0 | 0 | 0 | 0 | 0 | 0 | - |
| DWV-A | 0 | 0 | 7 | 2 | 0 | 5 | 111.2 |
| DWV-B | 0 | 6 | 9 | 2 | 5 | 10 | 50.1 |
| SBV | 0 | 10 | 0 | 13 | 9 | 14 | 60.2 |
| Pl | 0 | 0 | 0 | 0 | 0 | 0 | - |
| Mp | 0 | 0 | 0 | 0 | 0 | 0 | - |
| Na | 0 | 0 | 0 | 0 | 0 | 0 | - |
| Nc | 0 | 0 | 0 | 1 | 0 | 0 | 223.6 |
| Nb | 0 | 0 | 0 | 0 | 0 | 0 | - |

**Supplementary table S7.** Change in IPA richness between the two screening occasions (difference in IPA number between the two screening occasions) for each bee species on each focal crop (O: oilseed rape fields, A: apple orchards)

| **Bee species** | **Crop** | **Minimum** | **Maximum** | **Mean ± se** |
| --- | --- | --- | --- | --- |
| *A. mellifera* | **O** | -2 | 2 | 0.14 ± 0.02 |
|  | **A** | -3 | 4 | -0.04 ± 0.02 |
| *B. terrestris* | **O** | -3 | 6 | 1.48 ± 0.03 |
|  | **A** | -4 | 4 | 1.06 ± 0.03 |
| *O. bicornis* | **O** | 0 | 4 | 2.05 ± 0.04 |
|  | **A** | 0 | 4 | 1.97 ± 0.03 |

**Supplementary table S8.** Detection frequency of the 11 screened IPAs on the two focal crops (O: oilseed rape fields, A: apple orchards) at screening T1 (% positive sites). Pl=*Paenibacillus larvae*, Mp=*Melisoccocus plutonius*, Na=*Nosema apis*, Nc=*Nosema ceranae*, Nb=*Nosema bombi*. None of the frequency differences between focal crops were significant, tested for each IPA and bee species with χ^2^ (Fisher’s test for numbers of positive sites ≤ 5).

| **Bee species** | **Crop** | **ABPV** | **BQCV** | **CBPV** | **DWV-A** | **DWV-B** | **SBV** | **Pl** | **Mp** | **Na** | **Nc** | **Nb** |
| --- | --- | --- | --- | --- | --- | --- | --- | --- | --- | --- | --- | --- |
| *A. mellifera* | **O** | 10.9 | 100 | 26.6 | 48.4 | 95.3 | 82.8 | 1.6 | 7.8 | 18.8 | 68.8 | 0 |
|  | **A** | 12.7 | 100 | 42.9 | 49.2 | 93.7 | 77.8 | 0 | 0 | 9.5 | 66.7 | 0 |
| *B. terrestris* | **O** | 1.7 | 93.3 | 1.7 | 10.0 | 81.7 | 75.0 | 0 | 0 | 0 | 0 | 1.7 |
|  | **A** | 4.8 | 96.8 | 9.7 | 19.4 | 66.1 | 77.4 | 0 | 0 | 0 | 0 | 4.8 |
| *O. bicornis* | **O** | 0 | 70.3 | 0 | 21.6 | 43.2 | 67.6 | 0 | 0 | 0 | 2.7 | 0 |
|  | **A** | 0 | 62.5 | 0 | 18.8 | 50.0 | 65.6 | 0 | 0 | 0 | 0 | 0 |

**Supplementary table S9.** IPA detection frequencies at T1 on each focal crop (O: oilseed rape fields, A: apple orchards) in each country (% positive sites).

| Bee species | Country | Crop | ABPV | BQCV | CBPV | DWV-A | DWV-B | SBV | Pl | Mp | Na | Nc | Nb |
| --- | --- | --- | --- | --- | --- | --- | --- | --- | --- | --- | --- | --- | --- |
| *A. mellifera* | GBR | O | 0 | 100 | 12.5 | 25.0 | 100 | 100 | 0 | 62.5 | 75.0 | 87.5 | 0 |
|  |  | A | 0 | 100 | 62.5 | 50.0 | 100 | 100 | 0 | 0 | 0 | 75.0 | 0 |
|  | ESP | O | 50.0 | 100 | 37.5 | 100 | 100 | 50,0 | 0 | 0 | 0 | 50.0 | 0 |
|  |  | A | 12.5 | 100 | 50.0 | 100 | 100 | 37.5 | 0 | 0 | 0 | 37.5 | 0 |
|  | CHE | O | 12.5 | 100 | 62.5 | 37.5 | 87.5 | 100 | 0 | 0 | 0 | 100 | 0 |
|  |  | A | 50.0 | 100 | 37.5 | 0 | 100 | 100 | 0 | 0 | 0 | 100 | 0 |
|  | ITA | O | 0 | 100 | 25.0 | 0 | 100 | 100 | 0 | 0 | 0 | 75.0 | 0 |
|  |  | A | 0 | 100 | 87.5 | 12.5 | 75.0 | 100 | 0 | 0 | 0 | 100 | 0 |
|  | GER | O | 0 | 100 | 50.0 | 0 | 100 | 87.5 | 0 | 0 | 0 | 87.5 | 0 |
|  |  | A | 25.0 | 100 | 62.5 | 0 | 100 | 100 | 0 | 0 | 0 | 87.5 | 0 |
|  | IRL | O | 0 | 100 | 0 | 50.0 | 100 | 37.5 | 12.5 | 0 | 12.5 | 0 | 0 |
|  |  | A | 0 | 100 | 0 | 50.0 | 100 | 12.5 | 0 | 0 | 12.5 | 25.0 | 0 |
|  | EST | O | 12.5 | 100 | 25.0 | 75.0 | 75.0 | 87.5 | 0 | 0 | 50.0 | 87.5 | 0 |
|  |  | A | 0 | 100 | 42.9 | 85.7 | 71.4 | 71.4 | 0 | 0 | 71.4 | 71.4 | 0 |
|  | SWE | O | 12.5 | 100 | 0 | 100 | 100 | 100 | 0 | 0 | 12.5 | 62.5 | 0 |
|  |  | A | 12.5 | 100 | 0 | 100 | 100 | 100 | 0 | 0 | 0 | 37.5 | 0 |
| *B. terrestris* | GBR | O | 12.5 | 100 | 0 | 0 | 100 | 100 | 0 | 0 | 0 | 0 | 0 |
|  |  | A | 0 | 100 | 37.5 | 0 | 100 | 100 | 0 | 0 | 0 | 0 | 12.5 |
|  | ESP | O | 0 | 100 | 0 | 50.0 | 87.5 | 0 | 0 | 0 | 0 | 0 | 0 |
|  |  | A | 0 | 100 | 0 | 75.0 | 87.5 | 25.0 | 0 | 0 | 0 | 0 | 0 |
|  | CHE | O | 0 | 100 | 0 | 0 | 100 | 100 | 0 | 0 | 0 | 0 | 0 |
|  |  | A | 0 | 87.5 | 0 | 0 | 75.0 | 100 | 0 | 0 | 0 | 0 | 0 |
|  | ITA | O | 0 | 100 | 14.3 | 0 | 28.6 | 100 | 0 | 0 | 0 | 0 | 0 |
|  |  | A | 12.5 | 100 | 37.5 | 12.5 | 12.5 | 100 | 0 | 0 | 0 | 0 | 0 |
|  | GER | O | 0 | 100 | 0 | 0 | 100 | 100 | 0 | 0 | 0 | 0 | 12.5 |
|  |  | A | 0 | 100 | 0 | 0 | 25.0 | 100 | 0 | 0 | 0 | 0 | 0 |
|  | IRL | O | 0 | 50.0 | 0 | 0 | 100 | 12.5 | 0 | 0 | 0 | 0 | 0 |
|  |  | A | 0 | 87.5 | 0 | 0 | 100 | 0 | 0 | 0 | 0 | 0 | 0 |
|  | EST | O | 0 | 100 | 0 | 20.0 | 0 | 100 | 0 | 0 | 0 | 0 | 0 |
|  |  | A | 16.7 | 100 | 0 | 16.7 | 33.3 | 100 | 0 | 0 | 0 | 0 | 0 |
|  | SWE | O | 0 | 100 | 0 | 12.5 | 100 | 100 | 0 | 0 | 0 | 0 | 0 |
|  |  | A | 12.5 | 100 | 0 | 50.0 | 87.5 | 100 | 0 | 0 | 0 | 0 | 25.0 |
| *O. bicornis* | ESP | O | 0 | 50.0 | 0 | 37.5 | 37.5 | 0 | 0 | 0 | 0 | 0 | 0 |
|  |  | A | 0 | 66.7 | 0 | 66.7 | 100 | 0 | 0 | 0 | 0 | 0 | 0 |
|  | CHE | O | 0 | 62.5 | 0 | 0 | 37.5 | 75.0 | 0 | 0 | 0 | 0 | 0 |
|  |  | A | 0 | 25.0 | 0 | 0 | 75.0 | 100 | 0 | 0 | 0 | 0 | 0 |
|  | GER | O | 0 | 83.3 | 0 | 0 | 66.7 | 100 | 0 | 0 | 0 | 0 | 0 |
|  |  | A | 0 | 14.3 | 0 | 0 | 14.3 | 42.9 | 0 | 0 | 0 | 0 | 0 |
|  | EST | O | 0 | 85.7 | 0 | 14.3 | 14.3 | 85.7 | 0 | 0 | 0 | 14.3 | 0 |
|  |  | A | 0 | 100 | 100 | 12.5 | 12.5 | 87.5 | 0 | 0 | 0 | 0 | 0 |
|  | SWE | O | 0 | 75.0 | 0 | 50.0 | 62.5 | 87.5 | 0 | 0 | 0 | 0 | 0 |
|  |  | A | 0 | 85.7 | 0 | 14.3 | 71.4 | 100 | 0 | 0 | 0 | 0 | 0 |

**Supplementary figure S2. Synthetic index summarizing the risk of IPA exposure at each site for each sentinel bee.** To summarize the exposure of bees to the most prevalent IPAs at each sampled site and associate a risk to this exposure, a raw exposure risk index was calculated using the load thresholds of each IPA and following the formula: IPA load / IPA threshold. For each IPA at each screening occasion, risks were encoded for data interpretation to fall into four infection risk categories by the IPA threshold and the uncertainty of the molecular method *U* (see Supplementary table S21) as described below:

*IPA load* *Risk*


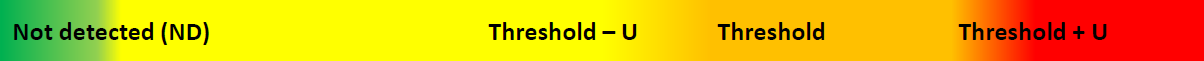

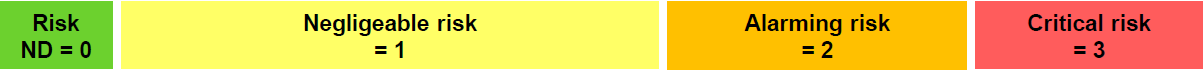


These categorial values were then included into a Multiple Factorial Analysis (MFA) to yield a single exposure risk value per site. The synthetic risk value for one bee species sampled at a specific site corresponds to the projection of the site data point onto the first dimension of the MFA, *i.e.* the dimension that explains the largest part of the total variability. Synthetic risk indices range from negative to positive values, each associated with exposure risks to specific IPAs. The synthetic risk indices and the interpretation of raw data based on risk categories enabled to visualize whether the two focal crops differed in their exposure risk profiles, and to discriminate the countries based on their exposure risk profiles at the two screening occasions, T0 and T1.

For *A. mellifera*, the analysis was performed on the loads of eight IPAs, including the six viruses and the two honey bee microsporidia. The first MFA dimension explained 12% of the index variability, and the most contributing (>2.5%) and best representing (Cos2>0.5) IPAs were DWV-B and *N. ceranae* (see histograms below).


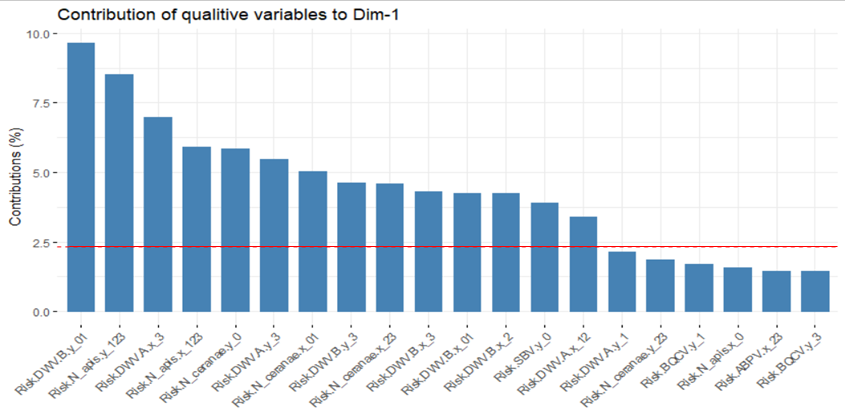

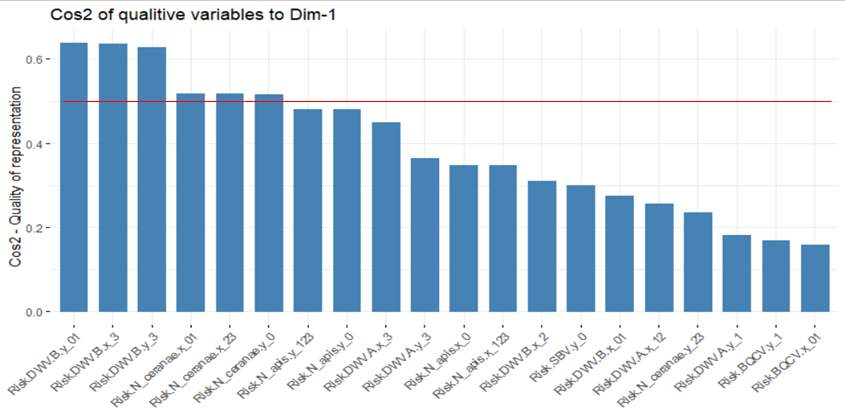


Contribution (top, in %) and quality of representation (bottom, Cos2 value) of each IPA in the first dimension of the MFA on *Apis mellifera* raw exposure risk indices. ‘x’ corresponds to the risk at T0, ‘y’ to the risk at T1.

For *B. terrestris*, the analysis was performed on the loads of the six viruses. The first MFA dimension explained 13% of the total variability. The most contributing (>2.5%) and best representing (Cos2>0.5) IPAs were BQCV at T0, DWV-B at T1, and CBPV and SBV at both screening occasions (see histograms below).


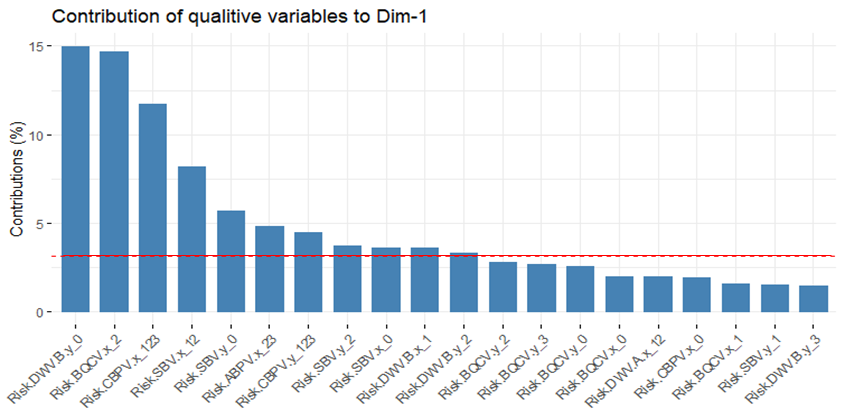

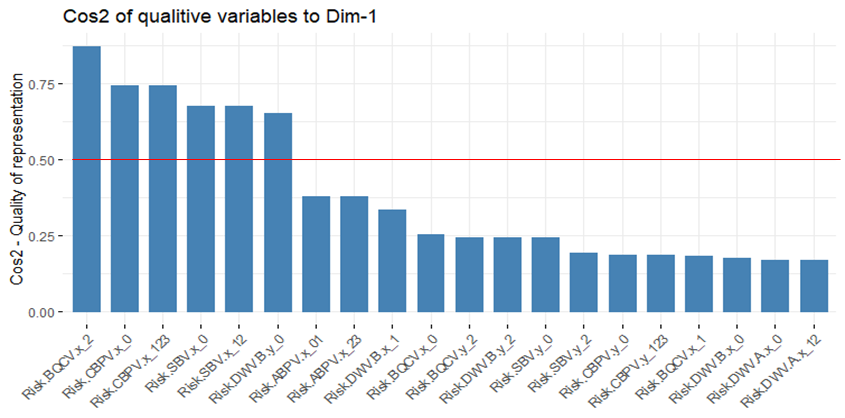


Contribution (top, in %) and quality of representation (bottom, Cos2 value) of each IPA in the first dimension of the MFA on *Bombus terrestris* raw exposure risk indices. ‘x’ corresponds to the risk at T0, ‘y’ to the risk at T1.

For *O. bicornis*, the analysis was performed on the loads of the four viruses, BQCV, DWV-A, DWV-B and SBV at T1. The first MFA dimension explained 27% of the variability. The most contributing (>2.5%) and best representing (Cos2>0.5) IPAs were BQCV and DWV-B (see histograms below).


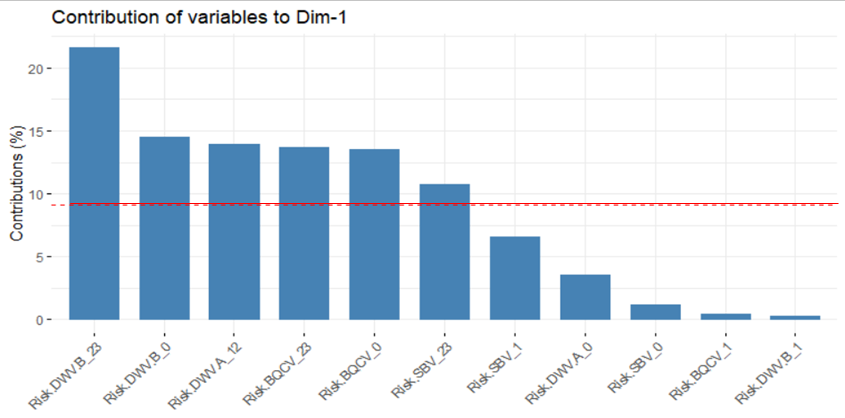

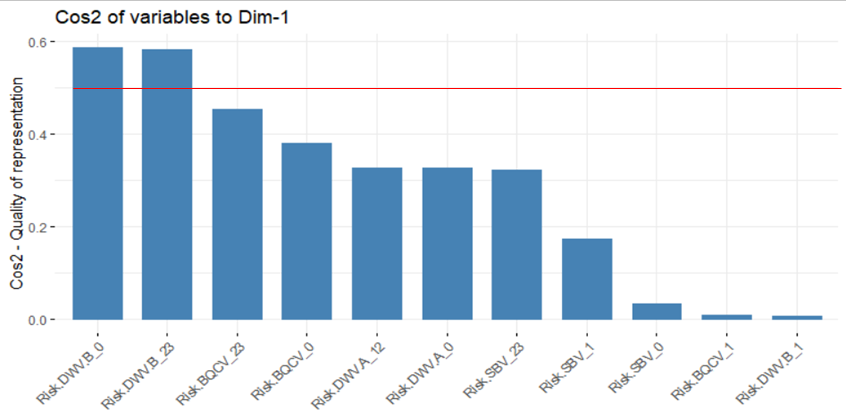


Contribution (top, in %) and quality of representation (bottom, Cos2 value) of each IPA in the first dimension of the MFA on *Osmia bicornis* raw exposure risk indices. ‘x’ corresponds to the risk at T0, ‘y’ to the risk at T1.

**Supplementary table S10.** Distribution of the samples with negative exposure risk (N) in the four risk categories (C; 0 to 3 or clustered categories) for the eight IPAs in *Apis mellifera*.

|  | ABPV | | BQCV | | CBPV | | DWV-A | | DWV-B | | SBV | | *N. apis* | | *N. ceranae* | |
| --- | --- | --- | --- | --- | --- | --- | --- | --- | --- | --- | --- | --- | --- | --- | --- | --- |
|  | **C** | ***N*** | **C** | ***N*** | **C** | ***N*** | **C** | ***N*** | **C** | ***N*** | **C** | ***N*** | **C** | ***N*** | **C** | ***N*** |
| T0 | **0-1** | 22 | **0-1** | 15 | **0-1** | 19 | **0** | 7 | **0-1** | 0 | **0** | 7 | **0** | 22 | **0-1** | 20 |
|  | **2-3** | 0 | **2** | 5 | **2-3** | 3 | **1-2** | 0 | **2** | 0 | **1** | 11 | **1-2-3** | 0 | **2-3** | 2 |
|  |  |  | **3** | 5 |  |  | **3** | 15 | **3** | 22 | **2-3** | 4 |  |  |  |  |
| T1 | **0** | 21 | **1** | 14 | **0** | 20 | **0** | 5 | **0-1** | 0 | **0** | 14 | **0** | 22 | **0** | 19 |
|  | **1-2-3** | 1 | **2** | 2 | **1** | 2 | **1** | 0 | **2** | 2 | **1-2** | 1 | **1-2-3** | 0 | **1** | 3 |
|  |  |  | **3** | 6 | **2-3** | 0 | **2** | 4 | **3** | 20 | **3** | 7 |  |  | **2-3** | 0 |
|  |  |  |  |  |  |  | **3** | 13 |  |  |  |  |  |  |  |  |

**Supplementary table S11.** Distribution of the samples with positive exposure risk (N) in the four risk categories (C; 0 to 3 or clustered categories) for the eight IPAs in *Apis mellifera*.

|  | ABPV | | BQCV | | CBPV | | DWV-A | | DWV-B | | SBV | | *N.apis* | | *N.ceranae* | |
| --- | --- | --- | --- | --- | --- | --- | --- | --- | --- | --- | --- | --- | --- | --- | --- | --- |
|  | **C** | ***N*** | **C** | ***N*** | **C** | ***N*** | **C** | ***N*** | **C** | ***N*** | **C** | ***N*** | **C** | ***N*** | **C** | ***N*** |
| T0 | **0-1** | 20 | **0-1** | 4 | **0-1** | 17 | **0** | 13 | **0-1** | 9 | **0** | 1 | **0** | 7 | **0-1** | 2 |
|  | **2-3** | 3 | **2** | 8 | **2-3** | 6 | **1-2** | 10 | **2** | 10 | **1** | 10 | **1-2-3** | 16 | **2-3** | 21 |
|  |  |  | **3** | 11 |  |  | **3** |  | **3** | 4 | **2-3** | 12 |  |  |  |  |
| T1 | **0** | 22 | **1** | 2 | **0** | 15 | **0** | 14 | **0-1** | 16 | **0** | 2 | **0** | 9 | **0** | 1 |
|  | **1-2-3** | 1 | **2** | 4 | **1** | 3 | **1** | 7 | **2** | 3 | **1-2** | 3 | **1-2-3** | 14 | **1** | 9 |
|  |  |  | **3** | 17 | **2-3** | 5 | **2** | 1 | **3** | 4 | **3** | 18 |  |  | **2-3** | 13 |
|  |  |  |  |  |  |  | **3** | 1 |  |  |  |  |  |  |  |  |

**Supplementary table S12.** Distribution of the samples with negative exposure risk (N) in the four risk categories (C; 0 to 3 or clustered categories) for the six IPAs in *Bombus terrestris*.

|  | ABPV | | BQCV | | CBPV | | DWV-A | | DWV-B | | SBV | |
| --- | --- | --- | --- | --- | --- | --- | --- | --- | --- | --- | --- | --- |
|  | **C** | ***N*** | **C** | ***N*** | **C** | ***N*** | **C** | ***N*** | **C** | ***N*** | **C** | ***N*** |
| T0 | **0-1** | 6 | **0** | 5 | **0** | 7 | **0** | 7 | **0** | 5 | **0** | 7 |
|  | **2-3** | 1 | **1** | 2 | **1-2-3** | 0 | **1-2** | 0 | **1** | 0 | **1-2** | 0 |
|  |  |  | **2** | 0 |  |  |  |  | **2-3** | 2 |  |  |
| T1 | **0** | 7 | **0** | 4 | **0** | 7 | **0** | 7 | **0** | 0 | **0** | 7 |
|  | **1-2-3** | 0 | **1** | 1 | **1-2-3** | 0 | **1-2-3** | 0 | **1** | 3 | **1** | 0 |
|  |  |  | **2** | 0 |  |  |  |  | **2** | 4 | **2** | 0 |
|  |  |  | **3** | 2 |  |  |  |  | **3** | 0 | **3** | 0 |

**Supplementary table S13.** Distribution of the samples with positive exposure risk (N) in the four risk categories (C; 0 to 3 or clustered categories) for the six IPAs in *Bombus terrestris*.

|  | ABPV | | BQCV | | CBPV | | DWV-A | | DWV-B | | SBV | |
| --- | --- | --- | --- | --- | --- | --- | --- | --- | --- | --- | --- | --- |
|  | **C** | ***N*** | **C** | ***N*** | **C** | ***N*** | **C** | ***N*** | **C** | ***N*** | **C** | ***N*** |
| T0 | **0-1** | 8 | **0** | 1 | **0** | 6 | **0** | 15 | **0** | 7 | **0** | 0 |
|  | **2-3** | 10 | **1** | 0 | **1-2-3** | 12 | **1-2** | 3 | **1** | 8 | **1-2** | 18 |
|  |  |  | **2** | 17 |  |  |  |  | **2-3** | 3 |  |  |
| T1 | **0** | 18 | **0** | 0 | **0** | 15 | **0** | 15 | **0** | 15 | **0** | 0 |
|  | **1-2-3** | 0 | **1** | 3 | **1-2-3** | 3 | **1-2-3** | 3 | **1** | 2 | **1** | 4 |
|  |  |  | **2** | 14 |  |  |  |  | **2** | 1 | **2** | 8 |
|  |  |  | **3** | 1 |  |  |  |  | **3** | 0 | **3** | 6 |

**Supplementary table S14.** Distribution of the samples with negative exposure risk (N) in the four risk categories (C; 0 to 3 or clustered categories) for the six IPAs in *Osmia bicornis*.

|  | BQCV | | DWV-A | | DWV-B | | SBV | |
| --- | --- | --- | --- | --- | --- | --- | --- | --- |
|  | **C** | ***N*** | **C** | ***N*** | **C** | ***N*** | **C** | ***N*** |
| T1 | **0** | 16 | **0** | 16 | **0** | 16 | **0** | 11 |
|  | **1** | 0 | **1-2** | 0 | **1** | 0 | **1** | 5 |
|  | **2-3** | 0 |  |  | **2-3** | 0 | **2-3** | 0 |

**Supplementary table S15.** Distribution of the samples with positive exposure risk (N) in the four risk categories (C; 0 to 3 or clustered categories) for the six IPAs in *Osmia bicornis*.

|  | BQCV | | DWV-A | | DWV-B | | SBV | |
| --- | --- | --- | --- | --- | --- | --- | --- | --- |
|  | **C** | ***N*** | **C** | ***N*** | **C** | ***N*** | **C** | ***N*** |
| T1 | **0** | 0 | **0** | 5 | **0** | 0 | **0** | 2 |
|  | **1** | 1 | **1-2** | 7 | **1** | 1 | **1** | 1 |
|  | **2-3** | 11 |  |  | **2-3** | 11 | **2-3** | 9 |

**Supplementary figure S3.** Frequency of exposure risk indices on apple (in blue) and oilseed rape (orange) for (A) *Apis mellifera*, (B) *Bombus terrestris* and (C) *Osmia bicornis*.


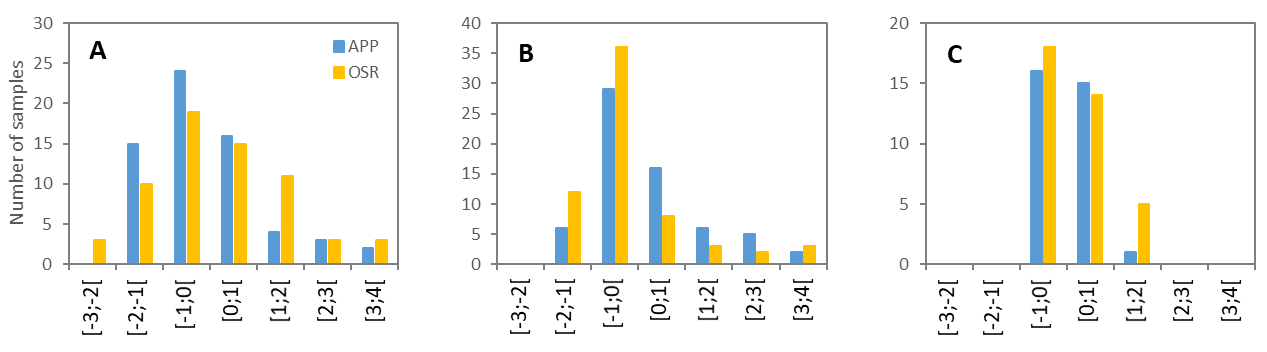


**Supplementary table S16.** Results of the ANOVA analysis (*P* values) of the IPA loads quantified in *Apis mellifera* and *Bombus terrestris* at screening T0. The significance threshold of *P* values was set at α=0.05.

| **Source of variation** | **ABPV** | **BQCV** | **CBPV** | **DWV-A** | **DWV-B** | **SBV** | **Na** | **Nc** |
| --- | --- | --- | --- | --- | --- | --- | --- | --- |
| ***Apis mellifera*** |  |  |  |  |  |  |  |  |
| Country | **0.040** | **<0.0001** | **0.005** | **<0.0001** | **<0.0001** | **<0.0001** | **<0.0001** | **<0.0001** |
| Site | 0.13 | 0.97 | 0.72 | 0.41 | 0.45 | 0.96 | 0.92 | 0.71 |
| ***Bombus terrestris*** |  |  |  |  |  |  |  |  |
| Country | **<0.0001** | **<0.0001** | 0.09 | **0.047** | **0.012** | **<0.0001** |  |  |
| Site | **0.041** | 0.42 | 0.40 | 0.79 | 0.22 | 0.84 |  |  |

**Supplementary table S17.** Results of the MANCOVA analysis of the load changes between the screening occasions T1 and T0 for the eight most frequent IPAs (six viruses, and two honey bee microsporidia *N. apis* and *N. ceranae*) in the three bee species. Similar results were obtained when including the 11 IPAs. The significance threshold of *P* values was set at α=0.05.

| **Source of variation** | ***df*** | ***Approximate F*** | ***P*** |
| --- | --- | --- | --- |
| Bee species | 16, 512 | 6.35 | **<0.0001** |
| Country | 56, 1827 | 4.60 | **<0.0001** |
| Crop | 8, 255 | 1.44 | 0.18 |
| Exposure time | 8, 255 | 5.07 | **<0.0001** |
| Site | 112, 2096 | 0.81 | 0.93 |
| Bee species × Country | 88, 2096 | 3.12 | **<0.0001** |
| Bee species × Crop | 16, 512 | 0.71 | 0.78 |
| Country × Crop | 56, 1827 | 2.01 | **<0.0001** |
| Bee species × Exposure time | 16, 512 | 1.15 | 0.31 |
| Country × Exposure time | 56, 1827 | 1.16 | 0.20 |
| Crop × Exposure time | 8, 255 | 1.90 | 0.06 |

**Supplementary table S18.** Results of the MANCOVA analysis of the load changes between screening occasions T1 and T0 for the eight most frequent IPAs (six viruses and two honey bee microsporidia) in each bee species. IPAs included the six viruses and the two honey bee microsporidia for *A. mellifera*, the six viruses for *B. terrestris* and the viruses BQCV, DWV-A, DWV-B and SBV for *O. bicornis.* The significance threshold of *P* values was set at α=0.05.

| **Source of variation** | ***df*** | ***Approximate F*** | ***P*** |
| --- | --- | --- | --- |
| ***Apis mellifera*** |  |  |  |
| Country | 56, 609 | 3.04 | **<0.0001** |
| Crop | 8, 81 | 0.81 | 0.59 |
| Exposure time | 8, 81 | 2.49 | **0.018** |
| Site | 112, 704 | 0.74 | 0.98 |
| Country × Crop | 56, 609 | 1.36 | **0.048** |
| Country × Exposure time | 56, 609 | 0.93 | 0.63 |
| Crop × Exposure time | 8, 81 | 1.01 | 0.43 |
| ***Bombus terrestris*** |  |  |  |
| Country | 42, 912 | 5.85 | **<0.0001** |
| Crop | 6, 147 | 2.14 | 0.053 |
| Exposure time | 6, 147 | 3.80 | **0.0015** |
| Site | 84, 912 | 1.22 | 0.091 |
| Country × Crop | 42, 912 | 1.95 | **0.00036** |
| Country × Exposure time | 42, 912 | 1.66 | **0.006** |
| Crop × Exposure time | 6, 147 | 1.16 | 0.33 |
| ***Osmia bicornis*** |  |  |  |
| Country | 16, 156 | 4.23 | **<0.0001** |
| Crop | 4, 36 | 2.64 | 0.050 |
| Exposure time | 4, 36 | 0.73 | 0.58 |
| Site | 56, 156 | 1.40 | 0.057 |
| Country × Crop | 16, 156 | 2.58 | **0.0014** |
| Country × Exposure time | 16, 156 | 2.71 | **0.00079** |
| Crop × Exposure time | 4, 36 | 0.50 | 0.74 |

**Supplementary table S19.** Results of the ANCOVA (*P* values) of the load changes between screening occasions T1 and T0 of each of the eight most frequent IPA in the three bee species. Na: *Nosema apis*, Nc: *N. ceranae*. The significance threshold of *P* values was set at α=0.05.

| **Source of variation** | **ABPV** | **BQCV** | **CBPV** | **DWV-A** | **DWV-B** | **SBV** | **Na** | **Nc** |
| --- | --- | --- | --- | --- | --- | --- | --- | --- |
| ***Apis mellifera*** |  |  |  |  |  |  |  |  |
| Country | 0.39 | 0.19 | **<0.001** | **<0.001** | **<0.001** | **<0.001** | **0.035** | **0.016** |
| Crop | 0.41 | 0.44 | 0.85 | 0.52 | 0.11 | 0.66 | 0.55 | 0.49 |
| Exposure time | 0.93 | **<0.001** | 0.34 | 0.76 | 0.60 | 0.13 | 0.84 | 0.20 |
| Site | 0.09 | 0.94 | 0.98 | 0.50 | 0.76 | 0.66 | 0.66 | 0.99 |
| Country × Crop | 0.27 | 0.42 | 0.06 | 0.56 | 0.98 | 0.13 | 0.64 | 0.74 |
| Country × Exposure time | 0.83 | 0.46 | 0.41 | 0.89 | 0.18 | 0.67 | 0.84 | 0.07 |
| Crop × Exposure time | 0.12 | 0.08 | 0.11 | 0.60 | 0.70 | 0.81 | 0.69 | 0.88 |
| ***Bombus terrestris*** |  |  |  |  |  |  |  |  |
| Country | **<0.001** | **<0.001** | 0.06 | 0.07 | **0.031** | **<0.001** |  |  |
| Crop | **0.025** | 0.57 | 0.94 | 0.27 | 0.39 | 0.25 |  |  |
| Exposure time | 0.95 | 0.97 | 0.73 | 0.73 | 0.56 | 0.38 |  |  |
| Site | 0.98 | **0.023** | 0.29 | 0.49 | 0.46 | 0.96 |  |  |
| Country × Crop | 0.48 | 0.08 | 0.99 | 0.49 | **0.006** | 0.37 |  |  |
| Country × Exposure time | 0.18 | 0.65 | **0.030** | 0.20 | 0.66 | 0.32 |  |  |
| Crop × Exposure time | 0.87 | 0.32 | 0.99 | 0.39 | 0.07 | 0.46 |  |  |
| ***Osmia bicornis*** |  |  |  |  |  |  |  |  |
| Country |  | **0.006** |  | 0.14 | **0.002** | **<0.001** |  |  |
| Crop |  | 0.41 |  | 0.96 | **0.005** | 0.13 |  |  |
| Exposure time |  | 0.31 |  | 0.81 | 0.14 | 0.24 |  |  |
| Site |  | 0.07 |  | 0.68 | **0.01** | 0.80 |  |  |
| Country × Crop |  | 0.91 |  | 0.23 | **<0.001** | 0.67 |  |  |
| Country × Exposure time |  | 0.84 |  | 0.56 | **<0.001** | 0.83 |  |  |
| Crop × Exposure time |  | 0.52 |  | 0.22 | 0.77 | 0.97 |  |  |

**Supplementary figure S4.** **Mean IPA loads (in log_10_ (IPA/bee), ± 95% confidence intervals) on the two screening occasions for the more frequently detected IPAs in the three sentinel bees.** The dashed line stands for the mean IPA load at T0 equals to that at T1, *i.e.* no change in mean IPA loads between screening occasions.

**
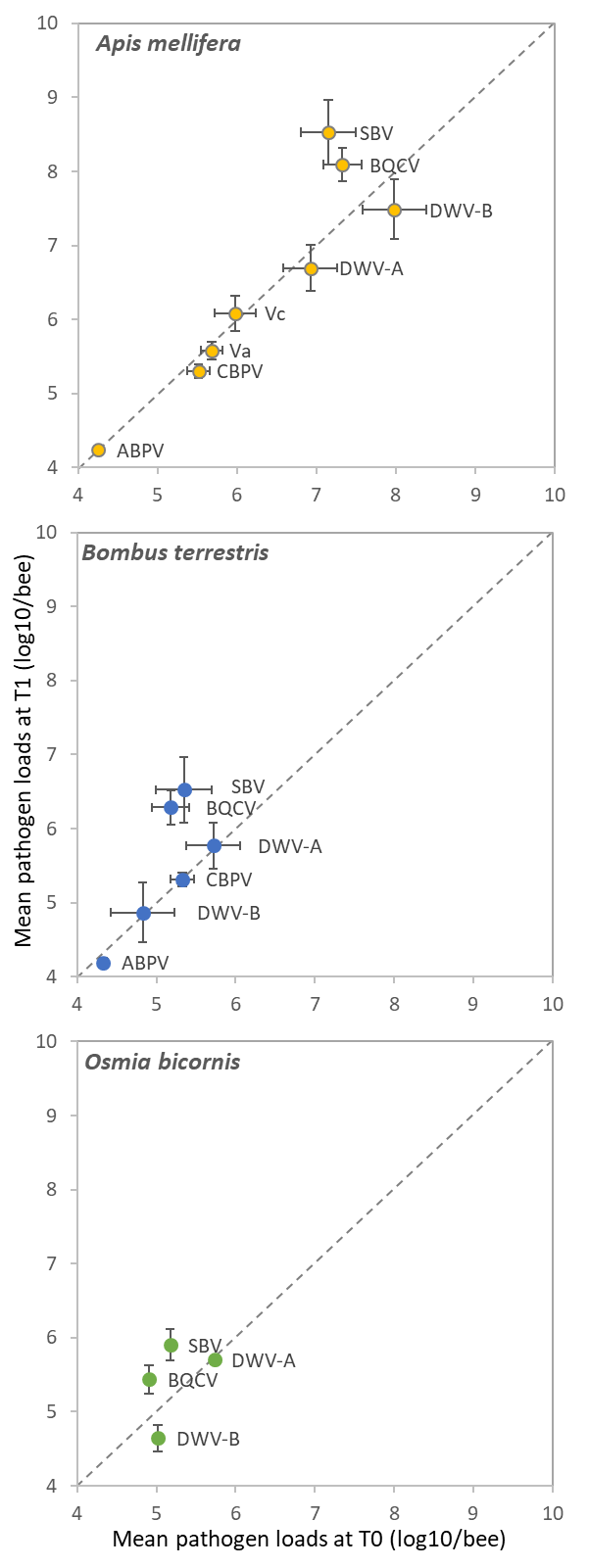
**

**Supplementary figure S5.** IPA loads before field exposure (at screening T0) in *Apis mellifera*. Boxplots indicate the quartiles and median, and the red points are the mean IPA loads. For the statistical analysis of load change during field placement, the non-detected analytical results were replaced by the corresponding limit of detection of the molecular method (see Supplementary table S30).


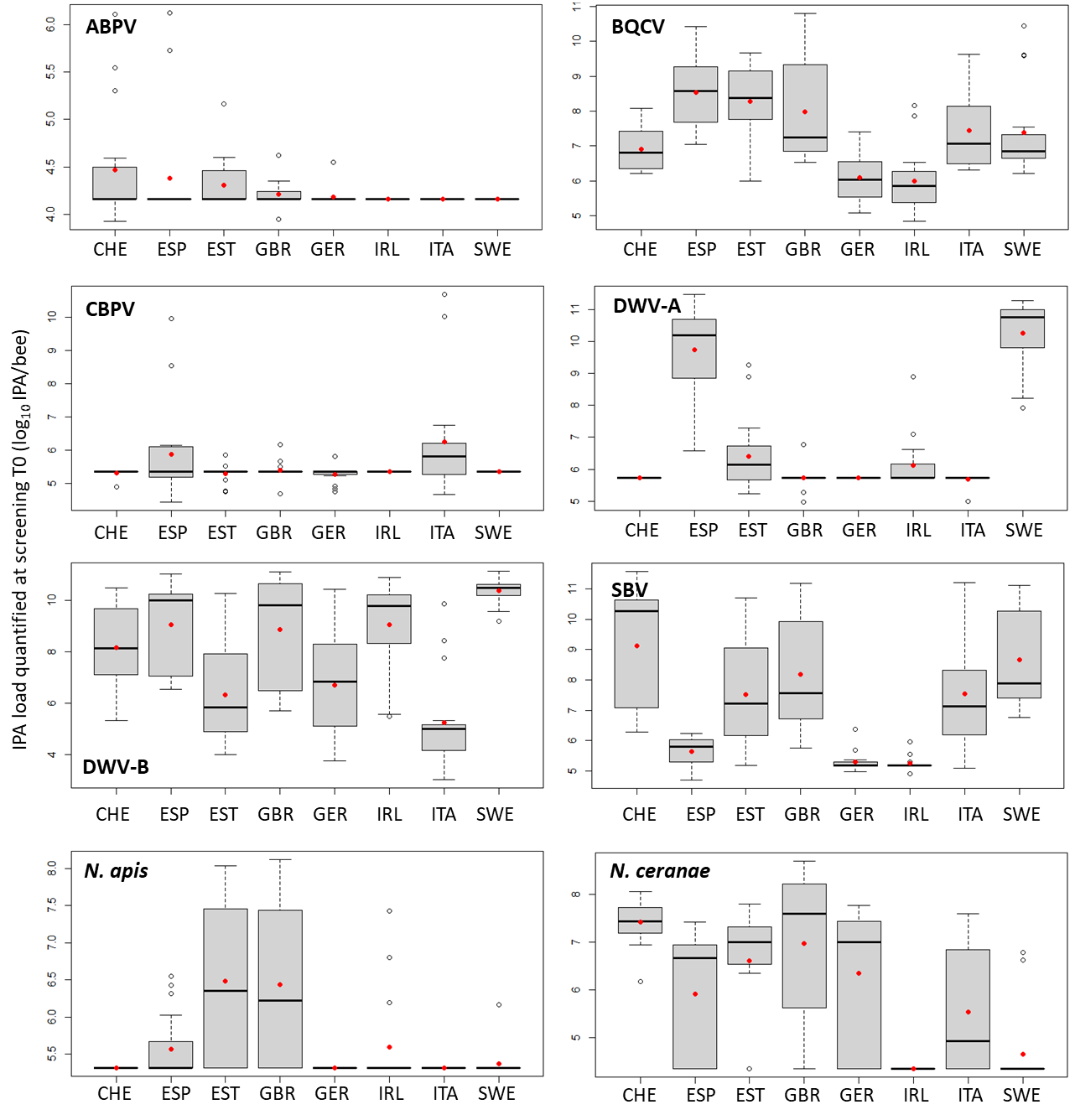


**Supplementary figure S6.** IPA loads quantified at each site in *Apis mellifera* for each country, screening occasion (T0, T1) and focal crop (oilseed rape fields OSR and apple orchards APP). Countries are presented by biogeographical zone: Atlantic area (United Kingdom GBR, Ireland IRL), Mediterranean area (Spain ESP, Italy ITA), Continental area (Switzerland CHE, Germany GER), and Boreal area (Estonia EST, Sweden SWE). Numbers of detected IPAs at each site are indicated above the x axis, missing numbers correspond to non-sampled sites.


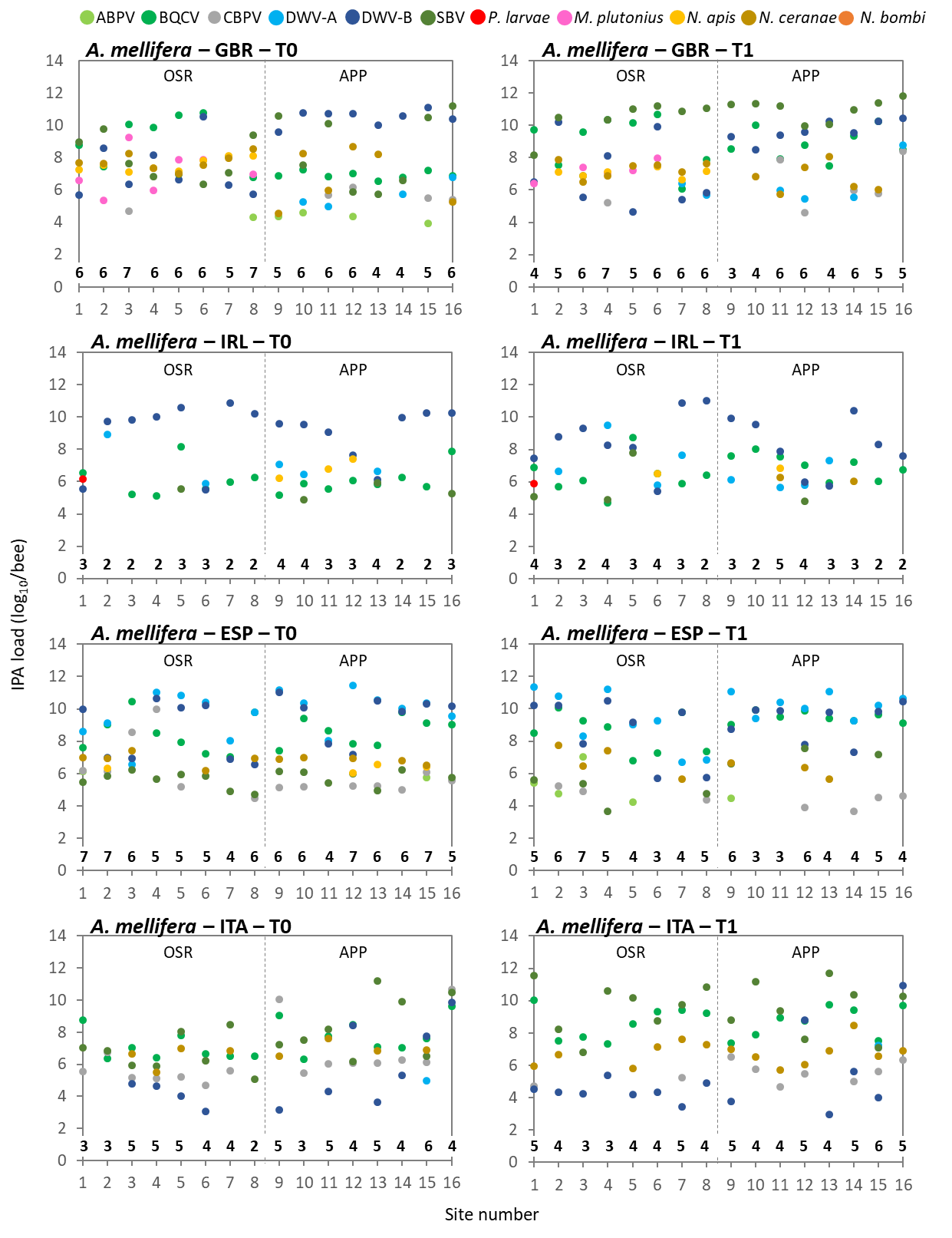


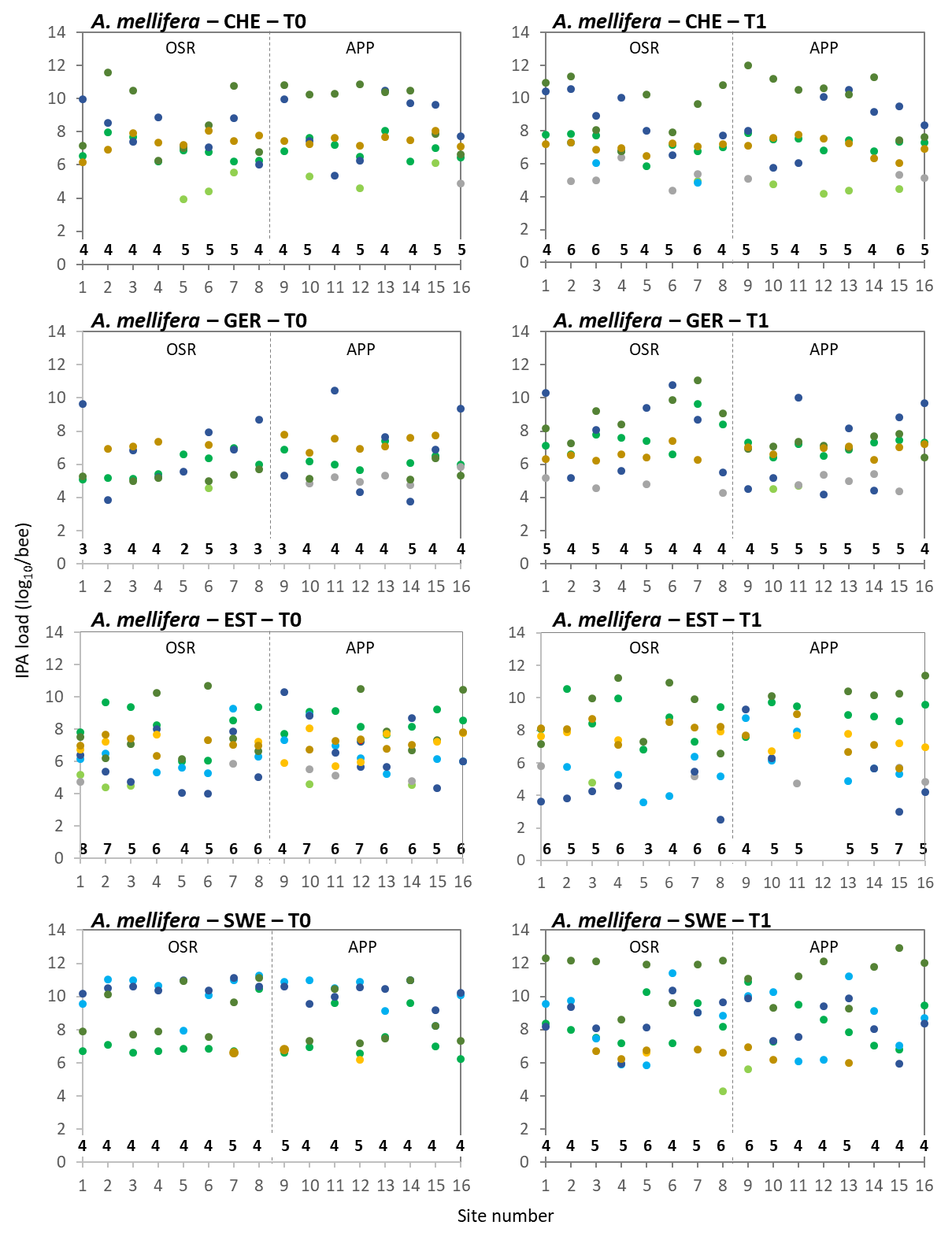


**Supplementary figure S7.** IPA loads before field exposure (at screening T0) in *Bombus terrestris*. Boxplots indicate the quartiles and median, and the red points are the mean IPA loads. For the statistical analysis of load change during field placement, the non-detected analytical results were replaced by the corresponding limit of detection of the molecular method (see Supplementary table S30).

**
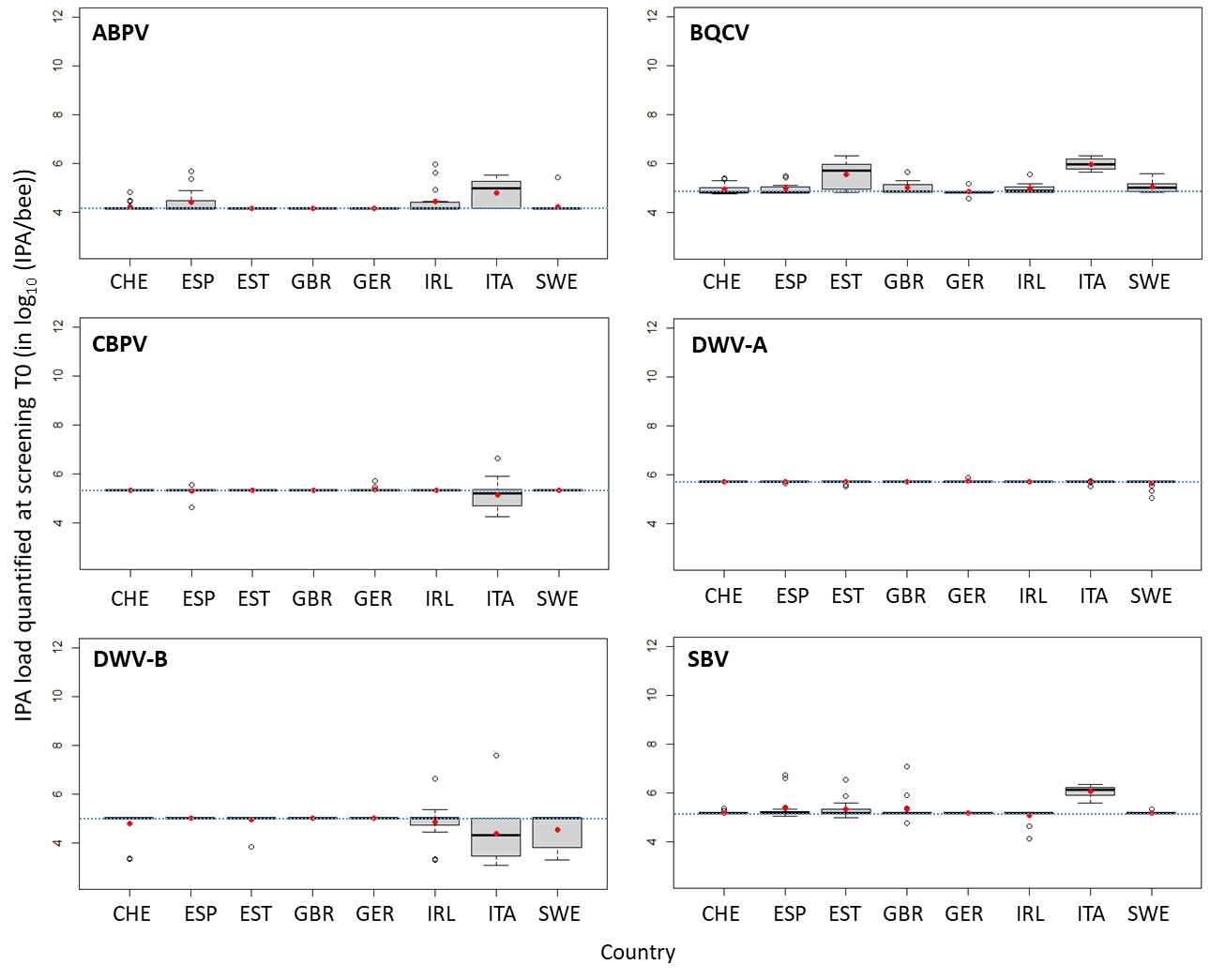
**

**Supplementary figure S8.** IPA loads quantified at each site in *Bombus terrestris* for each country, screening occasion (T0, T1) and focal crop (oilseed rape fields OSR and apple orchards APP). Countries are presented by biogeographical area: Atlantic area (United Kingdom GBR, Ireland IRL), Mediterranean area (Spain ESP, Italy ITA), Continental area (Switzerland CHE, Germany GER), and Boreal area (Estonia EST, Sweden SWE). Numbers of detected IPAs at each site are indicated above the x axis, missing numbers correspond to non-sampled sites.


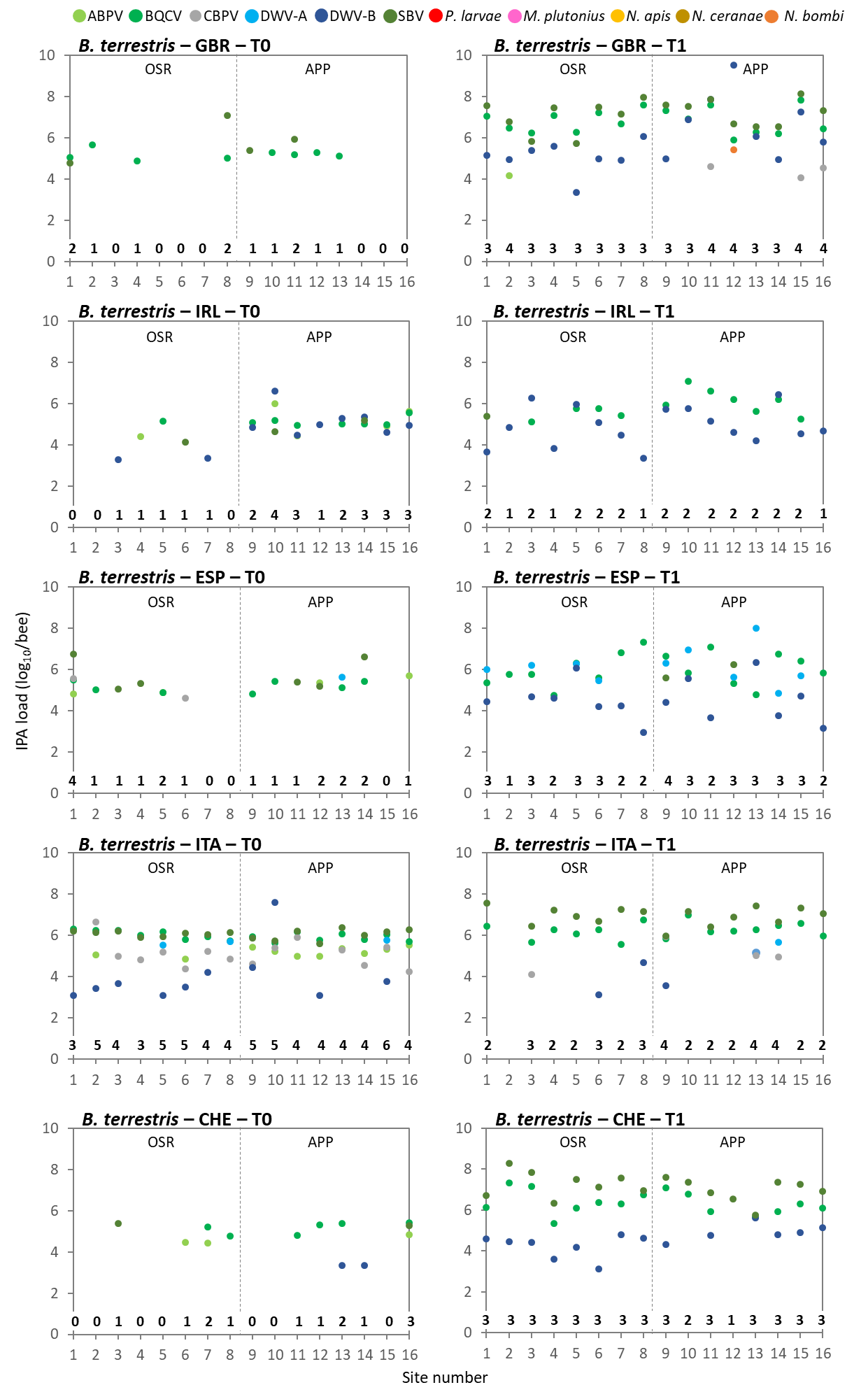


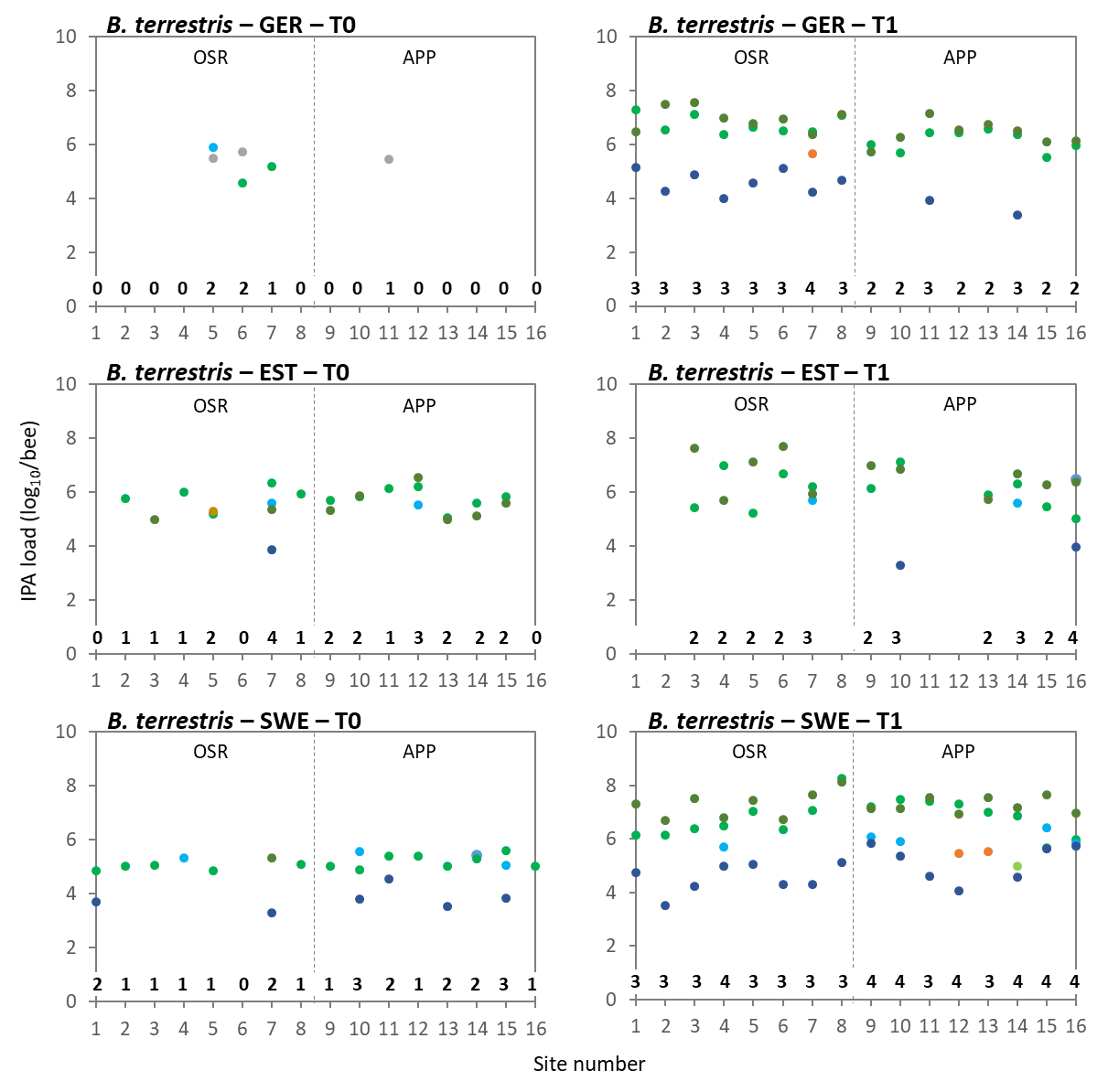


**Supplementary figure S9.** IPA loads quantified at each site at screening T1 in *Osmia bicornis* for each country, and focal crop (oilseed rape fields OSR and apple orchards APP). Countries are Spain ESP, Switzerland CHE, Germany GER, Estonia EST and Sweden SWE. Numbers of detected IPAs at each site are indicated above the x axis, missing numbers correspond to non-sampled sites.

**
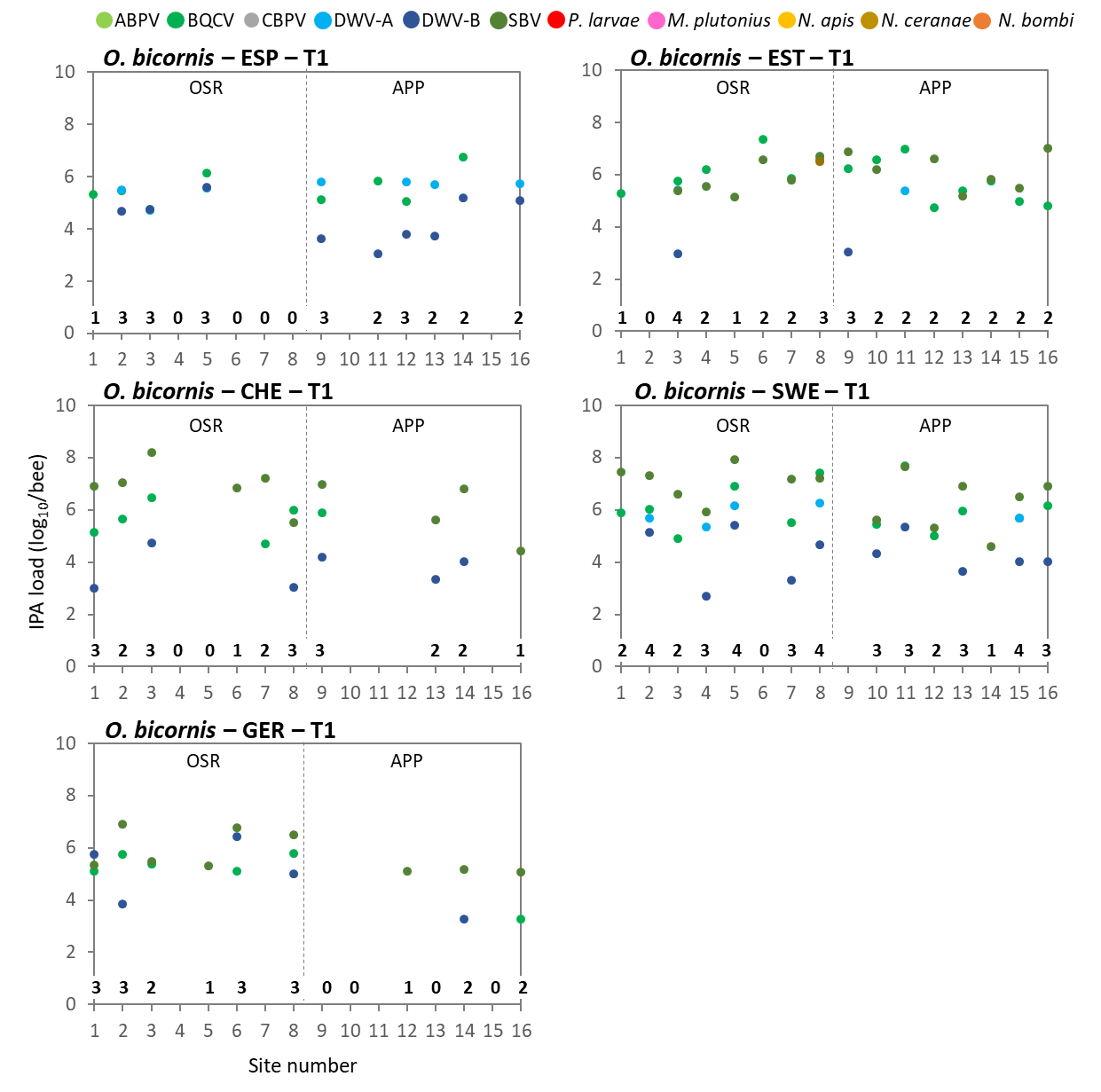
**

**Supplementary table S20. Mean IPA loads after field exposure (T1) on each crop and in each country for the three sentinel bees (± 95% confidence intervals, site numbers between brackets).** Countries: CHE=Switzerland, ESP=Spain, EST=Estonia, GBR=United Kingdom, GER=Germany, IRL=Ireland, ITA=Italy, and SWE=Sweden. Crops: O=oilseed rape, A=apple orchards. The table includes only the more frequently detected IPAs. Highlighted bold values indicate non-overlapping 95% CI.

| **Bee species** | **IPA** | **CHE** | **ESP** | **EST** | **GBR** | **GER** | **IRL** | **ITA** | **SWE** |
| --- | --- | --- | --- | --- | --- | --- | --- | --- | --- |
| *Apis mellifera* | ABPV | O : 4.95 (1)  A : 4.45 ± 0.31 (4) | O : 5.35 ± 1.67 (4)  A : 4.47 (1) | O : 4.77 (1)  *A : NA* | *O : NA*  *A : NA* | *O : NA*  A : 4.60 ± 0.49 (2) | *O : NA*  *A : NA* | *O : NA*  *A : NA* | O : 4.27 (1)  A : 5.62 (1) |
|  | BQCV | O : 7.12 ± 0.54 (8)  A : 7.33 ± 0.30 (8) | O : 8.49 ± 1.00 (8)  A : 9.47 ± 0.27 (8) | O : 8.68 ± 1.04 (8)  A : 8.97 ± 0.67 (7) | O : 9.00 ± 1.33 (8)  A : 8.85 ± 0.79 (8) | O : 7.64 ± 0.82 (8)  A : 7.05 ± 0.33 (8) | O : 6.36 ± 0.95 (8)  A : 7.01 ± 0.60 (8) | O : 8.64 ± 0.82 (8)  A : 8.67 ± 0.78 (8) | O : 8.27 ± 0.91 (8)  A : 8.43 ± 1.18 (8) |
|  | CBPV | O : 5.23 ± 0.87 (5)  A : 5.18 ± 0.25 (3) | O : 4.82 ± 0.80 (3)  A : 4.15 ± 0.64 (4) | O : 5.48 ± 1.33 (2)  A : 5.09 ± 0.96 (3) | O : 5.22 (1)  A : 6.54 ± 1.81 (5) | O : 4.70 ± 0.53 (4)  A : 4.98 ± 0.51 (5) | *O : NA*  *A : NA* | O : 4.96 ± 1.12 (2)  A : 5.62 ± 0.60 (7) | *O : NA*  *A : NA* |
|  | DWV-A | O : 6.09 ± 2.24 (3)  *A : NA* | O : 9.18 ± 1.50 (8)  A : 10.26 ± 0.55 (8) | O : 5.02 ± 1.08 (6)  A : 6.46 ± 1.56 (6) | O : 6.03 ± 1.53 (2)  A : 6.43 ± 2.17 (4) | *O : NA*  *A : NA* | O : 7.40 ± 2.22 (4)  A : 6.22 ± 1.05 (4) | *O : NA*  *A: NA* | O : 8.48 ± 1.58 (8)  A : 8.58 ± 1.59 (8) |
|  | DWV-B | O : 8.88 ± 1.37 (7)  A : 8.43 ± 1.44 (8) | O : 8.65 ± 1.62 (8)  A : 9.22 ± 0.93 (8) | O : 4.06 ± 1.00 (6)  A : 5.70 ± 2.74 (5) | **O : 7.02 ± 1.74 (8)**  **A : 9.65 ± 0.52 (8)** | O : 7.93 ± 1.83 (8)  A : 6.87 ± 2.07 (8) | O : 8.65 ± 1.48 (8)  A : 8.18 ± 1.41 (8) | O : 4.40 ± 0.46 (8)  A : 6.01 ± 3.17 (6) | O : 8.60 ± 1.10 (8)  A : 8.29 ± 1.13 (8) |
|  | SBV | O : 9.46 ± 1.36 (8)  A : 10.10 ± 1.37 (8) | **O : 4.84 ± 1.20 (4)**  **A : 7.09 ± 0.89 (3)** | O : 9.02 ± 1.74 (7)  A : 10.48 ± 0.58 (5) | O : 10.00 ± 1.31 (8)  A : 11.00 ± 0.54 (8) | **O : 9.01 ± 1.10 (7)**  **A : 7.17 ± 0.37 (8)** | O : 5.93 ± 2.97 (3)  A : 4.82 (1) | O : 9.58 ± 1.28 (8)  A : 9.55 ± 1.34 (8) | O : 11.36 ± 1.16 (8)  A : 11.22 ± 1.08 (8) |
|  | *N. apis* | *O : NA*  *A : NA* | *O : NA*  *A : NA* | O : 7.71 ± 0.34 (4)  A : 7.29 ± 0.54 (5) | O : 7.06 ± 0.28 (6)  *A : NA* | *O : NA*  *A : NA* | O : 6.53 (1)  A : 6.84 (1) | *O : NA*  *A : NA* | *O : NA*  *A : NA* |
|  | *N. ceranae* | O : 7.04 ± 0.22 (8)  A : 7.07 ± 0.49 (8) | O : 6.83 ± 1.30 (4)  A : 6.20 ± 0.94 (3) | O : 8.15 ± 0.45 (7)  A : 7.23 ± 1.42 (5) | O : 7.29 ± 0.42 (7)  A : 6.72 ± 0.89 (6) | O : 6.54 ± 0.37 (7)  A : 6.89 ± 0.30 (7) | *O : NA*  A : 6.15 ± 0.53 (2) | O : 6.74 ± 0.74 (6)  A : 6.76 ± 0.67 (8) | O : 6.62 ± 0.27 (5)  A : 6.36 ± 0.91 (3) |
| *Bombus terrestris* | ABPV | *O : NA*  *A : NA* | *O : NA*  *A : NA* | *O : NA*  A : 6.48 (1) | O : 4.16 (1)  *A : NA* | *O : NA*  *A : NA* | *O : NA*  *A : NA* | *O : NA*  A : 5.18 (1) | *O : NA*  A : 4.99 (1) |
|  | BQCV | O : 6.43 ± 0.52 (8)  A : 6.26 ± 0.45 (7) | O : 5.96 ± 0.67 (8)  A : 6.09 ± 0.63 (8) | O : 6.11 ± 0.88 (5)  A : 5.99 ± 0.72 (6) | O : 6.83 ± 0.40 (8)  A : 6.80 ± 0.58 (8) | **O : 6.76 ± 0.29 (8)**  **A : 6.13 ± 0.32 (8)** | O : 5.52 ± 0.42 (4)  A : 6.14 ± 0.54 (7) | O : 6.16 ± 0.38 (7)  A : 6.32 ± 0.30 (8) | O : 6.75 ± 0.28 (8)  A : 6.86 ± 0.55 (8) |
|  | CBPV | *O : NA*  *A : NA* | *O : NA*  *A : NA* | *O : NA*  *A : NA* | *O : NA*  A : 4.41 ± 0.54 (3) | *O : NA*  *A : NA* | *O : NA*  *A : NA* | O : 4.10 (1)  A : 5.32 ± 1.01 (3) | *O : NA*  *A : NA* |
|  | DWV-A | *O : NA*  *A : NA* | O : 5.99 ± 0.52 (4)  A : 6.25 ± 1.12 (6) | O : 5.70 (1)  A : 5.58 (1) | *O : NA*  *A : NA* | *O : NA*  *A : NA* | *O : NA*  *A : NA* | *O : NA*  A : 5.66 (1) | O : 5.71 (1)  A : 6.06 ± 0.36 (4) |
|  | DWV-B | O : 4.22 ± 0.47 (8)  A : 4.92 ± 0.44 (6) | O : 4.47 ± 0.82 (7)  A : 4.52 ± 1.01 (7) | *O : NA*  A : 3.6 ± 1.39 (2) | O : 5.05 ± 0.64 (8)  A : 6.66 ± 1.27 (8) | O : 4.61 ± 0.35 (8)  A : 3.67 ± 1.19 (2) | O : 4.69 ± 0.87 (8)  A : 5.13 ± 0.63 (8) | O : 3.91 ± 3.36 (2)  A : 3.56 (1) | O : 4.54 ± 0.45 (8)  A : 5.11 ± 0.62 (7) |
|  | SBV | O : 7.28 ± 0.52 (8)  A : 6.95 ± 0.48 (8) | *O : NA*  A : 5.92 ± 1.42 (2) | O : 6.83 ± 1.08 (5)  A : 6.49 ± 0.46 (6) | O : 6.99 ± 0.67 (8)  A : 7.28 ± 0.51 (8) | O : 6.97 ± 0.35 (8)  A : 6.40 ± 0.36 (8) | O : 5.39 (1)  *A : NA* | O : 7.05 ± 0.34 (7)  A : 6.87 ± 0.40 (8) | O : 7.29 ± 0.42 (8)  A : 7.27 ± 0.23 (8) |
| *Osmia bicornis* | BQCV | O : 5.88 (1)  A : 5.59 ± 0.80 (5) | O : 5.68 ± 1.09 (4)  A : 5.42 ± 0.80 (4) | O : 5.68 ± 0.69 (8)  A : 6.16 ± 0.72 (6) |  | *O : NA*  A : 5.42 ± 0.37 (5) |  |  | O : 5.99 ± 0.92 (6)  A : 6.10 ± 0.91 (6) |
|  | DWV-A | *O : NA*  *A : NA* | O : 5.75 ± 0.06 (4)  A : 5.25 ± 0.84 (3) | *O : NA*  *A : NA* |  | *O : NA*  *A : NA* |  |  | O : 5.67 (1)  A : 5.86 ± 0.60 (4) |
|  | DWV-B | O : 3.84 ± 0.83 (3)  A : 3.60 ± 1.82 (3) | O : 4.08 ± 0.86 (6)  A : 5.00 ± 0.92 (3) | *O : NA*  *A : NA* |  | O : 3.26 (1)  A : 5.25 ± 1.52 (4) |  |  | O : 4.27 ± 0.75 (5)  A : 4.24 ± 1.37 (5) |
|  | SBV | O : 5.96 ± 1.64 (4)  A : 6.94 ± 0.85 (6) | *O : NA*  *A : NA* | O : 6.16 ± 0.63 (7)  A : 5.85 ± 0.63 (6) |  | **O : 5.11 ± 0.10 (3)**  **A : 6.05 ± 0.74 (6)** |  |  | O : 6.20 ± 0.96 (7)  A : 7.08 ± 0.58 (7) |

**Supplementary figure S10. Change in IPA loads between T1 and T0 screening occasions (quartiles, median and mean as red points) on each focal crop (A=apple orchards, O=oil seed rape fields) and in each country in *Apis mellifera*** (CHE=Switzerland, ESP=Spain, EST=Estonia, GBR=United Kingdom, GER=Germany, IRL=Ireland, ITA=Italy, and SWE=Sweden). For this analysis to be possible, the non-detected analytical results were replaced by the corresponding limit of detection of the molecular method (see Supplementary table S30).


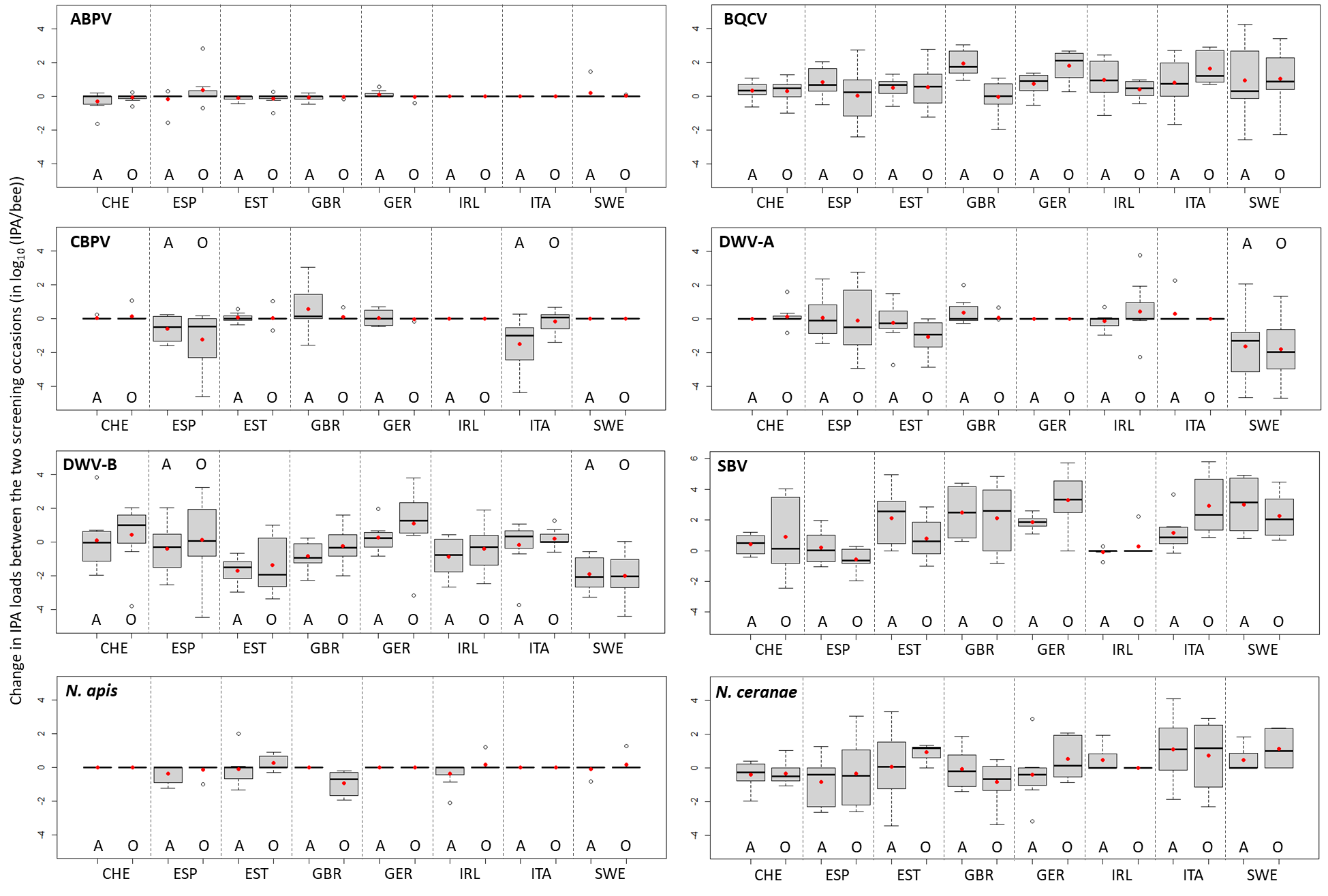


**Supplementary figure S11. Change in IPA loads between T1 and T0 screening occasions (quartiles, median and mean as red points) on each focal crop (A=apple orchards, O=oil seed rape fields) and in each country in *Bombus terrestris*** (CHE=Switzerland, ESP=Spain, EST=Estonia, GBR=United Kingdom, GER=Germany, IRL=Ireland, ITA=Italy, and SWE=Sweden). For this analysis to be possible, the non-detected analytical results were replaced by the corresponding limit of detection of the molecular method (Supplementary table S30).


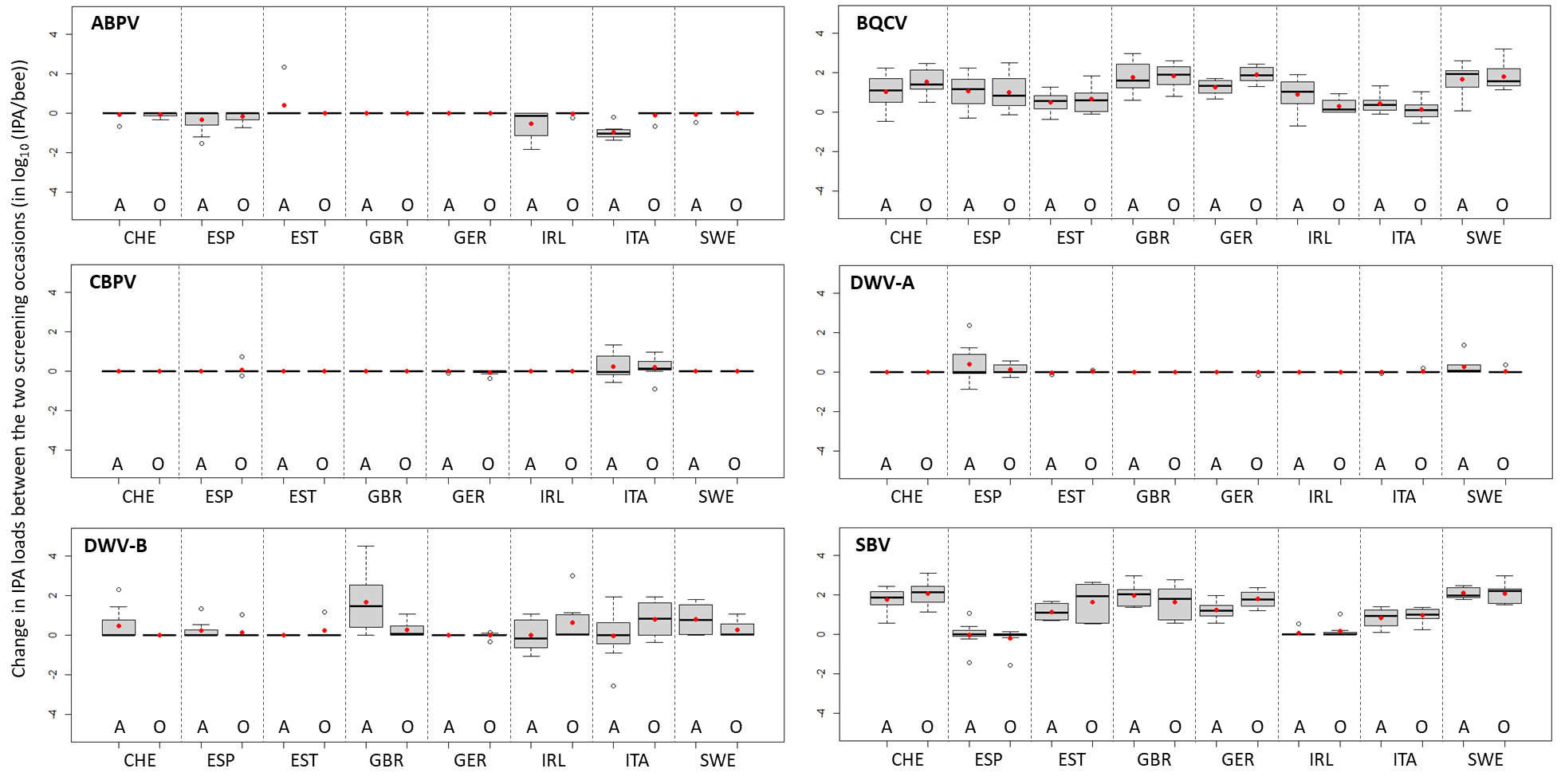


**Supplementary figure S12. Change in IPA loads between T1 and T0 screening occasions (quartiles, median and mean as red points) on each focal crop (A=apple orchards, O=oil seed rape fields) and in each country in *Osmia bicornis*** (CHE=Switzerland, ESP=Spain, EST=Estonia, GER=Germany, and SWE=Sweden). For this analysis to be possible, the non-detected analytical results were replaced by the corresponding limit of detection of the molecular method (see Supplementary table S30).


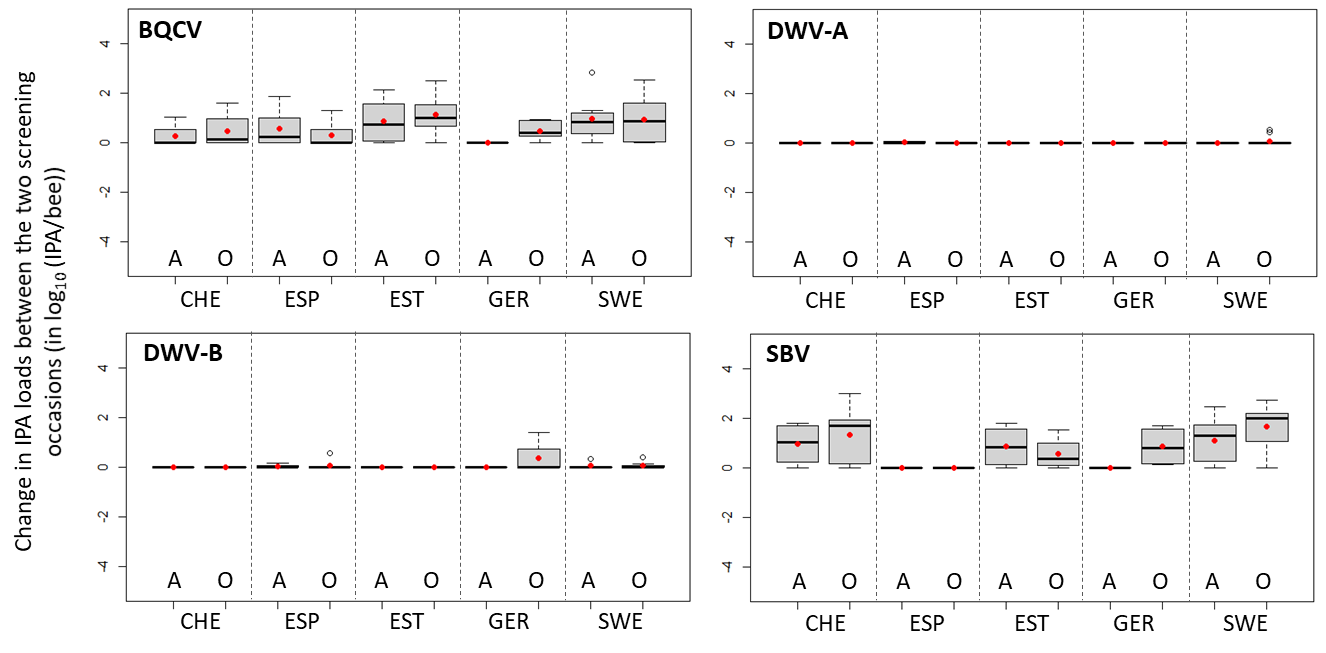


**Supplementary table S21.** **Relationships between the loads of BQCV and of the microsporidia *Nosema apis* and *Nosema ceranae* in *Apis mellifera* after field exposure (T1).** The table below summarizes the results of the Pearson’s correlation test.

| **Correlation** | ***r*** | ***df*** | ***t*** | ***P*** |
| --- | --- | --- | --- | --- |
| BQCV – *N. apis* | 0.40 | 16 | 1.72 | 0.10 |
| BQCV – *N. ceranae* | 0.04 | 84 | 0.41 | 0.68 |

**Supplementary figure S13.** **Relationships between the loads of BQCV and of the microsporidia *Nosema apis* in *Apis mellifera* after field exposure (T1).** The plots show data of all the countries (left), and for each country separately with different colours (right).


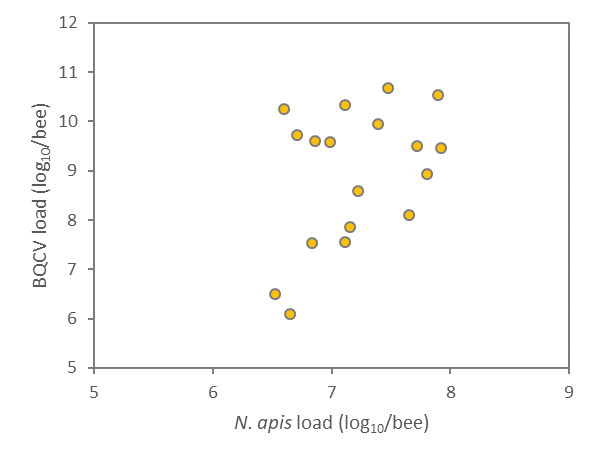

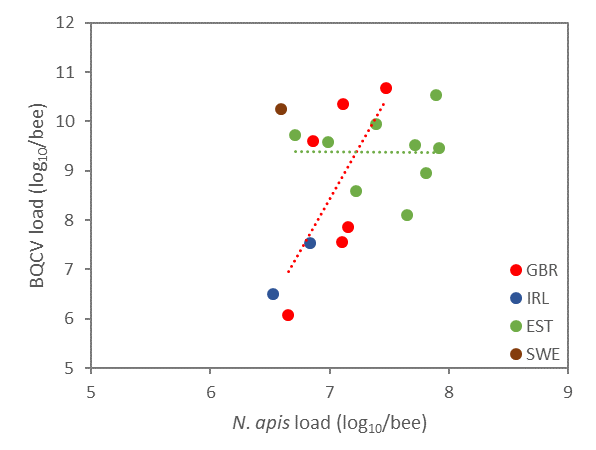


**Supplementary figure S14.** **Relationships between the loads of BQCV and of the microsporidia *Nosema ceranae* in *Apis mellifera* after field exposure (T1).** The plots show data of all the countries (left), and for each country separately with different colours (right).


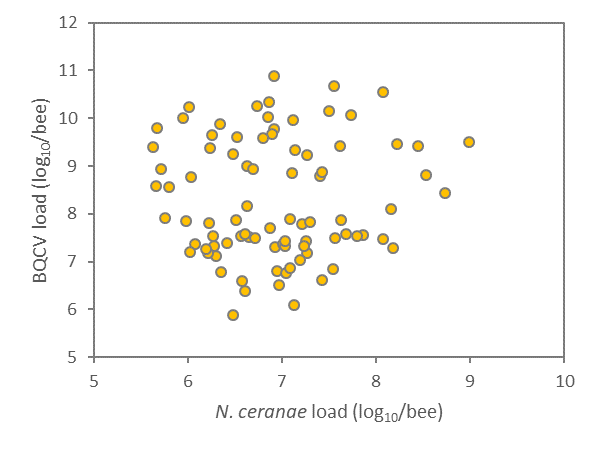

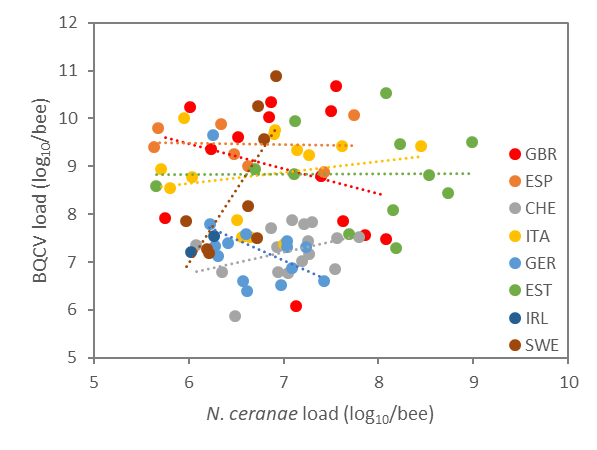


**Supplementary table S22.** **Relationships between the loads of the viruses DWV-A and DWV-B, and those of the microsporidia *Nosema apis* and *Nosema ceranae* in A*pis mellifera* after field exposure (T1)**. Results of the statistical analysis with Pearson’s correlation test. Similar results were obtained with non-parametric Spearman’s correlation test.

| **Correlation** | ***r*** | ***df*** | ***t*** | ***P*** |
| --- | --- | --- | --- | --- |
| DWV-A – *N. apis* | -0.11 | 10 | -0.34 | 0.74 |
| DWV-A – *N. ceranae* | -0.20 | 33 | -1.17 | 0.25 |
| DWV-B – *N. apis* | -0.38 | 14 | -1.52 | 0.15 |
| DWV-B – *N. ceranae* | -0.18 | 78 | -1.62 | 0.11 |

**Supplementary figure S15.** Relationships at T1 between the loads of the viruses DWV-A (A,B) and DWV-B (C,D), and those of the two honey bee microsporidia, *Nosema apis* (A,C) and *Nosema ceranae* (B,D). noV–noDW = no mites or wing deformities, noV–DW = no mite but bee deformities, V–noDW = mites without bee deformities, V–DW = mites and bee deformities.


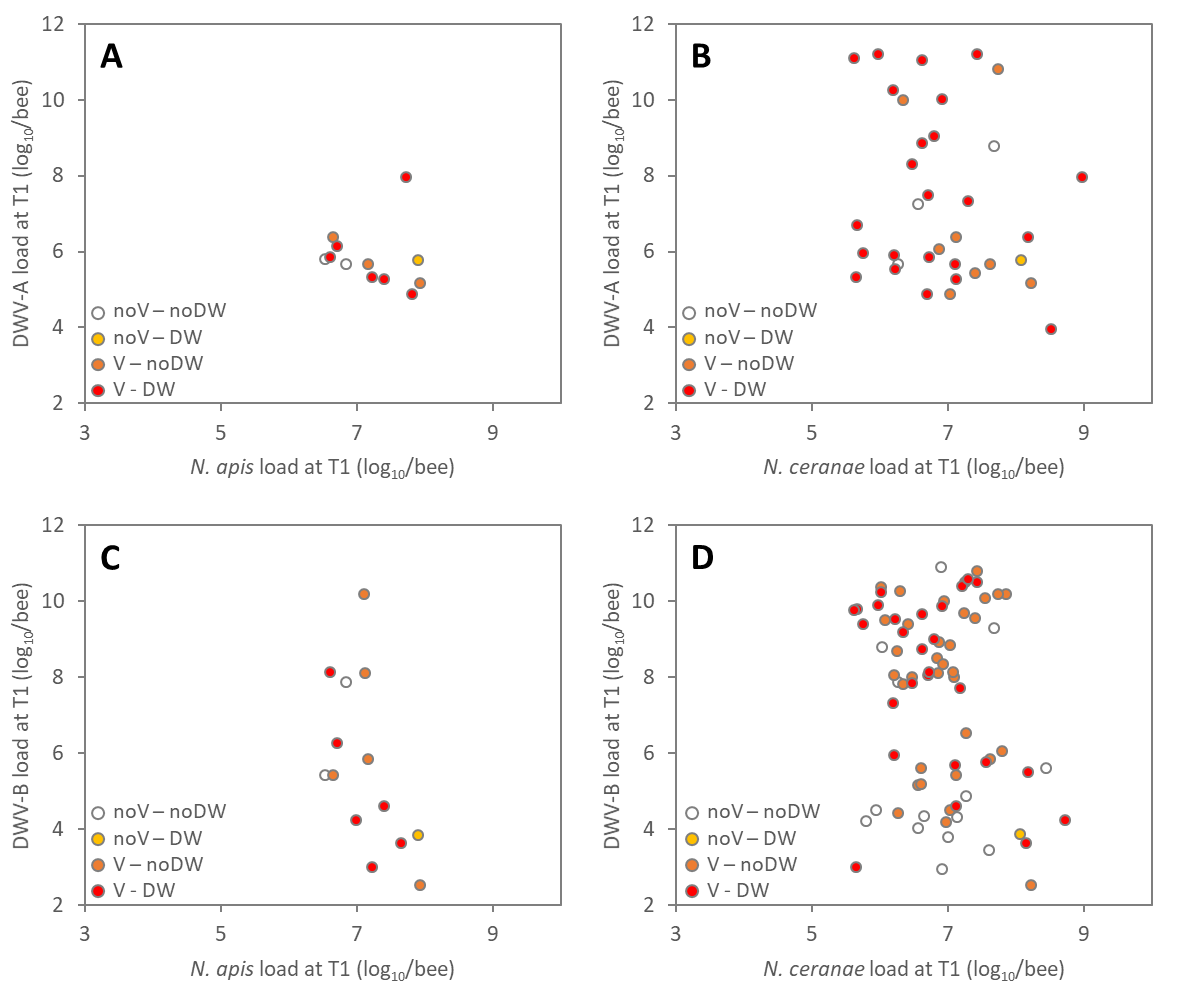


**Supplementary table S23.** Results of the statistical analysis with Pearson’s test of correlation testing the relationship between loads of DWV-A and DWV-B after field exposure (T1) in each sentinel bee species.

| **Sentinel bee** | ***r*** | ***df*** | ***t*** | ***P*** |
| --- | --- | --- | --- | --- |
| *Apis mellifera* | 0.57 | 55 | 5.13 | **<0.0001** |
| *Bombus terrestris* | 0.70 | 12 | 3.44 | **0.005** |
| *Osmia bicornis* | 0.21 | 11 | 0.71 | 0.49 |

**Supplementary figure S16.** Relationship between DWV-A and DWV-B loads in the three sentinel bees after field exposure (T1). For *Apis mellifera* and *Bombus terrestris*, orange points indicate the observation of wing deformities in the colonies, while white points indicate sites without deformities. Wing deformities were not searched for in *Osmia bicornis*. Significant relationships with Pearson’s correlation test are indicated with regression lines.


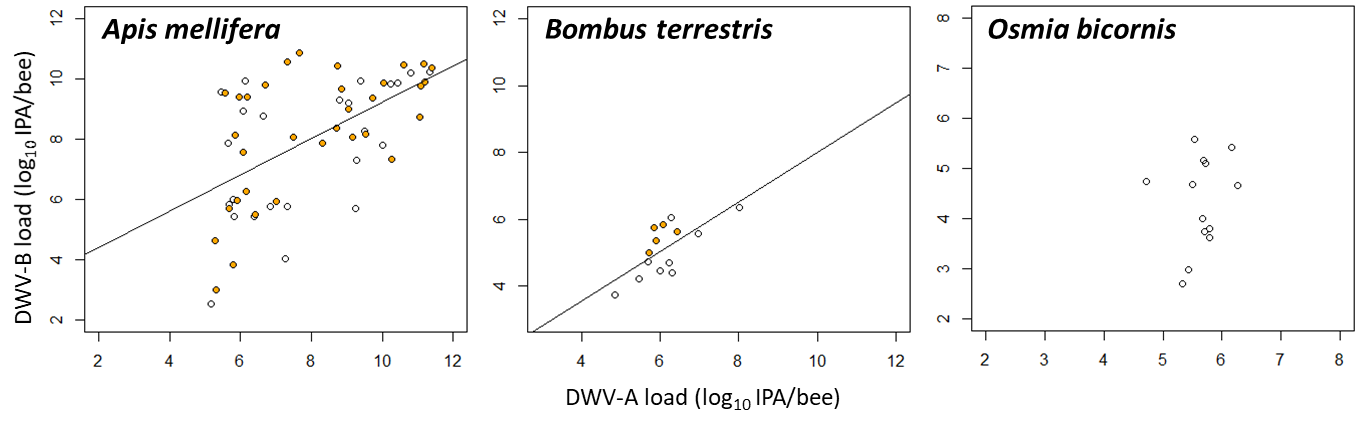


**Supplementary figure S17.** Relationships in *Apis mellifera* between the loads of DWV-A and DWV-B, and the detection frequencies and mean loads of *Varroa destructor* after field exposure (T1). Left plot: relationship between the loads of the two viruses, with red points indicating *V. destructor* presence. Middle and right plots: relationship between the loads of each virus and those of *V. destructor*, with orange points indicating the observation of wing deformities. Significant relationships with Pearson’s correlation test are indicated with regression lines.


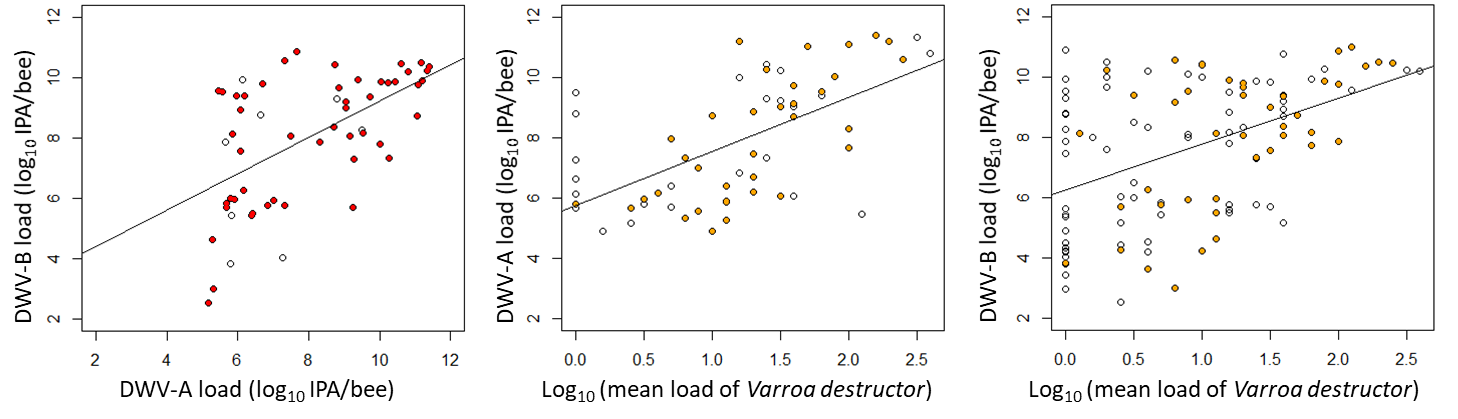


**Supplementary table S24.** Results of the statistical analysis with Pearson’s test of correlation between loads of either DWV-A and DWV-B and loads of *Varroa destructor* mites in *Apis mellifera*.

| **DWV genotype** | ***r*** | ***df*** | ***t*** | ***P*** |
| --- | --- | --- | --- | --- |
| DWV-A | 0.62 | 58 | 6.0 | **<0.0001** |
| DWV-B | 0.46 | 113 | 5.56 | **<0.0001** |

**Supplementary table S25.** **Relationships between IPA loads of co-located sentinel bee species.** Results of the correlation tests (*r* and *P* values; Pearson’s or Spearman’s depending on data distribution and sample size *N*) between loads at screening T0 for both bee species of the pair, the loads at screening T0 for one species and the load change (excluding the zeros) for the other species, and the loads at screening T1 for both bee species. A=*Apis mellifera*, B*=Bombus terrestris*, O=*Osmia bicornis*.

| **IPA** | **Species pair** | | **Screening** | | ***r*** | ***N*** | ***P*** |
| --- | --- | --- | --- | --- | --- | --- | --- |
| BQCV | A – B | | T0 – T0 | **0.28** | | 72 | **0.016** |
|  |  |  | T0 – load change | -0.03 | | 118 | 0.71 |
|  |  |  | load change – T0 | -0.09 | | 71 | 0.47 |
|  |  |  | T1 – T1 | **0.20** | | 116 | **0.035** |
|  | A – O | | T0 – load change | **0.42** | | 41 | **<0.001** |
|  |  |  | T1 – T1 | **0.30** | | 45 | **0.048** |
|  | B – O | | T0 – load change | 0.22 | | 27 | 0.28 |
|  |  |  | T1 – T1 | **0.48** | | 42 | **0.001** |
| CBPV | A – B | | T0 – T0 | -0.29 | | 15 | 0.30 |
|  |  |  | T0 – load change | 0.17 | | 14 | 0.55 |
|  |  |  | load change – T0 | 0.21 | | 15 | 0.46 |
|  |  |  | T1 – T1 | 0.12 | | 5 | 0.85 |
| DWV-A | A – B | | T0 – T0 | -0.22 | | 7 | 0.64 |
|  |  |  | T0 – load change | 0.05 | | 17 | 0.85 |
|  |  |  | load change – T0 | 0.71 | | 6 | 0.14 |
|  |  |  | T1 – T1 | 0.31 | | 17 | 0.22 |
|  | A – O | | T0 – load change | -0.40 | | 4 | 0.75 |
|  |  |  | T1 – T1 | 0.07 | | 13 | 0.82 |
|  | B – O | | T0 – load change | NA | | 0 | NA |
|  |  |  | T1 – T1 | 0.20 | | 7 | 0.67 |
| DWV-B | A – B | | T0 – T0 | 0.06 | | 25 | 0.76 |
|  |  |  | T0 – load change | **0.35** | | 48 | **0.014** |
|  |  |  | load change – T0 | 0.02 | | 25 | 0.94 |
|  |  |  | T1 – T1 | **0.30** | | 89 | **0.004** |
|  | A – O | | T0 – load change | -0.60 | | 8 | 0.13 |
|  |  |  | T1 – T1 | 0.22 | | 33 | 0.21 |
|  | B – O | | T0 – load change | NA | | 2 | NA |
|  |  |  | T1 – T1 | 0.12 | | 28 | 0.54 |
| SBV | A – B | | T0 – T0 | 0.23 | | 37 | 0.18 |
|  |  |  | T0 – load change | **0.40** | | 90 | **<0.001** |
|  |  |  | load change – T0 | 0.10 | | 36 | 0.56 |
|  |  |  | T1 – T1 | **0.45** | | 91 | **<0.001** |
|  | A – O | | T0 – load change | **0.54** | | 35 | **<0.001** |
|  |  |  | T1 – T1 | **0.36** | | 43 | **0.017** |
|  | | B – O | T0 – load change | 0.13 | | 9 | 0.73 |
|  | |  | T1 – T1 | **0.57** | | 44 | **<0.001** |

**Supplementary table S26.** **Relationships between IPA loads of co-located sentinel bee species after removing differences in loads across countries.** Results of the correlation tests as explained in Supplementary table S27. Load differences across countries were taken into account by centring and standardizing the loads within each country with the following formula: (load – mean country load) / standard deviation. A=*Apis mellifera*, B*=Bombus terrestris*, O=*Osmia bicornis*.

| **IPA** | **Species pair** | | **Screening** | | ***r*** | ***N*** | ***P*** |
| --- | --- | --- | --- | --- | --- | --- | --- |
| BQCV | A – B | | T0 – T0 | **0.28** | | 72 | **0.022** |
|  |  |  | T0 – load change | -0.04 | | 118 | 0.70 |
|  |  |  | load change – T0 | **-0.25** | | 71 | **0.037** |
|  |  |  | T1 – T1 | **0.25** | | 115 | **0.006** |
|  | A – O | | T0 – load change | 0.31 | | 41 | 0.051 |
|  |  |  | T1 – T1 | 0.18 | | 45 | 0.25 |
|  | B – O | | T0 – load change | 0.19 | | 27 | 0.35 |
|  |  |  | T1 – T1 | **0.58** | | 42 | **<0.001** |
| CBPV | A – B | | T0 – T0 | -0.27 | | 15 | 0.33 |
|  |  |  | T0 – load change | **0.57** | | 14 | **0.033** |
|  |  |  | load change – T0 | 0.24 | | 15 | 0.38 |
|  |  |  | T1 – T1 | 0.8 | | 5 | 0.13 |
| DWV-A | A – B | | T0 – T0 | 0.9 | | 5 | 0.08 |
|  |  |  | T0 – load change | -0.13 | | 16 | 0.63 |
|  |  |  | load change – T0 | -0.23 | | 4 | 0.77 |
|  |  |  | T1 – T1 | 0.22 | | 17 | 0.39 |
|  | A – O | | T0 – load change | 0 | | 4 | 1 |
|  |  |  | T1 – T1 | 0.31 | | 13 | 0.31 |
|  | B – O | | T0 – load change | NA | | 0 | NA |
|  |  |  | T1 – T1 | 0.32 | | 7 | 0.50 |
| DWV-B | A – B | | T0 – T0 | -0.33 | | 24 | 0.11 |
|  |  |  | T0 – load change | 0.24 | | 47 | 0.11 |
|  |  |  | load change – T0 | -0.07 | | 24 | 0.75 |
|  |  |  | T1 – T1 | **0.22** | | 89 | **0.037** |
|  | A – O | | T0 – load change | -0.03 | | 8 | 0.94 |
|  |  |  | T1 – T1 | 0.26 | | 32 | 0.16 |
|  | B – O | | T0 – load change | NA | | 1 | NA |
|  |  |  | T1 – T1 | 0.06 | | 27 | 0.78 |
| SBV | A – B | | T0 – T0 | 0.33 | | 35 | 0.051 |
|  |  |  | T0 – load change | 0.15 | | 90 | 0.15 |
|  |  |  | load change – T0 | -0.12 | | 35 | 0.49 |
|  |  |  | T1 – T1 | **0.30** | | 90 | **0.039** |
|  | A – O | | T0 – load change | **0.51** | | 35 | **0.002** |
|  |  |  | T1 – T1 | 0.22 | | 43 | 0.15 |
|  | | B – O | T0 – load change | 0.52 | | 8 | 0.18 |
|  | |  | T1 – T1 | **0.46** | | 44 | **0.002** |

**Supplementary figure S18. Location of field sites for the placement of honey bee colonies, and bumble bee and mason bee nests.** The yellow-filled circles indicate sites near oilseed rape crops. The green-filled circles indicate sites near apple orchards. The geographical proximity of the sites near both crops is represented by yellow and green semi-circles. Biogeographic zones are represented by a blue ring for the Atlantic zone, orange for Mediterranean zone, green for Continental zone and white for Boreal zone. For more details on the location of the sites, see Hodge *et al.* 2022 [reference 32 of the article].

**
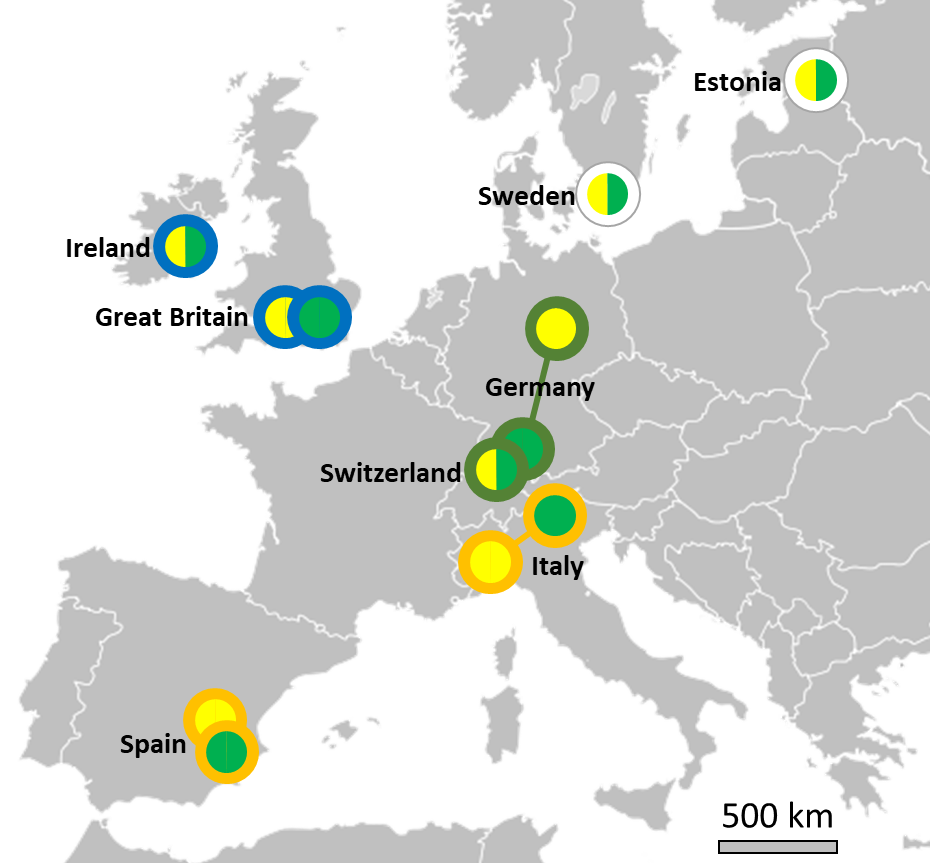
**

**Supplementary table S27.** Details of the five subspecies of the *A. mellifera* colonies deployed (CHE=Switzerland, ESP=Spain, EST=Estonia, GBR=United Kingdom, GER=Germany, IRL=Ireland, ITA=Italy, and SWE=Sweden ; O=oilseed rape, A=apple).

| Subspecies | *buckfast* | *carnica* | *iberiensis* | *ligustica* | *mellifera* | mixed |
| --- | --- | --- | --- | --- | --- | --- |
| Countries (Crop) | GBR (O) | CHE (O, A)  GER (A) | ESP (O, A) | EST (O, A) ITA (O, A) | IRL (O, A) GER (O) | SWE (O, A)  GBR (A) |
| Site number | 8 | 24 | 16 | 32 | 24 | 24 |

**Supplementary figure S19. Diagram of the deployment and sampling of the three sentinel bees.** Honey bee *Apis mellifera*, bumble bee *Bombus terrestris*, and mason bee *Osmia bicornis*, were sampled for the 11 IPAs at screening T0 (prior to bee’s deployment) and screening T1 (after bees’ exposure to the field).


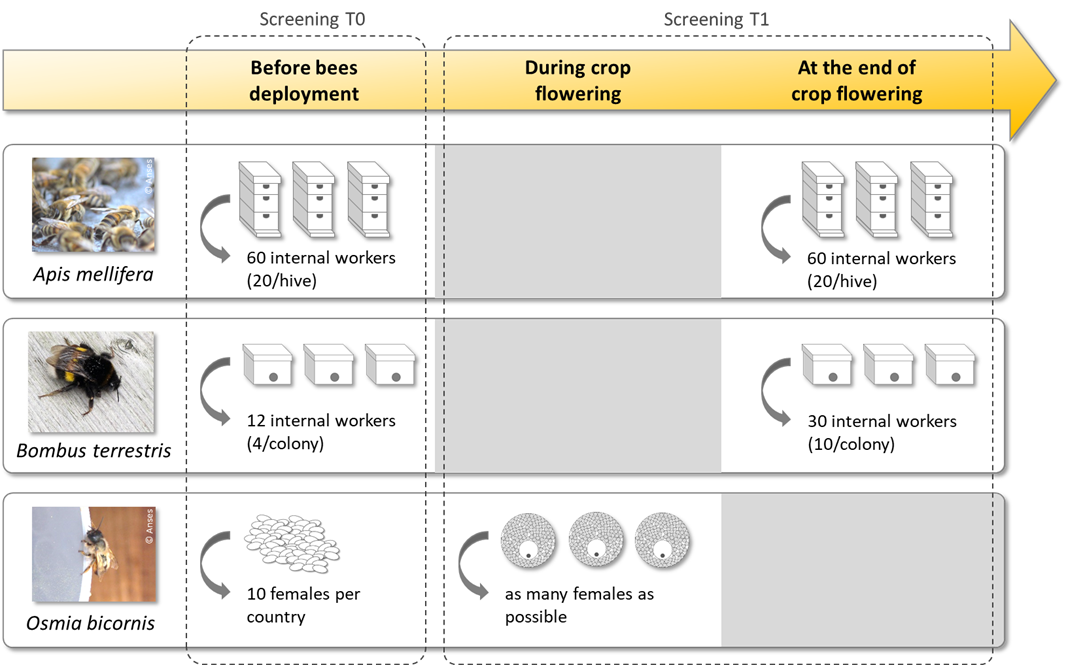


**Supplementary table S28. Size of *O. bicornis* pooled samples.** Numbers of *Osmia bicornis* females collected when returning to the trap nests during the crop flowering period, at each oilseed rape sites (OSR, sites 1 to 8) and apple orchard sites (APP, sites 9 to 16). Numbers varied widely from one site to another, due to variation in the efficiency at which bees settled in the trap nests. Sites with *na* (not available) are sites where no solitary bees were collected.

| **Country** | **OSR sites** | **APP sites** |
| --- | --- | --- |
| Estonia | 14 – *na* – 39 – 43 – 13 – 42 – 32 – 37 | 21 – 16 – 25 – 25 – 26 – 14 – 32 – 30 |
| Germany | 21 – 15 – 2 – *na* – 2 – 17 – *na* – 1 | 5 – 11 – *na* – 8 – 1 – 14 – 4 – 18 |
| Spain | 13 – 8 – 13 – 9 – 3 – 3 – 9 – 1 | 2 – *na* – 9 – 22 – 2 – 2 – *na* – 1 |
| Sweden | 18 – 14 – 5 – 8 – 16 – 2 – 36 – 13 | *na* – 9 - 32 – 10 – 21 – 7 – 13 – 11 |
| Switzerland | 7 – 19 – 5 – 3 – 9 – 26 – 29 – 5 | 16 – *na* – *na* – *na* – 15 – 28 – *na* – 25 |

**Supplementary figure S20. Accuracy profiles and systematic bias of the quantification method for each IPA in each sentinel bees.** For each IPA screened in each sentinel bee, the method accuracy profile was built on 3 to 4 loads of the specific plasmid spiked in a bee homogenate devoid of the screened IPA. Loads ranged from the detection limit of the method (4 or 5 log_10_ copies/bee) up to 10 log_10_ copies/bee (3 independent sets of loads, each load being purified once or twice). Analytical results were compared with the theoretical spiked plasmid loads.

Accuracy profiles (blue lines in the figures below) were built with the mean bias (trueness error) between the mean analytical results and the theoretical expected loads, and the data reproducibility (SD_R_, precision error)^[1]^. Profiles were interpreted with the upper and lower tolerance limits (95% confidence intervals, red lines), and the upper and lower acceptable limits set at ± 1 log_10_ (blue dotted lines). In the molecular method characterisation, the lower and upper limits of quantification corresponded to the lowest and highest plasmid loads quantified with an acceptable accuracy falling within ± 1 log_10_ copies/bee. The systematic method biases (trueness error) are indicated in Supplementary figure S21. Accuracy profiles and systematic method biases for the quantification of *Nosema apis*, *Nosema ceranae*, and *Nosema bombi* have already been presented.^[2]^

Systematic method bias (in log_10_ copies/bee) for IPA quantification in each sentinel bee.

|  | ABPV | BQCV | CBPV | DWV-A | DWV-B | SBV | *P. larvae* | *M. plutonius* |
| --- | --- | --- | --- | --- | --- | --- | --- | --- |
| *A. mellifera* | 0.38 | 0.07 | -0.13 | -0.29 | -0.51 | 0.03 | 0.52 | 0.33 |
| *B. terrestris* | 0.34 | -0.09 | -0.10 | -0.17 | -0.46 | 0.36 | 0.60 | 0.31 |
| *O. bicornis* | -0.74 | -1.20 | -1.26 | -1.17 | -1.78 | -0.97 | -0.95 | -1.08 |

- ABPV


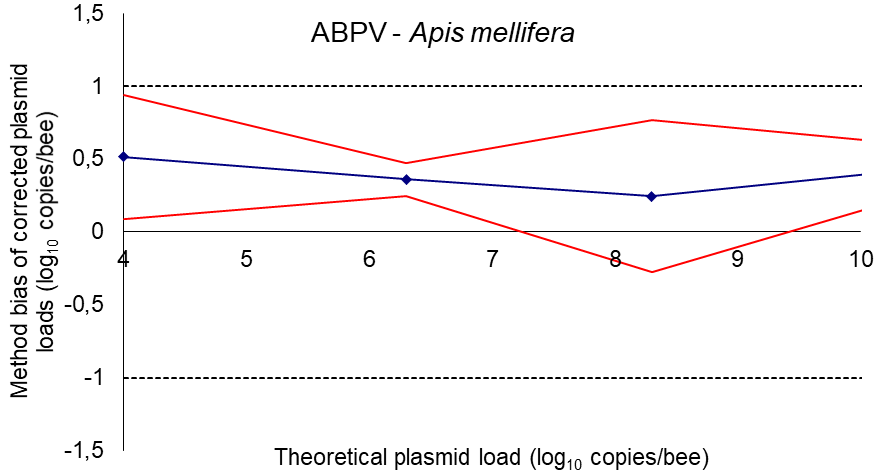

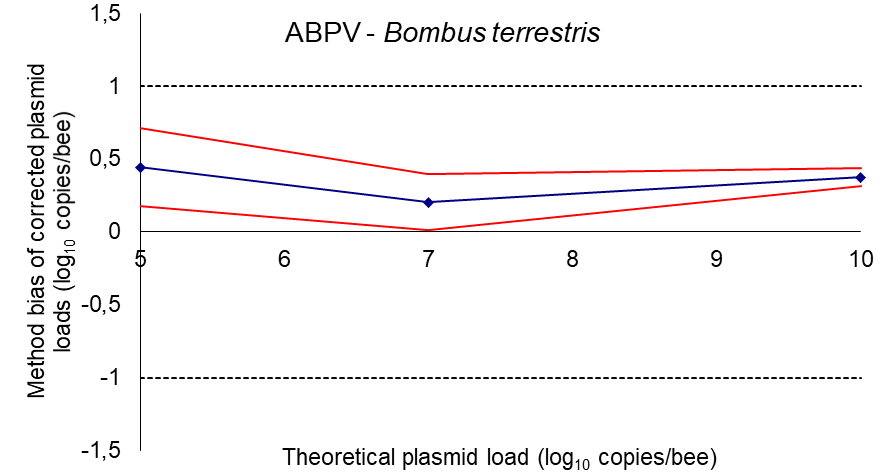

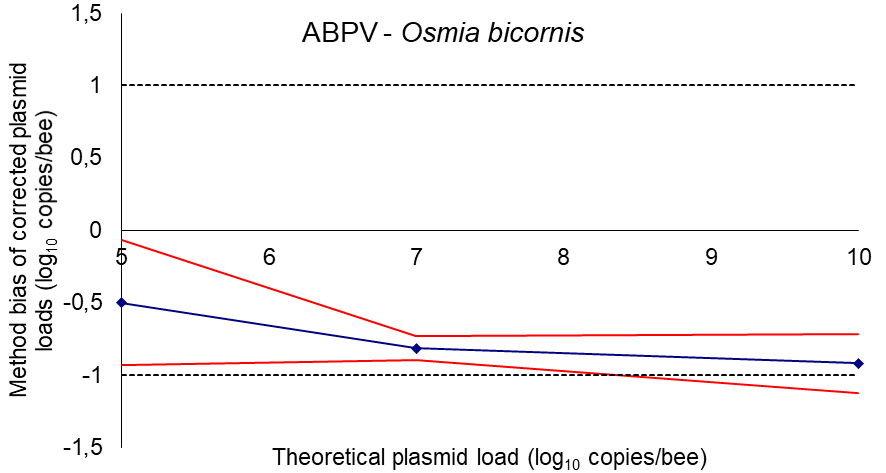


- BQCV


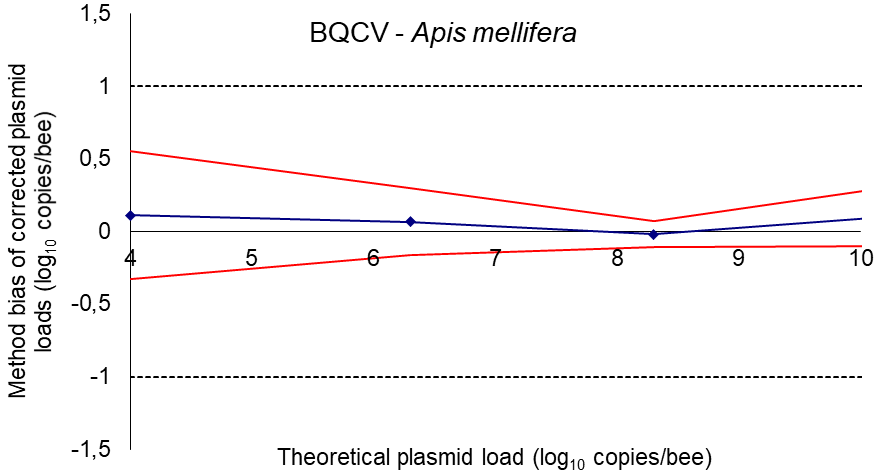

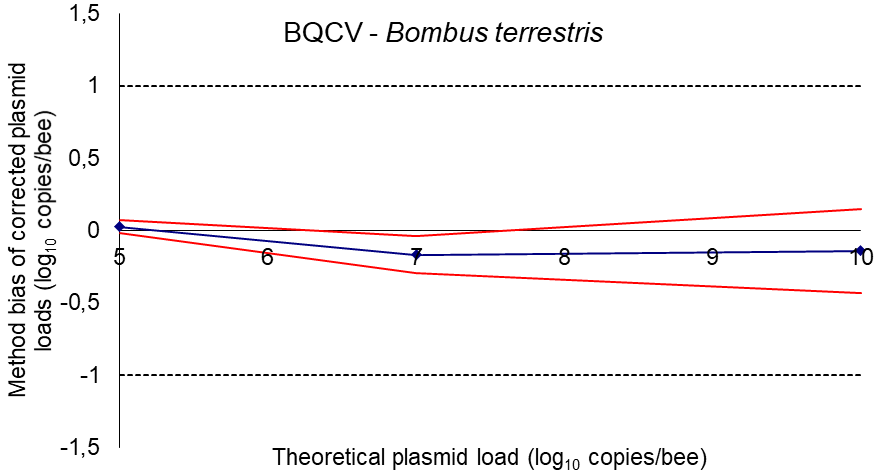

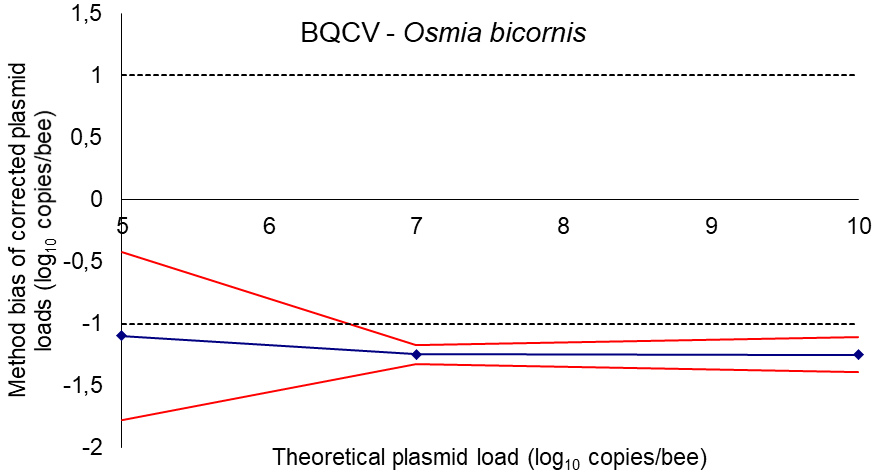


- CBPV


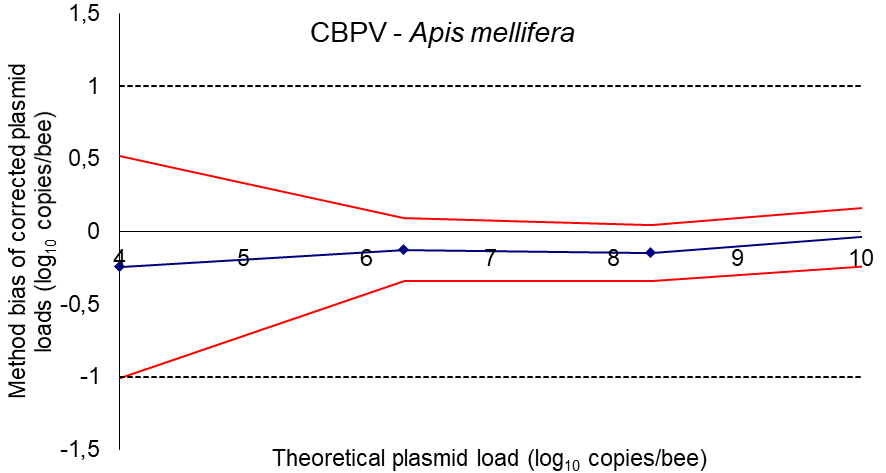

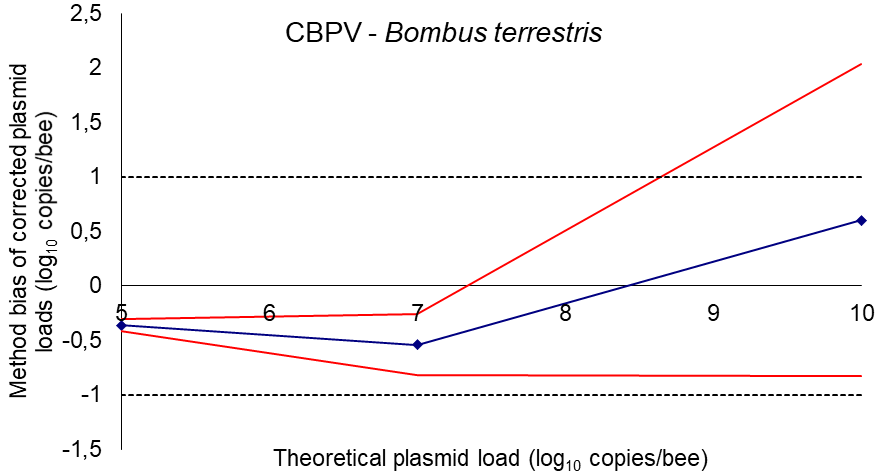

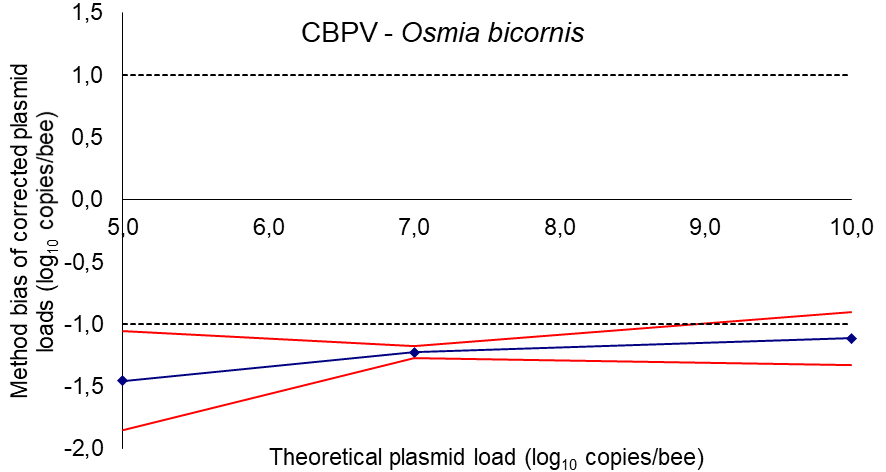


- DWV-A


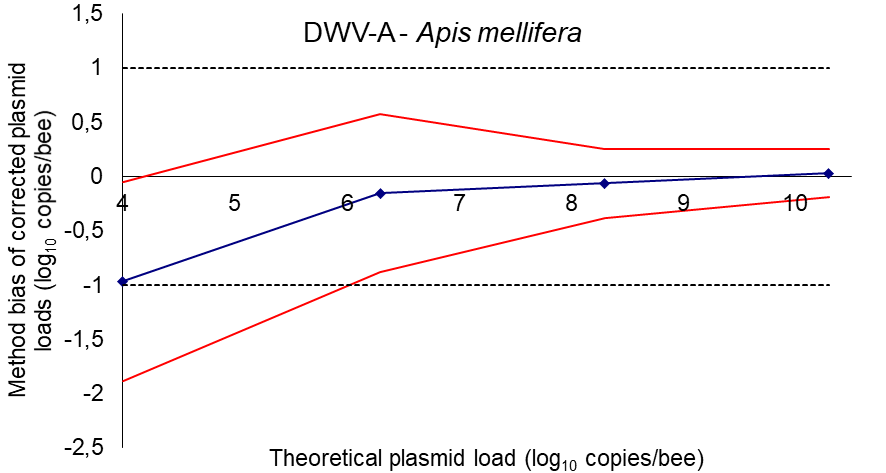

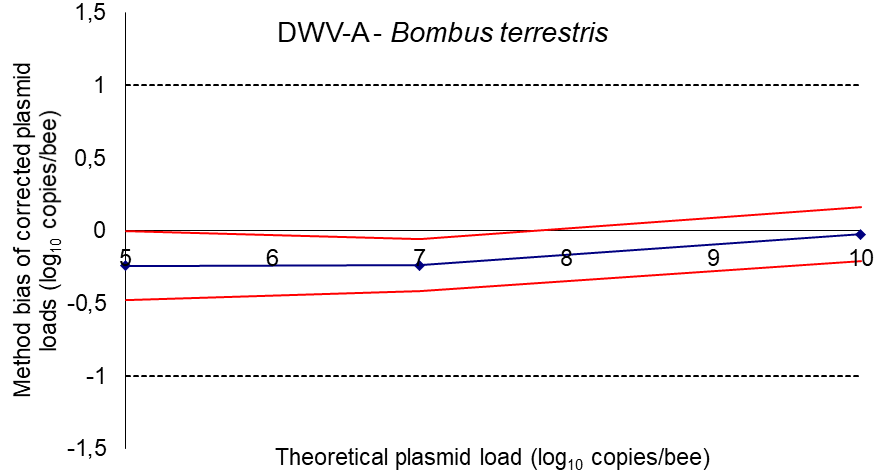

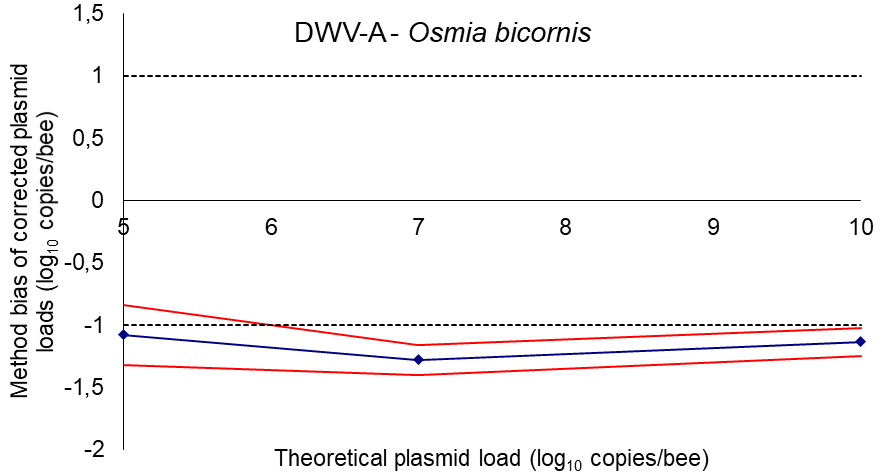


- DWV-B


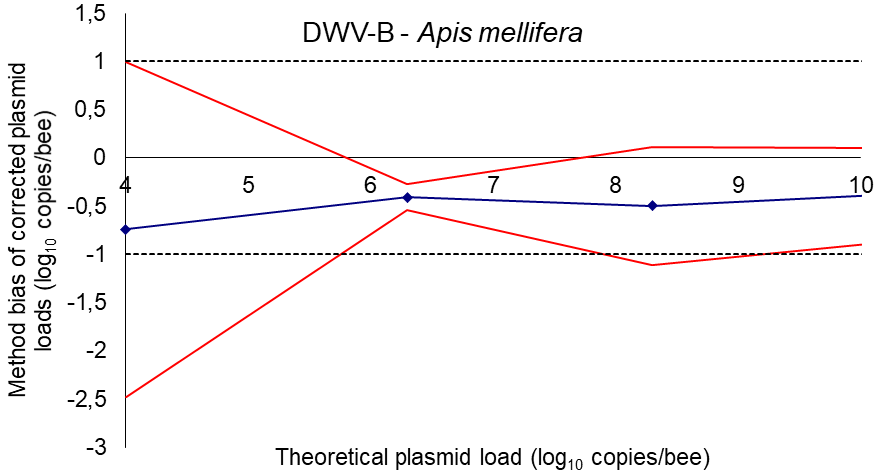

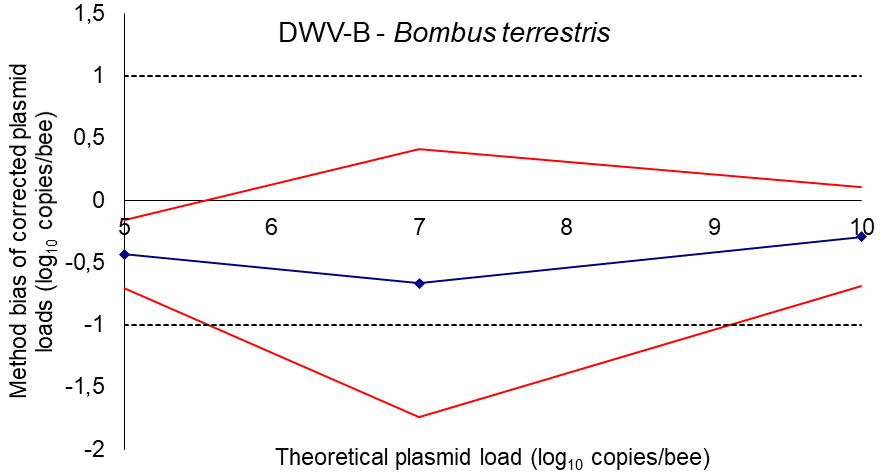

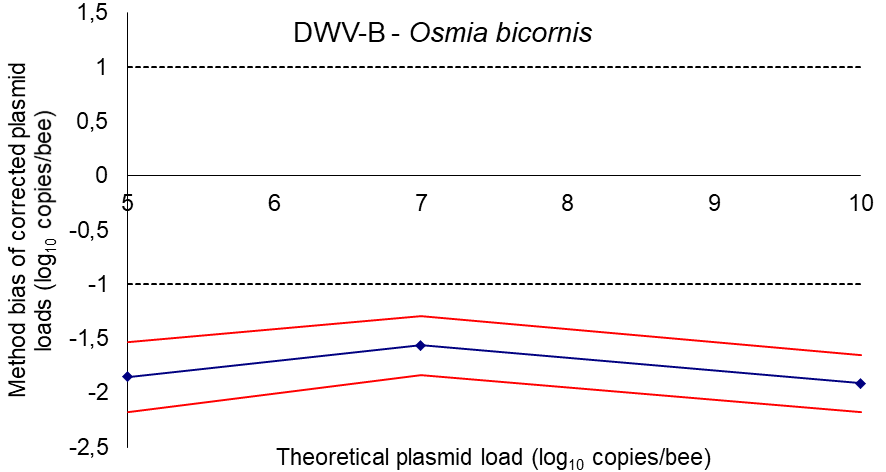


- SBV


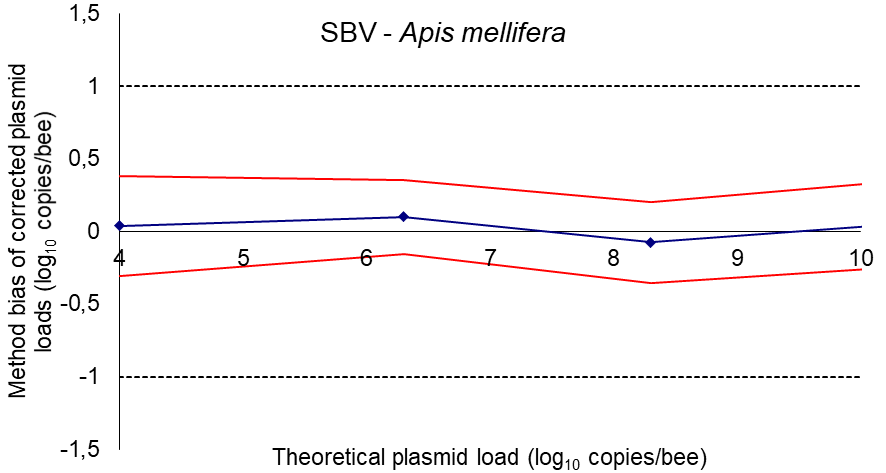

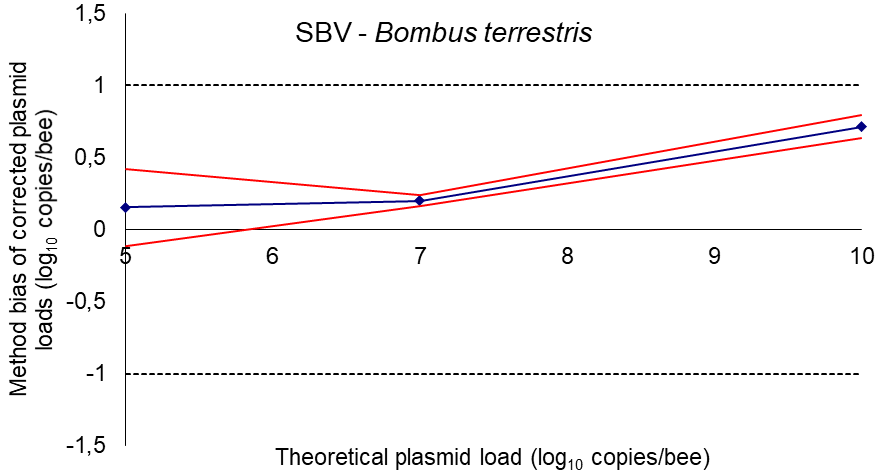

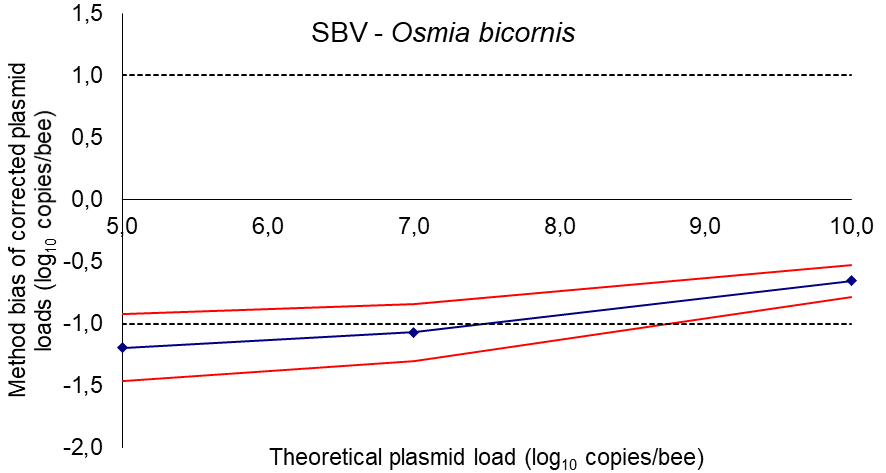


- *Paenibacillus larvae*


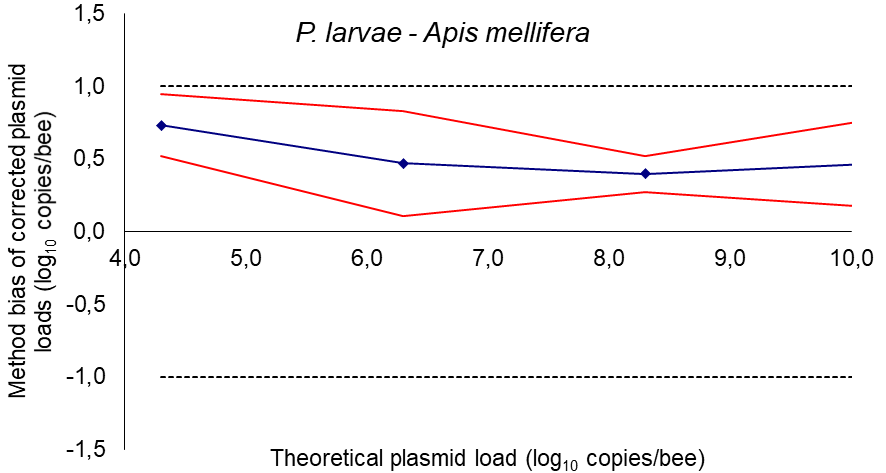

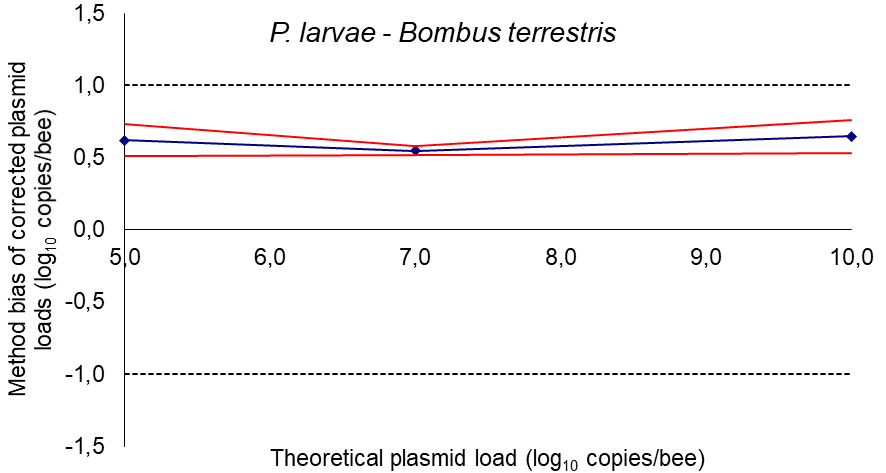

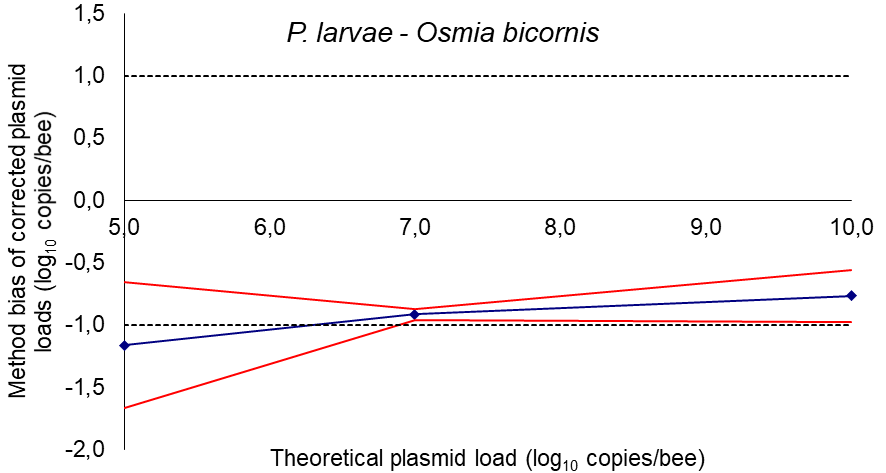


- *Melissococcus plutonius*


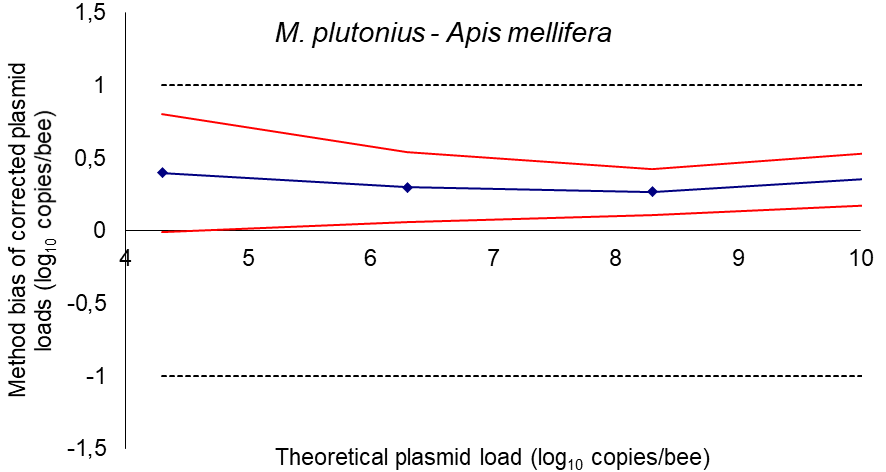

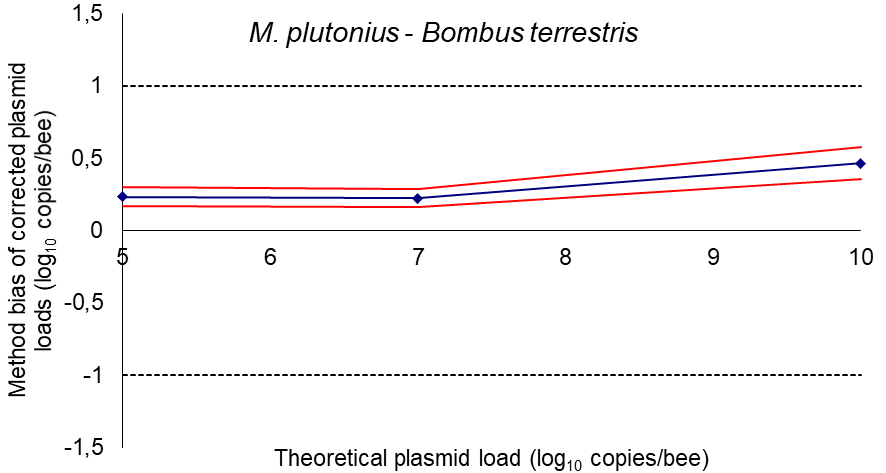

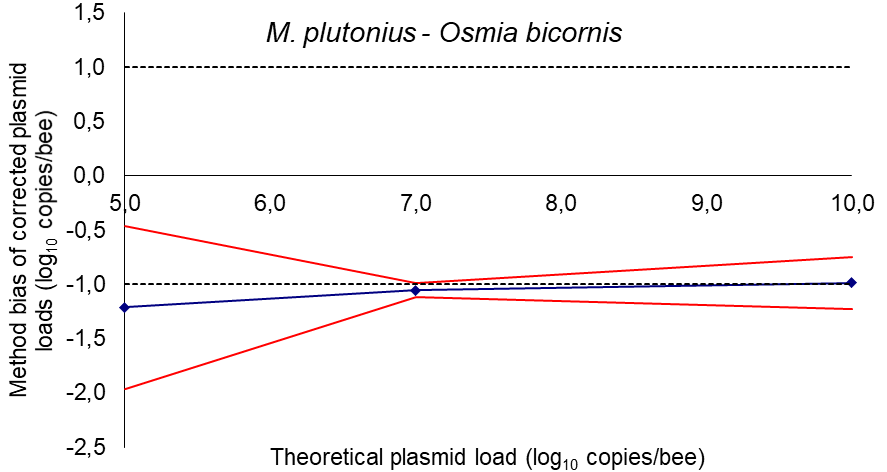


**Supplementary table S29.** Details of the PCR primers and probes used for the detection and quantification of the 11 IPAs.

| **Target IPA** | **Target gene (copy number)** | **Nature** | **Primers and probe (5’-3’)** | **Amplicon size (pb)** | **Source** |
| --- | --- | --- | --- | --- | --- |
| ABPV | Protein  (1 copy) | Virus | Fwd: CATATTGGCGAGCCACTATG  Rev: CTACCAGGTTCAAAGAAAATTTC  Pr: TATCGTATAGCTATAGTTAAAACAGCTT (6FAM, BHQ1^®^) | 91 | Primers: [3]  Probe: this study |
| BQCV | Polyprotein  (1 copy) | Virus | Fwd: GGTGCGGGAGATGATATGGA  Rev: GCCGTCTGAGATGCATGAATAC  Pr: TTTCCATCTTTATCGGTACGCCGCC (6FAM, BHQ1^®^) | 71 | [4] |
| CBPV | Polymerase  (1 copy) | Virus | Fwd: CGCAAGTACGCCTTGATAAAGAAC  Rev: ACTACTAGAAACTCGTCGCTTCG  Pr: TCAAGAACGAGACCACCGCCAAGTTC (6FAM, BHQ1^®^) | 101 | [5] |
| DWV-A | Polyprotein  (1 copy) | Virus | Fwd: GCGGCTAAGATTGTAAATTG  Rev: GTGACTAGCATAACCATGATTA  Pr: CCTTGACCAGTAGACACAGCATC (6FAM, BHQ1^®^) | 72 | [6] |
| DWV-B | Polyprotein  (1 copy) | Virus | Fwd: GGTCTGAAGCGAAAATAG  Rev: CTAGCATATCCATGATTATAAAC  Pr: CCTTGTCCAGTAGATACAGCATCACA (6FAM, BHQ1^®^) | 73 | [6] |
| SBV | Polyprotein  (1 copy) | Virus | Fwd: AACGTCCACTACACCGAAATGTC  Rev: ACACTGCGCGTCTAACATTCC  Pr: TGATGAGAGTGGACGAAGA (6FAM, MGB-Eclipse^®^) | 70 | [7] |
| *Paenibacillus larvae* | rRNA 16S ITS  (8 copies) | Gram+ bacterium | Fwd: CACCTTACGTGTGATTGAAAAC  Rev: TGCTCTTACCAGCTTAACCTAT  Pr: CAGCGAAAGCCCTTCAAAGAAA (6FAM, BHQ1^®^) | 83 | Primers: this study  Probe: EU Reference Laboratory for Honeybee health, pers. com. |
| *Melissococcus plutonius* | rRNA 16S  (4 copies) | Gram+ bacterium | Fwd: TGTTGTTAGAGAAGAATAGGGGAA  Rev: CGTGGCTTTCTGGTTAGA  Pr: AGAGTAACTGTTTTCCTCGTGACGGT (6FAM, BHQ1^®^) | 69 | [8] |
| *Nosema apis* | *RPB1*  (1 copy) | Microsporidium | Fwd: TGCAGATTTTGACGGAGATGA  Rev: TGTACAATACCCATTATAGGACGA  Pr: TGAATTTACACATGCCACAATCA (6FAM, MGB-Eclipse^®^) | 138 | [2] |
| *Nosema ceranae* | *RPB1*  (1 copy) | Microsporidium | Fwd: TCTTGTTCCTCCACCATCAGT  Rev: TGTGTCAAATCATCTTCTGCTCT  Pr: ATCTATTGTTATGGAAGGGATG (6FAM, MGB-Eclipse^®^) | 75 | [2] |
| *Nosema bombi* | *RPB1*  (1 copy) | Microsporidium | Fwd: GGAGAAATCTGTGAAAGTGGGT  Rev: GGCTACTAGTCCCATTCCTTCT  Pr: TGTGGGAATAAACAGCCTGCT (6FAM, BHQ1^®^) | 81 | [2] |

**Supplementary figure S21. Charts of the method positive controls included at the purification step of the molecular analytical workflow.** These positive controls were included in the nucleic acid purification. They consisted of a honey bee homogenate spiked with 7 log_10_ copies/bee of the specific plasmids of the 11 IPAs. These controls went through all the analytical steps up to the qPCR.

For each IPA, analytical results were plotted in control charts to analyse the quantification uncertainty and detect unexpected deviations from the theoretical load 7 log_10_ copies/bee that would indicate analytical issues. Each control chart shows the analytical plasmid loads (blue circles), and the mean load as a function of the increasing number of analysed positive controls (thin black dotted line). Each chart also includes three sets of limits to interpret the results: the monitoring limits (mean load ± 2 sd; yellow lines), the control limits (mean ± 3 sd; red lines) and validation limits (mean load ± 3 sd of intermediate fidelity set at 1 log_10_ copies/bee; thick black dotted lines).

Method uncertainty for each IPA quantification (in log_10_ copies/bee) calculated from plotting the analytical results of 16-17 positive controls. *Pl*: *Paenibacillus larvae*, *Mp*: *Melissococcus plutonius*, *Na*: *Nosema apis*, *Nc*: *Nosema ceranae*, *Nb*: *Nosema bombi.*

|  | ABPV | BQCV | CBPV | DWV-A | DWV-B | SBV | *Pl* | *Mp* | *Na* | *Nc* | *Nb* |
| --- | --- | --- | --- | --- | --- | --- | --- | --- | --- | --- | --- |
| *U* | 0.62 | 0.53 | 0.65 | 0.69 | 0.76 | 0.39 | 1.36 | 1.24 | 0.33 | 0.83 | 0.37 |


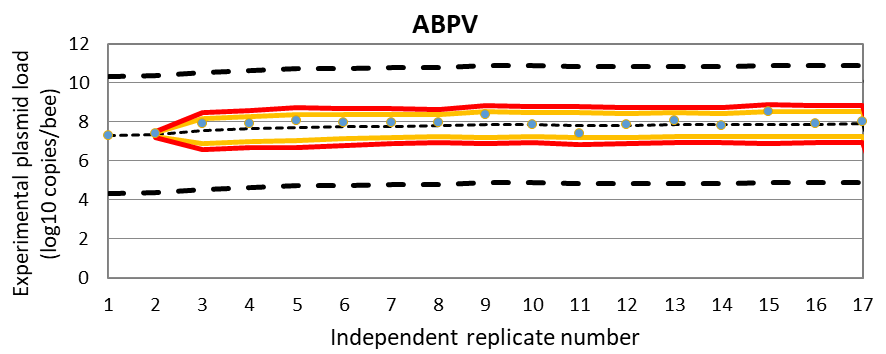

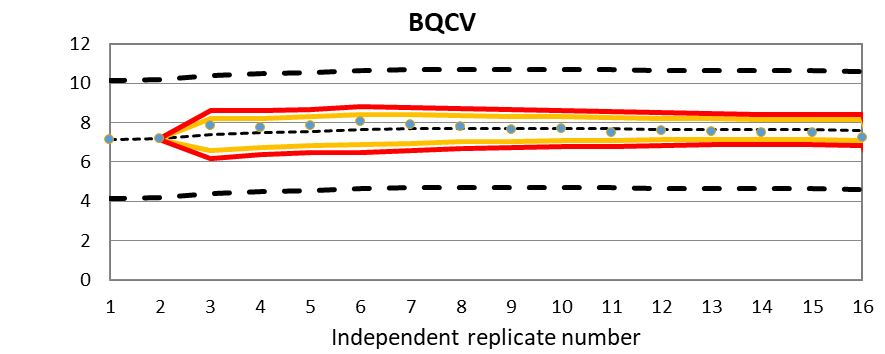

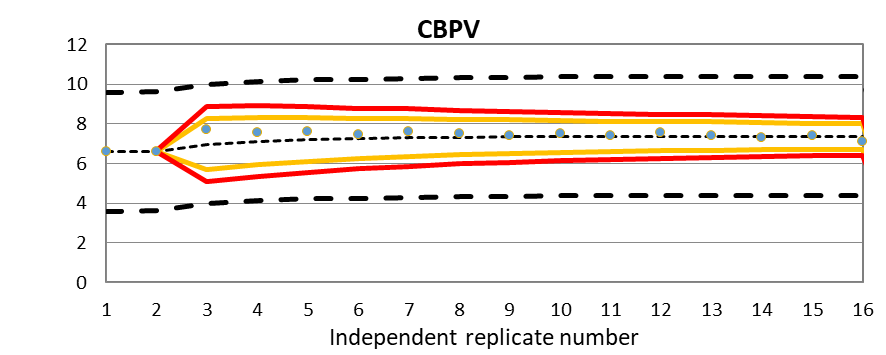

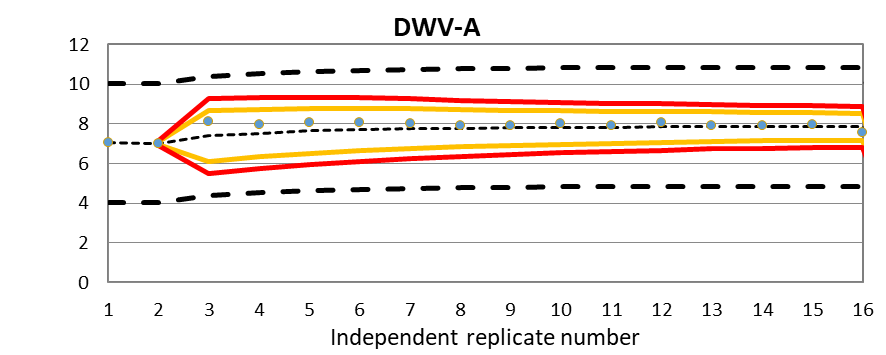

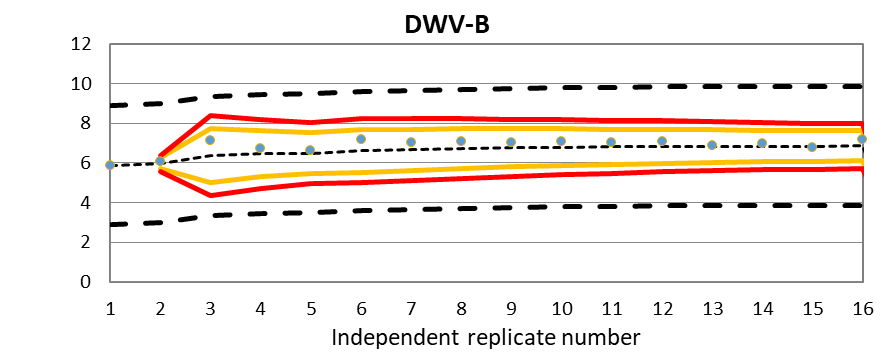

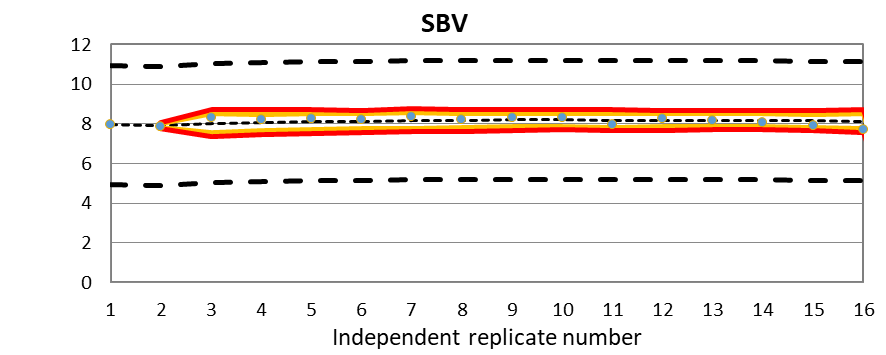

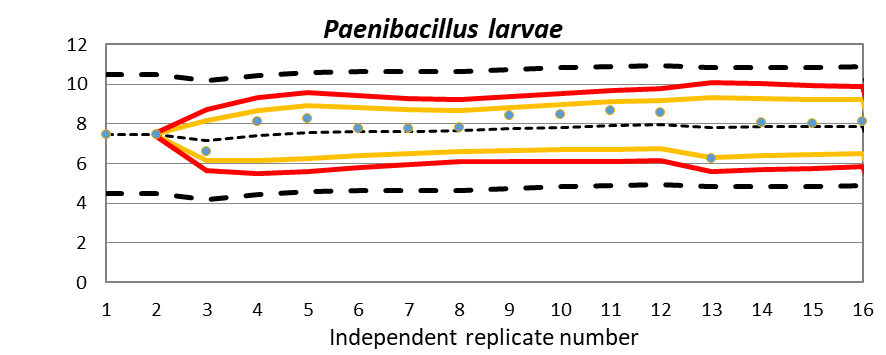

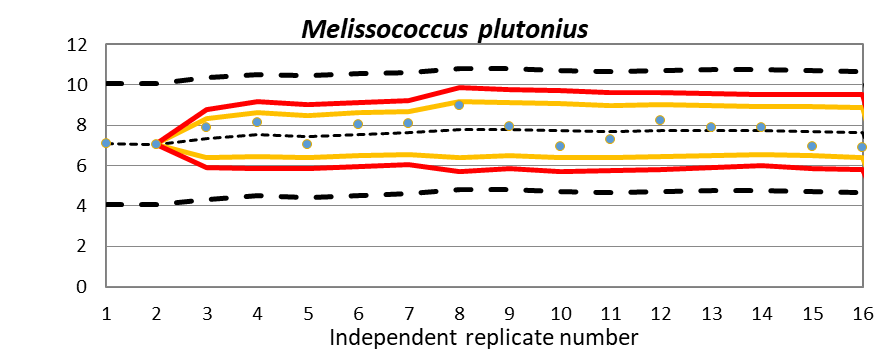


**
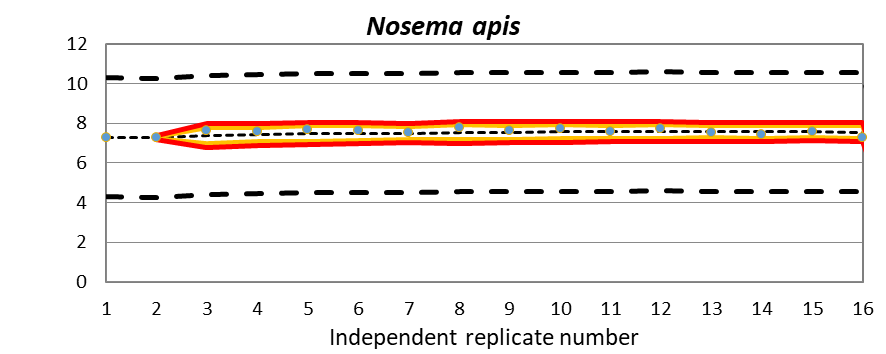
**

**
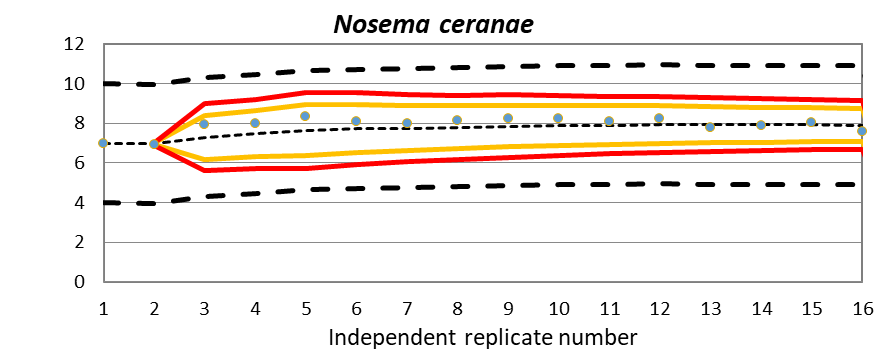
**

**
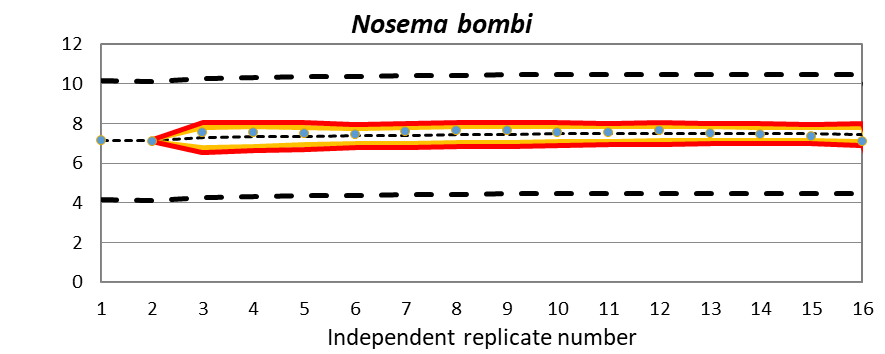
**

**Supplementary table S30. Limit of detection (LD) of the molecular method for the quantification of each IPA.** These limits were used to replace non-detected analytical results in the dataset for the statistical analysis of variance.

| **IPA** | **LD of the method (in log_10_ IPA/bee)** |
| --- | --- |
| ABPV | 4.16 |
| BQCV | 4.85 |
| CBPV | 5.35 |
| DWV-A | 5.73 |
| DWV-B | 5.02 |
| SBV | 5.18 |
| *P. larvae* | 4.57 |
| *M. plutonius* | 3.94 |
| *N. apis* | 5.32 |
| *N. ceranae* | 4.35 |
| *N. bombi* | 4.18 |

**References**

1. AFNOR 2015 NF U47-600, Part 2: Animal health analysis methods – PCR – Part 2: Requirements and recommendations for the development and the validation of veterinary PCR, Norme française. AFNOR, pp. 1-51 (www.afnor.fr).

2. Babin A, Schurr F, Rivière M-P, Chauzat M-P, Dubois E. 2022 Specific detection and quantification of three microsporidia infecting bees, *Nosema apis*, *Nosema ceranae*, and *Nosema bombi*, by probe-based real-time PCR. *Eur. J. Protistol.* 86, 125935 (doi: 10.1016/j.ejop.2022.125935).

3. Jamnikar Ciglenečki U, Toplak I. 2012 Development of a real-time RT-PCR assay with TaqMan probe for specific detection of acute bee paralysis virus. *J. Virol. Methods* 184, 63-68 (doi: 10.1016/j.jviromet.2012.05.010).

4. Chantawannakul P, Ward L, Boonham N, Brown M 2006 A scientific note on the detection of honeybee viruses using real-time PCR (TaqMan) in Varroa mites collected from a Thai honeybee (*Apis mellifera*) apiary. *J. Invertebr. Pathol.* 91, 69–73 (doi: 10.1016/j.jip.2005.11.001).

5. Blanchard P, Regnault J, Schurr F, Dubois E, Ribière M. 2012 Intra-laboratory validation of chronic bee paralysis virus quantitation using an accredited standardised real-time quantitative RT-PCR method. *J. Virol. Methods* 180, 26–31 (doi: 10.1016/j.jviromet.2011.12.005).

6. Schurr F, Tison A, Militano L, Cheviron N, Sircoulomb F, Rivière M-P, Ribière-Chabert M, Thiéry R, Dubois R. 2019 Validation of quantitative real-time RT-PCR assays for the detection of six honeybee viruses. *J. Virol. Methods* 270, 70-78 (doi: 10.1016/j.jviromet.2019.04.020).

7. Blanchard P, Guillot S, Antunez K, Koglberger H, Kryger P, de Miranda JR, Franco S, Chauzat, M-P, Thiéry R, Ribière M. 2014 Development and validation of a real-time two-step RT-qPCR TaqMan^®^ assay for quantitation of Sacbrood virus (SBV) and its application to a field survey of symptomatic honey bee colonies. *J. Virol. Methods* 197, 7–13 (doi: 10.1016/j.jviromet.2013.09.012).

8. Budge GE, Barrett B, Jones B, Pietravalle S, Marris G, Chantawannakul P, Thwaites R, Hall J, Cuthbertson AGS, Brown MA. 2010 The occurrence of *Melissococcus plutonius* in healthy colonies of *Apis mellifera* and the efficacy of European foulbrood control measures. *J. Invertebr. Pathol.* 105, 164-170 (doi: 10.1016/j.jip.2010.06.004).
